# Shark and ray diversity in the Tropical America (Neotropics)—an examination of environmental and historical factors affecting diversity

**DOI:** 10.7717/peerj.5313

**Published:** 2018-07-20

**Authors:** Jorge Domingo Carrillo-Briceño, Juan D. Carrillo, Orangel Antonio Aguilera, Marcelo R. Sanchez-Villagra

**Affiliations:** 1 Palaeontological Institute and Museum, University of Zurich, Zurich, Switzerland; 2 Department of Biological and Environmental Sciences, University of Gothenburg, Gothenburg, Sweden; 3 Gothenburg Global Biodiversity Centre, Gothenburg, Sweden; 4 Laboratorio de Paleoecologia Marinha e Mudanças Globais, Campus de Gragoatá, Universidade Federal Fluminense, Niterói, Rio de Janeiro, Brazil

**Keywords:** Eastern Central Pacific, Chondrichthyans, Neogene, Fossils, Western Central Atlantic

## Abstract

We present the first comprehensive review of the present and past shark and ray diversity in marine waters of Tropical America, examining the patterns of distribution in the Eastern Central Pacific (EP) and Western Central Atlantic (WA) realms. We identified the major regions of diversity and of endemism, and explored the relations to physical variables. We found a strong relationship between shark and ray diversity with area and coastal length of each province. The Tropical Northwestern Atlantic Province is characterized by high diversity and greater occurrence of endemic species, suggesting this province as the hotspot of sharks and rays in Tropical America. The historical background for the current biogeography is explored and analyzed. Referential data from 67 geological units in 17 countries, from both shallow and deep-water habitats, across five time-clusters from the Miocene to the Pleistocene were studied. New data include 20 new assemblages from six countries. The most diverse Neogene and extant groups of shark and ray are Carcharhiniformes and Myliobatiformes, respectively. The differentiation between Pacific and Atlantic faunas goes to at least the middle Miocene, probably related with the increasing closure of the Central American Seaway acting as a barrier. The highest faunal similarity between the assemblages from the EP and the WA at the early Miocene could be related to the lack of a barrier back then, but increased sampling is needed to substantiate this hypothesis.

## Introduction

**Figure 1:**
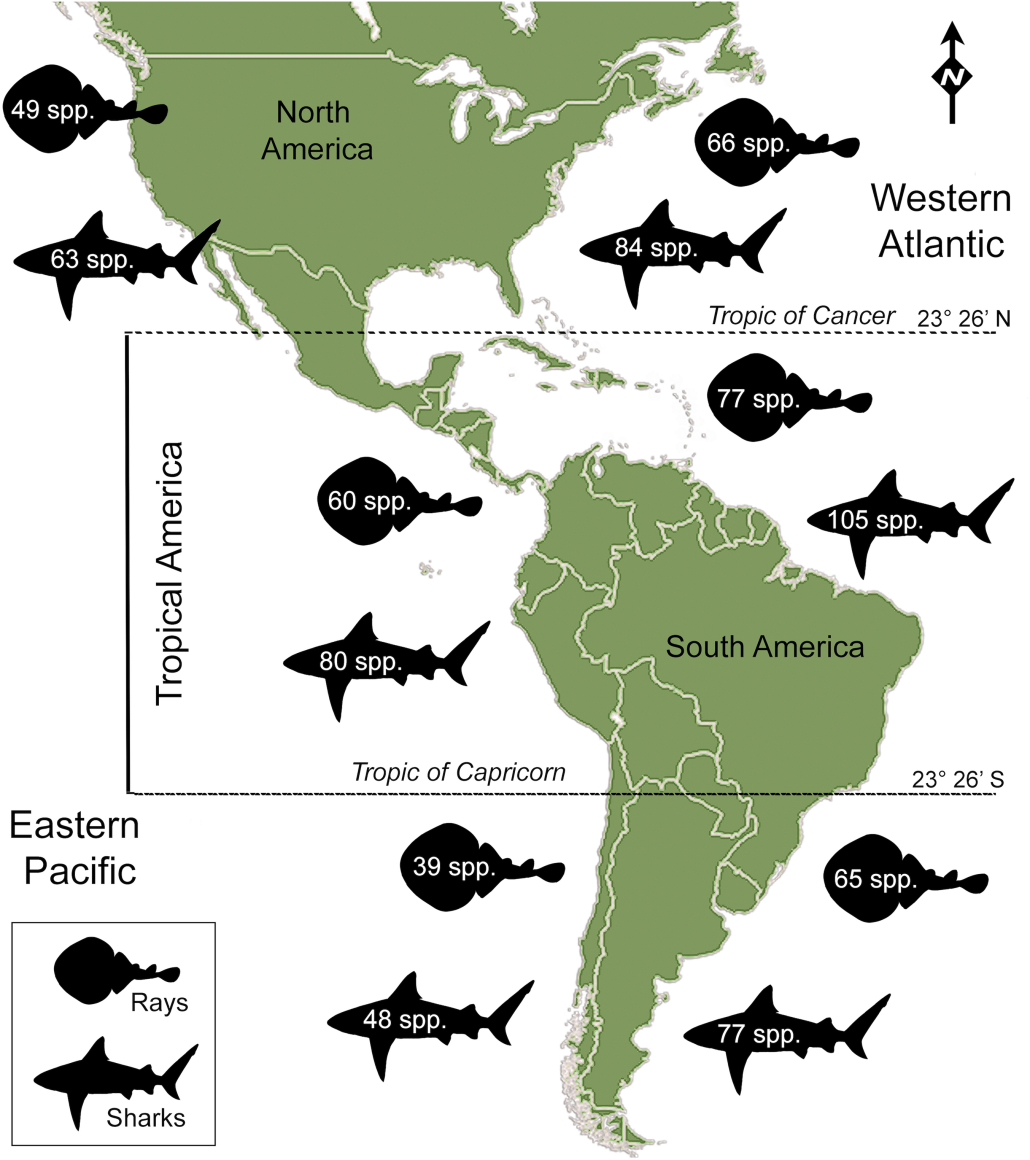
Extant shark and ray species diversity in the Americas. Total number of species is based on the FishBase website (Froese & Pauly, 2017), Ocean Biogeographic Information System (OBIS: http://www.iobis.org/), Table 1; Data S1; Table S2.

Sharks (Selachians) and rays (batoids) are important faunal components of worldwide freshwater and marine ecosystems, with 509 species of sharks and 630 of rays (Weigmann, 2016) playing different functional roles in coastal and oceanic ecosystems (Heithaus et al., 2008; Heithaus, Wirsing & Dill, 2012; Klimley, 2013). In the Americas, much of the diversity is found in tropical coastal seas, along the Atlantic coast (Lucifora, García & Worm, 2011; Miloslavich et al., 2011; Dulvy et al., 2014, fig. 7; Weigmann, 2016; Stein et al., 2018, fig. 1). Current Tropical America’s marine diversity is characterized by 273 species of sharks and rays (Table 1), a number higher than those of the other regions of the Americas (Fig. 1). Although there have been attempts to approach to historical relationship between Miocene ichthyofaunas of Tropical America (e.g., Echeverry & Gallo, 2015), and the temperate Pacific coast of South America (Villafaña & Rivadeneira, 2018), the origin and biogeographic distribution of sharks and rays diversity for the Neogene–Quaternary interval is largely unexplored. Sharks and rays can serve to address major issues on factors driving diversity and the impact of paleontological data to study diversity patterns and geographic distribution in Tropical faunas.

**Table 1:** Extant shark and ray diversity from Tropical America.

Orders	Eastern Central Pacific			Western Central Atlantic			Tropical America		
	Families	Genera	Species	Families	Genera	Species	Families	Genera	Species
**Sharks**									
Hexanchiformes	3	4	4	3	4	5	3	4	5
Echinorhiniformes	1	1	1	0	0	0	1	1	1
Squaliformes	5	12	17	6	11	27	6	16	37
Pristiophoriformes	0	0	0	1	1	1	1	1	1
Squatiniformes	1	1	2	1	1	2	1	1	4
Heterodontiformes	1	1	3	0	0	0	1	1	3
Orectolobiformes	2	2	2	2	2	2	2	2	3
Lamniformes	6	8	11	7	9	12	7	10	14
Carcharhiniformes	5	16	40	7	16	56	7	22	78
**Rays**									
Rhinopristiformes	3	3	8	2	2	5	3	3	11
Rajiformes	3	8	15	4	13	37	4	17	51
Torpediformes	2	4	6	2	6	9	2	6	14
Myliobatiformes	8	12	31	8	12	26	8	13	51
Total	40	72	140	43	77	182	46	97	273

**Note:**

Total numbers are based on Table S2 and Data S1.

Changes in the geomorphological coastal configurations, paleoceanographic patterns and derived currents, and changes in the paleohydrographic systems in the coastal plain basins in Tropical America during the last 23 million years are complex (Coates & Stallard, 2013; Leigh, O’Dea & Vermeij, 2014; O’Dea et al., 2016; Jaramillo et al., 2017). Large scale processes include the closure of the Central American Seaway (CAS) (Woodburne, 2010; Coates & Stallard, 2013; Leigh, O’Dea & Vermeij, 2014; Coates et al., 2004; Montes et al., 2015; Bacon et al., 2015; O’Dea et al., 2016; Jaramillo et al., 2017), and the reduction of neritic areas at the late Pliocene and Pleistocene due to sea level and climatic changes (Miller et al., 2005; De Boer et al., 2010; Pimiento et al., 2017). The CAS was a deep oceanic connection between the Eastern Central Pacific (EP) and Western Central Atlantic (WA) oceans along the tectonic boundary of Caribbean and the South American plates (Jaramillo et al., 2017). The cessation of deep-water flow in the CAS has been hypothesized to be around the middle Miocene ca. 15–12 Ma ago (Montes et al., 2015; Jaramillo et al., 2017), although shallow marine connections (other than CAS) between the Caribbean and Pacific waters likely occurred until the Pliocene, when a permanent land barrier between the Western Pacific and Atlantic oceans was established (Jackson & O’Dea, 2013; Coates & Stallard, 2013). The new “geographic barrier” caused by the closure of the CAS, and the emergence of the isthmus, induced environmental changes on the Pacific and Atlantic oceans, especially in Tropical America, affecting current flow, salinity, temperature, and primary productivity of both sides (Leigh, O’Dea & Vermeij, 2014), launching organisms of the two oceans into independent evolutionary trajectories (Lessios, 2008; Stange et al., 2018). This is evident when examining the current pattern of diversity, which reflects ecological contrasts between the EP and WA (Leigh, O’Dea & Vermeij, 2014). Clear differences in total biodiversity can also be noticed between both oceans areas at the same latitude (Miloslavich et al., 2011). Molecular clock and divergence time estimates in many extant marine species, suggest that high rates of species turnover occurred in EP and WA after their isolation (O’Dea & Jackson, 2009; Lessios, 2008; Leigh, O’Dea & Vermeij, 2014; O’Dea et al., 2016), also providing potential evidence of when the last interoceanic connections were severed (O’Dea et al., 2016). Differential extinction and diversification processes in marine areas of EP and WA have been linked to their contrasting environmental conditions. The habitat loss associated to sea-level oscillations (Miller et al., 2005; De Boer et al., 2010), has also been shown to be associated with marine extinctions at the Pliocene, with a consequent erosion of functional diversity of the marine megafauna (Pimiento et al., 2017). These phenomena have not been studied in sharks and rays, in spite of the availability of direct empirical evidence—fossils—documenting their evolutionary history. Recent studies of the Neogene shark and ray fossil record of Tropical America documented the taxonomy and paleoecology of many assemblages (Aguilera, 2010; Carrillo-Briceño, Aguilera & Rodríguez, 2014; Carrillo-Briceño et al., 2015a, 2015b, 2016a, 2016b; Perez et al., 2017; Aguilera et al., 2017; Landini et al., 2017, and references therein), making the region one of the best documented of the American continent (Kruckow & Thies, 1990; Aguilera, 2010; Aguilera et al., 2011, 2017; Carrillo-Briceño, Aguilera & Rodríguez, 2014; Carrillo-Briceño et al., 2015b, 2016a, and references therein).

We document here the first synthesis of the present and past diversity of sharks and rays in Tropical America, by combining paleontological and neontological occurrence data. We conducted an analytical examination of the physical (environmental) and historical factors that could be associated with such diversity, offering clues about the faunal turnover, extinction patterns, and the origin of endemism and the timing and dynamics of change related to the closure of the CAS.

## Materials and Methods

### Characterization of extant and past shark and ray diversity

We analyzed extant shark and ray fauna diversity across the six marine provinces for Tropical America as defined by Sullivan Sealey & Bustamante (1999) and Spalding et al. (2007, fig. 1). According to Spalding et al. (2007, p. 575); these provinces are “large areas defined by the presence of distinct biotas that have at least some cohesion over evolutionary time frames. Provinces will hold some level of endemism, principally at the level of species.” Each province is characterized by one or more smallest-scale units defined as Marine Ecoregions (Table S1), which also are characterized by a relatively homogeneous species composition, clearly distinct from adjacent systems determined by the predominance of a small number of ecosystems and/or a distinct suite of oceanographic or geomorphologic features (Spalding et al., 2007). The provinces referred for EP are: Tropical Eastern Pacific (TEP), Galapagos (Gal), and Warm Temperate Southeastern Pacific (WTSP); and for the WA: Tropical Northwestern Atlantic (TNWA), North Brazil Shelf (NBS), and Tropical Southwestern Atlantic (TSWA) (see Sullivan Sealey & Bustamante, 1999; Spalding et al., 2007). The WTSP province exceeds the limits of the area that we defined as Tropical America, extending approximately up to 41° S of the Pacific coast of South America. In this circumstance, for the WTSP, we used the extant sharks and rays recorded above the 23°26′S. This area corresponds to the Central Peru and Humboldtian marine ecoregions (Table S1; Spalding et al., 2007, fig. 3). We compiled a distributional database for the extant shark and ray diversity using published data (Table S2); this included the most recent taxonomical status, verification of published faunal lists and regional checklists for these regions (Data S1), information from the FishBase website (Froese & Pauly, 2017), and Ocean Biogeographic Information System (OBIS: http://www.iobis.org/). If at least one record of a taxon was reported for one province, it was assigned as present within the overall marine province. The shark and ray species diversity for the overall studied area was categorized by their prevalent habitat preferences (benthic/demersal, benthopelagic, and pelagic) and habits (coastal, oceanic, and bathyal) (Table S2). The taxonomic nomenclature for the extant and fossil sharks and rays follows mostly Compagno (2005) and Cappetta (2012); for Carcharhiniformes, Rajiformes, Rhinopristiformes sensu Last, Seret & Naylor, 2016, and Aetobatidae sensu, Agassiz, 1858, we follow the nomenclature discussed in Iglésias, Lecointre & Sellos (2005), Last, Weigmann & Yang (2016), Last, Seret & Naylor (2016), and White & Naylor (2016), respectively.

We reviewed the temporal and paleogeographical distribution of the shark and ray assemblages by “Epoch” from the Neogene (Miocene–Pleistocene intervals) fossil record of Tropical America. The data were standardized at the generic level in order to remove biases caused by taxonomic and nomenclatural uncertainties at species level. Although many differences in extant faunas between the EP and WA are at the species level, the resolution in fossil samples is in many cases not at this level, and genera do serve to characterize these faunas while avoiding taxonomic vagaries (Westrop & Adrain, 2001; Eronen et al., 2011). As the known Pliocene and Pleistocene shark and ray assemblages do not present detailed stage/age information, these geological intervals were not subdivided. A comprehensive Miocene–Pleistocene faunal compilation was based on published and new data from 17 countries, encompassing 67 geological units (16 from EP and 51 from WA) from both shallow and deep-water habitats (Tables S3 and S4; Data S2). The new data presented here include the report for eight revised and taxonomically expanded shark and ray fossil assemblages from Costa Rica, Ecuador, Panama, and Venezuela (Table S5), and 20 new assemblages from Colombia, Costa Rica, Panama, Peru, Trinidad, and Venezuela (Table S6). We scored a genus as present in either EP or WA regions, if the genus had at least one record for a given geological interval, indifferently of the geological unit where it comes from.

### Analytical examination of the physical (environmental) factors affecting extant diversity

For the six marine provinces we estimated the total area (km^2^), the length of coastal line (km), and the percent area of bathymetry (categorized in: 0–200, 200–1,000, and >1,000 m depth) (Table S1). We analyzed the correlation between species diversity and these three variables. In order to test the significance of the relationship between each variable with the species diversity, a linear model on the log transformed values was used. All analyses were performed in R (R Core Team, 2017). We used pairwise similarity coefficients to identify faunal similarity among the different marine provinces, using the modified Forbes index (Alroy, 2015). Computer simulations and empirical data suggest that this index is more robust than other similarity coefficients (e.g., Simpson) when sampling size is unequal (Alroy, 2015). We used the R function to estimate the Forbes index, available at http://bio.mq.edu.au/∼jalroy/Forbes.html (accessed 16th of October 2017). We did a hierarchical cluster analysis on the pairwise similarity coefficients using Vegan (Oksanen et al., 2017) and used the average distance algorithm.

### Analytical examination of the paleodiversity

We estimate paleodiversity curves of sharks and rays in Tropical America using the “sampled in bin” and “boundary crossers” methods (Foote, 2000). The sampled in bin method counts the taxa within each time interval and assumes taxa recorded in each interval co-existed (time averaging). The boundary crossers method is more conservative and counts only taxa known before and after the boundary of adjacent time intervals, therefore assuring co-existence of taxa (see Quental & Marshall, 2010). In order to evaluate shark/ray assemblage similarities during Miocene–Pleistocene intervals, the associations from both EP and WA were compared using a dissimilarity analysis, as described above. We used five epoch intervals: early Miocene (Emi), middle Miocene (Mmi), late Miocene (Lmi), Pliocene (P), and Pleistocene (Pl). Finally, we estimated extinction rates for sharks from the middle Miocene to the Pleistocene, using the boundary crossers rate estimate (Foote, 2000). There is insufficient data to estimate rates prior the middle Miocene. For all the analyses, we assume that a genus is present for the entire interval between its first and last known occurrence in either EP or WA (the range-through assumption, Foote & Miller, 2007) (Table S4).

## Results

### Extant shark and ray diversity

**Figure 2:**
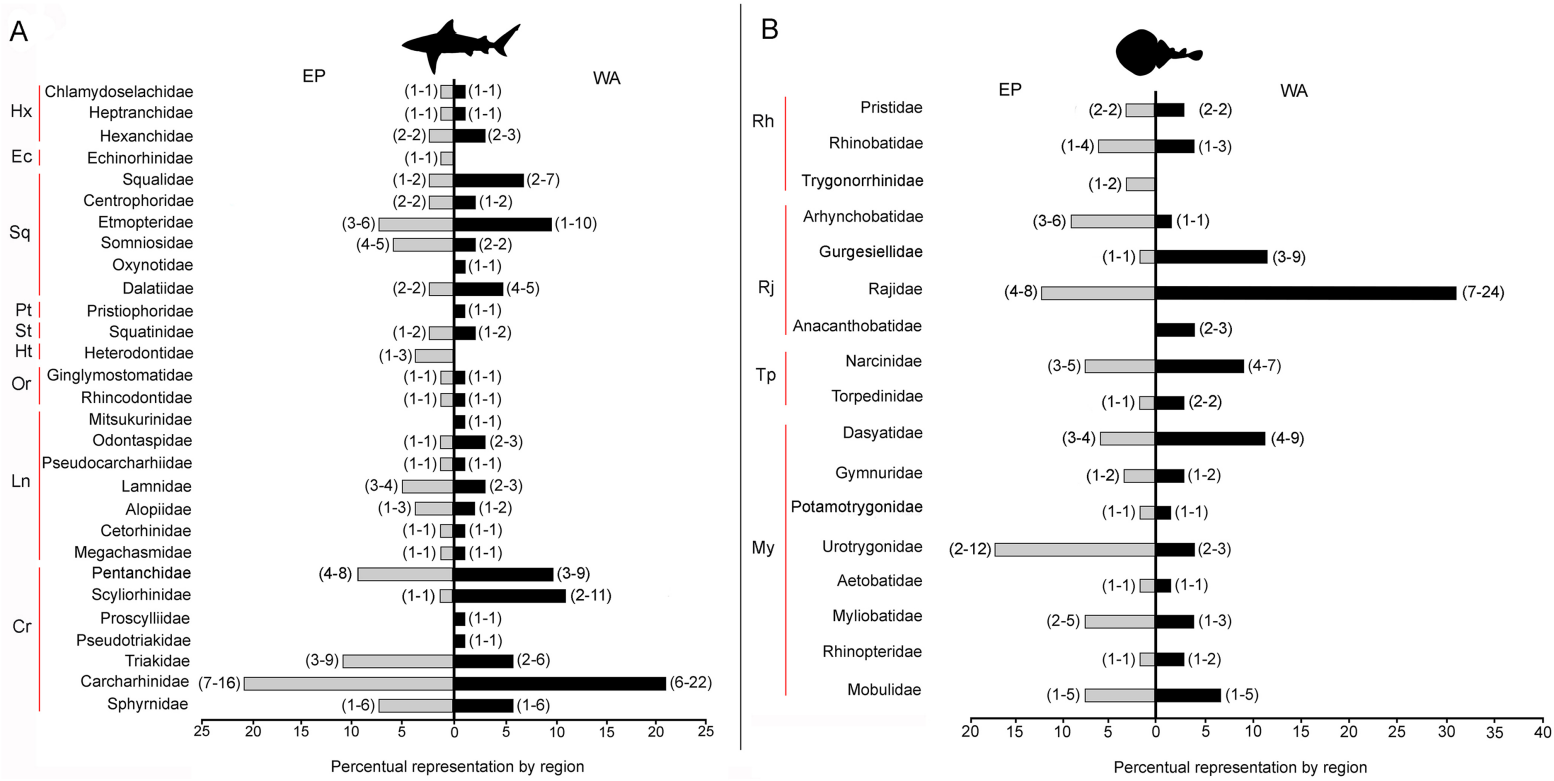
Tropical America extant shark and ray diversity by families and regions. (A) Sharks. (B) Rays. Gray bars: Eastern Central Pacific “EP”; black bars: Western Central Atlantic “WA”. Values in parentheses represent total genera (first entry) and total species (second entry). Study sites: Hx, Hexanchiformes; Ec, Echinorhiniformes; Sq, Squaliformes; Pt, Pristiophoriformes; St, Squatiniformes; Ht, Heterodontiformes; Or, Orectolobiformes; Ln, Lamniformes; Cr, Carcharhiniformes; Rh, Rhinopristiformes; Rj, Rajiformes; Tp, Torpediformes; My, Myliobatiformes. Information based on Table S2.

**Figure 3:**
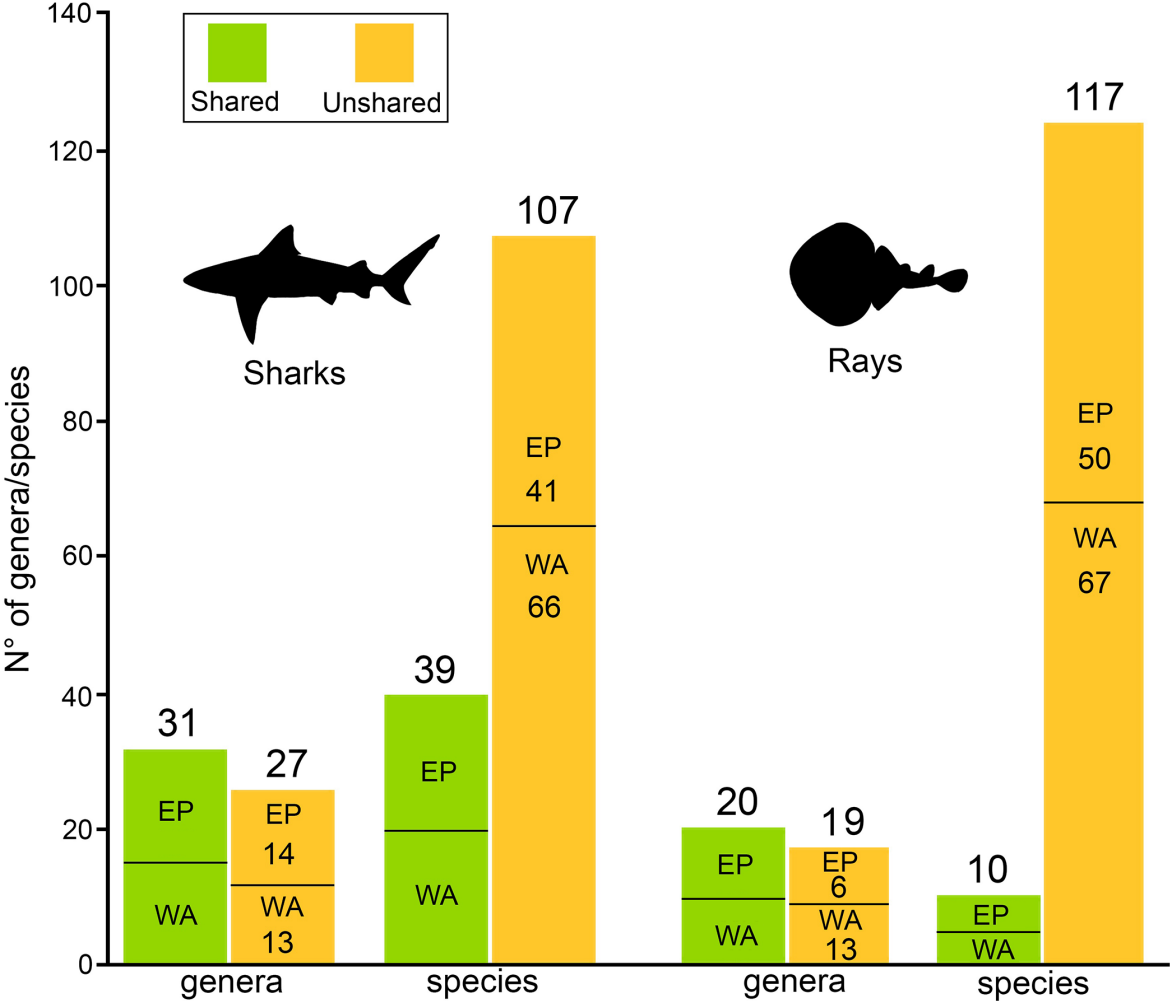
Shared/unshared shark and ray genera and species in Eastern Pacific and Western Atlantic sides of Tropical America.

There are 146 sharks and 127 rays species in Tropical America, belonging to 46 families (29 sharks and 17 rays) and 97 genera (58 sharks and 39 rays), whereby Carcharhiniformes, Rajiformes and Myliobatiformes are the most diverse groups of sharks and rays, respectively (Fig. 2; Table 1). A conservative account of endemic species for the region is 25 species of sharks (EP: 4 spp.; WA: 21 spp.) and 30 species of rays (EP: 15 spp.; WA: 15 spp.) (Table S7). There is a marked difference in diversity between EP and WA faunas (Fig. 1). For EP, 80 and 60 species of sharks and rays, respectively, are reported, while 105 sharks and 77 rays inhabit WA (Table 1). A total of 39 sharks and 10 ray species are present in EP and WA (Fig. 3), representing 49% of shark species in the EP and 37% in the WA, and 17% of the ray species in the EP and 13% in the WA. There are a similar number of shark and ray genera in the two regions, with 45 shark and 27 ray genera in the EP, and 44 shark and 33 ray genera in the WA (Table 1). A total of 31 shark and 20 ray genera is present in both regions (Fig. 3), representing the 69% of the shark genera in the EP and 70% in WA, and the 74% of the ray genera in the EP and the 61% in the WA. A total of 22 shark families is shared between EP and WA, whereas two are present only in the EP (Echinorhinidae and Heterodontidae), and five only in the WA (Oxynotidae, Pristiophoridae, Mitsukurinidae, Proscylliidae, and Pseudotriakidae) (Fig. 2). A total of 15 ray families is shared between EP and WA, while Trygonorrhinidae is present only in the EP and the Anacanthobatidae only in the WA (Fig. 2).

**Figure 4:**
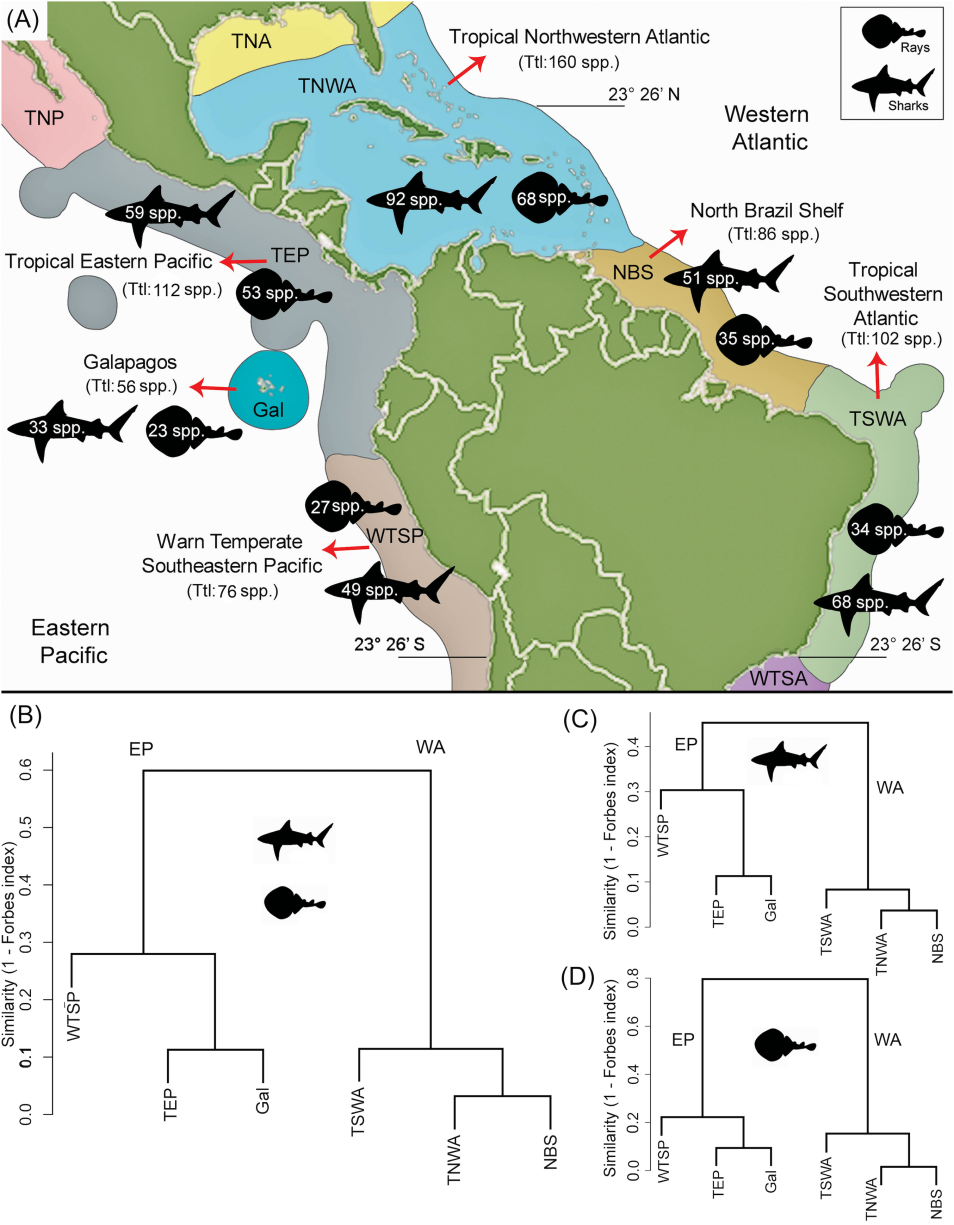
Shark and ray species diversity and similarity analysis among the six marine provinces of Tropical America. (A) Diversity by marine provinces. (B) Similarity analysis for sharks and rays. (C) Similarity analysis for sharks only. (D) Similarity analysis for rays only. Our data for the WTSP province, includes only the shark and ray diversity recorded for the Central Peru and Humboldtian marine ecoregions (approximately up to 23°26′S) (Table S1; Spalding et al., 2007, fig. 3). Study sites: EP, Eastern Central Pacific; WA, Western Central Atlantic; TNP, Temperate Northern Pacific; TNA, Temperate Northern Atlantic; WTSA, Warm Temperate Southwestern Atlantic.

There is a higher diversity of shark genera and species over rays in all marine provinces (Fig. 4A; Table 1). The TNWA province (in the WA) is the most diverse, with a total of 160 species, where 92 correspond to sharks and 68 to rays. This province also has the highest number of endemic taxa with 28 species (16 of sharks and 12 of rays). The second most diverse province is the TEP (in the EP) with a total of 112 species, 59 sharks 53 rays. Of these, 14 species (two of sharks and 12 of rays) are endemic to this province (Table S7). The less diverse province is Gal in the EP, with a diversity of 56 species (33 of sharks and 23 of rays).

### Shark and ray fossil diversity

**Figure 5:**
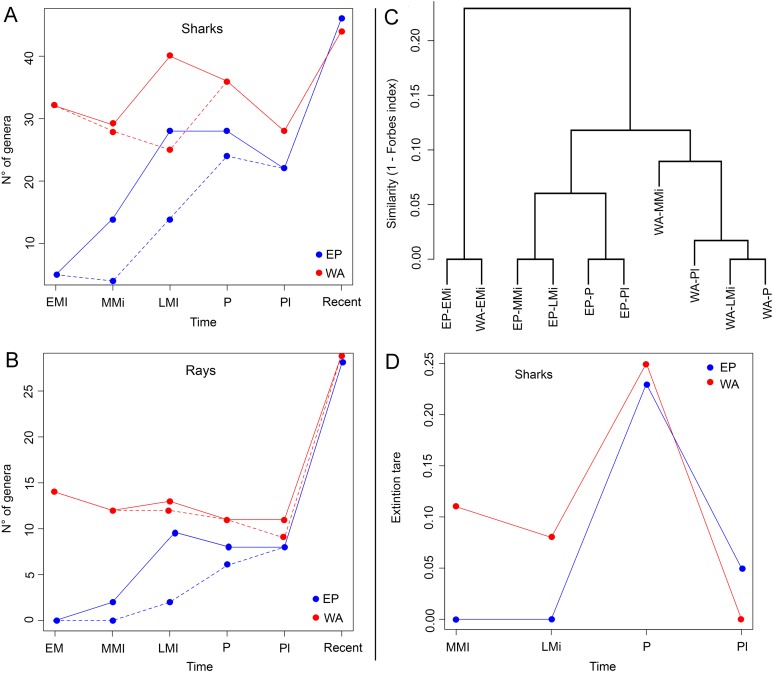
Paleodiversity, faunal similarity, and extinction rates of sharks and rays in Tropical America. Paleodiversity was estimated using the sampled in bin method (solid line) and the boundary crossers method (dashed line) for the Eastern Central Pacific (EP) and Western Central Atlantic (WA). (A) Paleodiversity of shark genera. (B) Paleodiversity of ray genera. (C) Faunal similarity cluster analysis of the early Miocene–Pleistocene shark and ray assemblages. (D) Extinction rates values of shark genera, estimated with the boundary crosser method. Study sites: EMi, early Miocene; MMi, middle Miocene; LMi, late Miocene; P, Pliocene; PI, Pleistocene.

The Miocene–Pleistocene shark and ray faunal compilation of published (Data S2) and new data presented here (Tables S3–S6) for Tropical America is represented by 69 genera (52 of sharks and 17 of rays) (Figs. 5A and 5B; Table 2; Table S3). The WA has the greater fossil diversity of the region, with 63 genera, of which 46 are sharks (Carchariniformes and Lamniformes are the most diverse groups), and 17 are rays (Myliobatiformes is the most diverse group). In contrast, in the EP there are 44 genera, 33 sharks (Carchariniformes and Lamniformes being the most diverse groups), and 11 rays (Myliobatiformes the most diverse group), and the low paleodiversity in the assemblages of the early and middle Miocene intervals (Table 2), are related with the biases in the fossil record of this region. There are few records of fossil sharks and rays for the Pleistocene. However, for this time period, 23 genera of sharks and nine of rays are inferred to be present in EP, and 29 shark and 12 ray genera are inferred to be present in WA (Table 2), because they are recorded in the Pliocene and the present (Figs. 5A and 5B). Our analysis reveals extirpation/extinction patterns, where at least 29 genera (24 of sharks and five of rays) were affected during Neogene time in the Americas, of which: (1) four shark genera (*Dalatias*, *Pristiophorus*, *Carcharias*, and *Isogomphodon*) regionally extirpated from the EP currently live in the WA; (2) two shark (*Deania* and *Heterodontus*) and two ray genera (*Taeniurops* and *Aetomylaeus*) were regionally extirpated from the WA, but with extant representatives in EP; (3) nine shark (*Trigonognathus*, *Scymnodon*, *Chiloscyllium*, *Nebrius*, *Iago*, *Chaenogaleus*, *Hemipristis*, *Paragaleus*, and the Stegostomatidae family) and two ray (*Rhynchobatus* and *Taeniura*) genera became completely extirpated from the Americas; (4) nine shark (†*Carcharoides*, †*Cosmopolitodus*, †*Carcharocles*, †*Paradoxodon*, †*Paratodus*, †*Anotodus*, †*Pachyscyllium*, †*Kruckowlamna*, and †*Physogaleus*) and one ray (†*Plinthicus*) genera, also became extinguished from the Americas and worldwide at the end of the Neogene. The extinction rates estimated for shark genera are relatively low; being higher for the Pliocene in both the EP and WA (Fig. 5D). The extinction rates estimated for Tropical America are higher in the WA than in the EP, with the exception of the time interval between the Pleistocene and the present.

**Table 2:** Miocene–Pleistocene shark/ray diversity from Tropical America.

Orders	Eastern Central Pacific						Western Central Atlantic					
	EMi	MMi	LMi	P	Pl	Recent	EMi	MMi	LMi	P	Pl	Recent
**Sharks**												
Hexanchiformes		1	1	2	2	4	1	1	3	3	3	4
Echinorhiniformes				1	1	1						
Squaliformes		1	2	2	2	12	5	6	8	8	6	11
Pristiophoriformes		1	1	1			1	1	1	1	1	1
Squatiniformes			1	1	1	1			1	1	1	1
Heterodontiformes		1	1	1	1	1	1	1	1	1		
Orectolobiformes			1	1	1	2	2		2	2	2	2
Lamniformes	2	3	9	7	4	8	11	9	8	7	5	9
Carcharhiniformes	3	7	13	12	11	16	10	10	14	13	11	16
**Rays**												
Rhinopristiformes			3	1	1	3	2	2	2	2	2	2
Rajiformes				1	1	8			1	1	1	13
Torpediformes						4					1	6
Myliobatiformes		2	7	7	7	12	12	10	10	9	8	12
Total	5	16	39	37	32	72	45	40	51	48	41	77

**Note:**

Total numbers are based on Table S4.

### Physical factors affecting extant diversity

**Figure 6:**
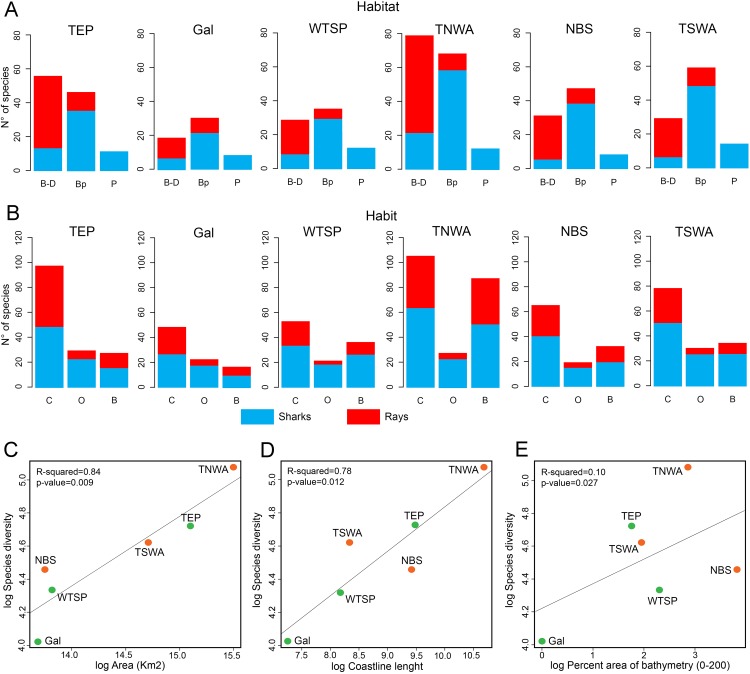
Physical factors affecting extant shark and ray diversity in Tropical America by marine provinces. (A) Habitat. (B) Habit. (C) Area vs. species diversity. (D) Coastline vs. species diversity. (E) Bathymetry vs. species diversity. In (C–E), the green color indicates the marine provinces from EP and the orange are those from the WA. Study sites: B–D, Benthic/demersal; Bp, benthopelagic; P, pelagic; C, coastal; O, oceanic; B, bathyal; TEP, Tropical Eastern Pacific; Gal, Galapagos; WTSP, Warm Temperate Southeastern Pacific; TNWA, Tropical Northwestern Atlantic; NBS, North Brazil Shelf; TSWA, Tropical Southwestern Atlantic.

Shark diversity from the six marine provinces of Tropical America is dominated by benthopelagic species, whereas pelagic and benthic/demersal ones are less diverse (Fig. 6A). Most ray species are benthic/demersal, followed by benthopelagic ones. Concerning habitat preferences, the shark diversity is dominated by coastal species, followed by those associated to bathyal and oceanic environments (Fig. 6B). The ray diversity is also mostly from coastal areas, followed by species of bathyal preferences.

The species diversity and total area of each marine province of Tropical America are positively correlated, being the TNWA (WA) and TEP (EP) the largest and most diverse marine provinces (Fig. 6C). The coastal length has also a positive correlation with the diversity (Fig. 6D). The provinces with longer coast length and greater diversity are the TNWA and the TEP, followed by the TSWA and WTSP. The NBS province has a relative low diversity given its coastal length (the third longest coastline). The area and coastal length are highly correlated (Pearson correlation coefficient = 0.90). There is no correlation between the shark and ray diversity and the percent of neritic areas (0–200 m depth; Fig. 6E).

### Similarity analysis of extant and fossil sharks and rays

The similarity analysis of extant shark and ray faunas among the six marine provinces clearly differentiates the EP and WA regions (Fig. 4B). The fauna similarity analysis in the EP shows a closer similarity between the TEP and Gal. For the WA side, it shows a closer similarity between the TNWA and NBS provinces. These overall patterns are similar in separated analyses of sharks (Figs. 4C and 4D; Table S8).

There is a high similarity between the early Miocene assemblages from the EP and WA (Forbes index = 1; Fig. 5C)—notwithstanding the limited sampling in the EP for the early Miocene—whereas the similarity values between the two regions are lower in younger time intervals: 0.89 for middle Miocene, 0.89 for late Miocene, 0.90 for Pliocene, and 0.91 for Pleistocene (Table S9). For the EP, the time between the early and middle Miocene was the one with the highest taxonomic turnover (lower similarity value, Forbes index = 0.82), whereas for the younger time intervals the similarity was 1.00 between the middle and late Miocene, 0.95 between late Miocene and Pliocene, and 1.00 between Pliocene and Pleistocene. For the WA, we obtained high Forbes similarity values for adjacent time periods: 0.99 between the early and middle Miocene, 0.96 between middle and late Miocene, 0.99 between the late Miocene and Pliocene, and 0.98 between the Pliocene and Pleistocene.

## Discussion

### Distributional patterns and factors affecting extant diversity

In Tropical America shark and ray faunas consist mostly of coastal and benthopelagic species (Figs. 6A and 6B). It is well known that habits influence the distribution of sharks and rays (Musick, Harbin & Compagno, 2004), so differences among areas of the EP and WA are expected. Navia, Mejía-Falla & Hleap (2016) noticed that diversity of sharks and rays for marine waters of Colombia, is higher in the Caribbean side (11% more species), than in the Pacific one. There is a lower shark and ray species diversity in the EP than in WA (80 shark and 60 ray species in EP vs. 105 shark and 77 ray species in WA). This pattern is consistent with that reported for many clades of bony fishes (Robertson & Allen, 2015; Miloslavich et al., 2011), where the marine provinces of the EP are less diverse than those of the WA, although the opposite pattern has also been reported, as for the Sciaenidae (Robertson & Allen, 2015; Robertson & Tassell, 2015). Differences in physical conditions, total area and habitat are related to the lower diversity of shorefish species in the TEP, in comparison with the TNWA (also known as “Wider Caribbean”) (Robertson & Allen, 2015). At the genus level, the shark and ray diversity in the EP (45 shark and 27 ray genera) and WA (44 shark and 33 ray genera) is very similar.

There is a contrast between the number of shark and ray species shared between the EP and WA. A relatively high percentage of shark species are shared (49% for EP and 37% for WA), whereas the percentage is lower in the case of ray species (17% for EP and 13% for WA). This difference is not observed at the genus level, where the percentage of shark genera shared between the regions is 69% for EP and 70% for WA, and the percentage of ray genera present in both, EP and WA, is 74% and 61%, respectively. Total area and coastal line are correlated and both have a positive relationship with shark and ray diversity in Tropical America (Figs. 6C and 6D). The TEP and TNWA are the marine provinces from the EP and WA, respectively, with the largest total area and coastline, and therefore they show the highest diversity of sharks and rays in the region. Abiotic variables such as water temperature, salinity, turbidity, and dissolved oxygen concentration, play an important role in the spatial distribution and ecology of sharks and rays (Schlaff, Heupel & Simpfendorfer, 2014, and references therein).

The shark and ray diversity differences among provinces in the EP (Fig. 4A) mirror the environmental features of these areas. The EP provinces are characterized by a narrow continental shelf, where deep-water environments (e.g., depths >1,000 m) are dominant in the total area of bathymetry in the region (Table S1). The TEP province has the largest total area and coastline among the three provinces of the tropical EP, being characterized mainly by coastal habitats, with a low quantity of offshore islands and a wide range of sea surface temperatures that can drop easily due to coastal upwelling (Sullivan Sealey & Bustamante, 1999). This province also has large areas covered of mangroves and the higher river discharges of the region, leading to the largest concentration of estuarine systems with high freshwater outflows of the tropical EP (Sullivan Sealey & Bustamante, 1999; Chatwin, 2007; Miloslavich et al., 2011). The conditions of the TEP province, contrast with those found in the Gal and WTSP, especially in the latter where waters are notably cooler (sea surface temperature maximum is 16–17 °C), the coastline has few geographic accidents and with few or absent river discharges due the scarce continental rain that characterized the region (Sullivan Sealey & Bustamante, 1999; Miloslavich et al., 2011). However, WTSP is part of one of the major upwelling systems of the world (Thiel et al., 2007). The cooler conditions in the most southern regions of the tropical EP could affect shark and ray diversity; sea surface temperature is known to have a direct effect on the physiology and distribution of sharks and rays (Schlaff, Heupel & Simpfendorfer, 2014). The high biodiversity and endemism in terrestrial and marine faunas of the Gal province are well documented (Sullivan Sealey & Bustamante, 1999; Parent, Caccone & Petren, 2008). The Gal province is the southernmost limit of the coral reef formations along the EP (Sullivan Sealey & Bustamante, 1999; Glynn et al., 2015). However, the shark and ray diversity in the Gal province is the lowest diverse of the tropical EP provinces, which could be the result of its small size and isolation. Despite this isolation, Gal has the lowest number of endemic shark and ray species (2) (Table S7). The closer faunal similarity observed between the TEP and Gal provinces (Forbes index = 0.89, Fig. 4B; Table S8), could be related to the geographical proximity between both provinces, and similarities in the oceanographic conditions prevailing in the region, contrasting with those present in the southernmost WTSP (see Sullivan Sealey & Bustamante, 1999).

In contrast to the EP provinces, those from the tropical WA are characterized by wider continental shelf and longer coastline with more geographic accidents, which results into higher habitat heterogeneity (Sullivan Sealey & Bustamante, 1999). The TNWA or “Wider Caribbean” (Sullivan Sealey & Bustamante, 1999; Fish, 2012; Lausche, 2008) is geographically complex, with large areas of shallow coastal shelf (Table S1), extensive coral reef development, extensive coastal line covered by mangroves, the largest number of continental and offshore islands, and a high marine diversity than clearly differentiate this region from the NBS and TSWA provinces (Sullivan Sealey & Bustamante, 1999; Carpenter, 2002; Miloslavich et al., 2011). The TNWA has the highest diversity and regional endemism (with at least 28 species) (Table S7) of shark and ray from Tropical America, and possibly from the all marine provinces of the Americas, being listed as hotspots of the shark and ray biodiversity in the world (Weigmann, 2016; Stein et al., 2018). Despite the great diversity at TNWA, there is still an uncertainty about exact number of chondrichthyan species in this province (Dulvy et al., 2014). The shark and ray faunal similarity between the TNWA and NBS (Forbes index = 0.96; Fig. 4B; Table S8) is probably related to abiotic conditions and geographical proximity between both provinces, and the effect caused by the “Amazon biogeographical barrier” (Rocha, 2003), which might represent a barrier for some taxa between these marine provinces and the southernmost TSWA. The biotic and abiotic conditions that characterize NBS are dominated by the large river discharges and plumes of the Amazon and Orinoco rivers (Sullivan Sealey & Bustamante, 1999). The plume produced by both rivers is characterized by turbid and low-salinity waters that reduce connectivity of northern and southern populations of many coastal organisms (Rocha, 2003; Luiz et al., 2012). These conditions could also affect the shark and ray diversity of the NBS. For example, the relation diversity vs. bathymetry of the NBS, contrasts with those of the other marine provinces of Tropical America, where NBS has a relative low diversity given its large percentage of bathymetry ranges between 0 and 200 m (Fig. 6E; Table S1).

### The fossil record of sharks and rays and factors affecting paleodiversity

The Miocene–Pleistocene fossil record of Tropical America includes 52 shark and 17 ray genera (Fig. 5A; Table 2). The Neogene basins of the WA have a higher diversity than those of the EP—this difference could result from poor sampling in the fossil record of the EP, especially in the early and middle Miocene intervals. The most diverse groups of sharks and rays from Tropical America throughout the Miocene–Pleistocene are the Carcharhiniformes and Myliobatiformes (Table 2). A similar pattern is observed in the extant diversity of the region (Fig. 2; Table 1), suggesting these groups have prevailed at least since the early Miocene.

The highest faunal similarity between the assemblages from the EP and WA is recorded during early Miocene time (Fig. 5C), but this result is compromised given the limited sampling available for the EP, consisting of five genera only. We think that this number indicates a lack of potential sedimentary exposures and limited sampling of existing ones and not a reflection of depauperate faunas at the time in that region. In the analysis, the assemblages of the middle Miocene of the WA and EP exhibit a lower similarity value. The decrease in faunal similarity between the EP and WA, during the early and middle Miocene is probably related to the early phases of closure of the CAS, which represented a barrier for marine biota (Lessios, 2008; Rocha et al., 2008; Leigh, O’Dea & Vermeij, 2014, O’Dea et al., 2016; and references therein). Although the CAS experienced a significant reduction of its width by the early Miocene (Farris et al., 2011) and most likely cessation of deep-water flow by the middle Miocene (Montes et al., 2015; Bacon et al., 2015; Jaramillo et al., 2017), shallow marine connections between the Caribbean and Pacific waters likely occurred until ca. 3 Ma ago (Coates & Stallard, 2013; Coates et al., 2004; Jaramillo et al., 2017). Our survey does not offer a direct biochronology and biostratigraphic inference to support in a precise way any of the postulated timings proposed for the closure of the CAS and origin the Panama Isthmus (Coates & Stallard, 2013; O’Dea et al., 2016; Jaramillo et al., 2017). However, the increasing shark and ray differentiation between EP and WA assemblages after the early/middle Miocene (Fig. 5C), support changes in sharks and rays faunistic composition during these geological intervals. After the interruption of flow between the EP and WA oceans, vicariant events took place, resulting in differential speciation or extinction, which could be reflected in the differences of diversity of the living sharks and rays in the region (Fig. 3). However, time-calibrated phylogenetic studies in some amphi-American shark genera such as lemon shark (*Negaprion*), angel sharks (*Squatina*), and hammer sharks (*Sphyrna*), suggest regional speciation with endemic species that diverged at least in the last 10 myr (Schultz et al., 2008; Stelbrink et al., 2010; Lim et al., 2010; Acero et al., 2016). The extinction rates estimated for shark genera are relatively low; however, it is important to consider that the boundary crossers method used can be affected by biases that result in underestimation of extinction rates (Alroy, 2010). More accurate methods exist (Alroy, 2008, 2014), but they are more demanding of the data and could not be used in our dataset. Nevertheless, our analysis revealed a higher extinction rate in Tropical America during the Pliocene (Fig. 5D), which corresponds with a global extinction event of marine megafauna (Pimiento et al., 2017).

Clues related with possible consequence of “post-CAS” environmental changes and other global processes can be observed. Our analysis revealed extirpation/extinction processes affecting at least 29 genera of sharks and rays in the Americas during Neogene times, especially during the final stage of this period. The sharks were the most affected, with at least 24 genera. The affected genera are mainly those associated to neritic/epipelagic species, although few associated with deep-water environments are also reported. Five genera of rays appear to be the least affected. Shark genera such as †*Carcharoides*, †*Cosmopolitodus*, †*Carcharocles*, †*Paradoxodon*, †*Paratodus*, †*Anotodus*, †*Pachyscyllium*, and †*Physogaleus*, and the ray †*Plinthicus*, had a wide distribution during the Neogene. They became extinct not only in the American continent, but also worldwide during different geological intervals. Many of them went extinct between the middle Miocene and Pliocene (Cappetta, 2012). However, some of these taxa, crossed the boundary between the late Neogene and the Quaternary, as is the case of †*Cosmopolitodus* and †*Hemipristis serra*, recently reported for the early Pleistocene of North America (Ebersole, Ebersole & Cicimurri, 2017). Shark genera such as †*Kruckowlamna*, *Dalatias*, *Pristiophorus, Carcharias*, *Isogomphodon*, *Trigonognathus*, *Scymnodon*, *Chiloscyllium*, *Nebrius*, *Iago*, *Chaenogaleus*, *Hemipristis*, and *Paragaleus* and the rays *Rhynchobatus*, *Taeniura*, *Taeniurops*, and *Aetomylaeus*, were possibly affected directly by the “post-CAS” environmental changes. Some of the above mentioned genera were regionally extirpated from the EP (e.g., *Dalatias*, *Pristiophoru*s, *Carcharias*, and *Isogomphodon*) or WA (e.g., *Deania, Heterodontus*, *Taeniurops*, and *Aetomylaeus*), but with living representatives in one of both sides. The above mentioned fossil record of *Dalatias* and *Isogomphodon* in the EP, have been reported exclusively from the Bahía Inglesa Formation (Late Miocene–Pliocene), Atacama Region, Northern Chile (Suárez, 2015; Villafaña & Rivadeneira, 2018). Other shark genera such as *Trigonognathus*, *Scymnodon*, *Chiloscyllium*, *Nebrius*, *Iago*, *Chaenogaleus*, *Hemipristis*, *Paragaleus*, and the family Stegostomatidae reported by Suárez (2015, fig. 4) and the rays *Rhynchobatus* and *Taeniura* became extinct in the Americas, but living representatives of them still exist in other marine regions of the world (Compagno, Dando & Fowler, 2005; Last, Seret & Naylor, 2016). Extinction and diversification processes in marine areas of EP and WA have been strongly linked to the contrasting environmental diversity between these regions (Lessios, 2008; Leigh, O’Dea & Vermeij, 2014). However, the habitat loss associated to sea-level oscillations during Pliocene–Pleistocene (Miller et al., 2005; De Boer et al., 2010), have also been shown to be associated with marine extinctions during this time, with a consequent erosion of functional diversity of the marine megafauna (Pimiento et al., 2017). The extirpation of the shark genus *Carcharias* from the EP has been shown to be related with a drop of global temperatures during the Pleistocene and the lowering of sea level that reduced the area of distribution of the taxon to the northern areas of EP resulting on the disappearance of suitable habitat (Cione et al., 2007). In contrast, the extirpation of the kitefin shark *Dalatias* and the sawshark *Pristiophorus* from EP at the end of the Neogene is intriguing. *Pristiophorus* had a wider distribution in the American continent during Miocene and Pliocene times (Carrillo-Briceño et al., 2016a); however, only one extant species (*Pristiophorus schroederi*) with a reduced distribution, inhabits the northern most part of the TNWA province, living over continental and insular slopes between 400 and 1,000 m (Kiraly, Moore & Jasinski, 2003). In the reference to *Dalatias*, the extant species prefers deep-water environments near the continental slope, although also it has been referred in shallower waters (Carrillo-Briceño et al., 2016a, fig. 6). The Neogene fossil record of *Dalatias* and *Pristiophorus* in the Americas indicates that these taxa have been inhabitants of both shallow and deep-water environments (Carrillo-Briceño et al., 2013, 2015b, 2016a, and references therein), which could have allowed a high vagility to overcome aquatic barriers associated to sea level and habitat loss. The extirpation of *Dalatias*, *Pristiophorus*, as well other marine fauna from the EP (Rivadeneira & Marquet, 2007; Villafaña & Rivadeneira, 2014), could be correlated with the expansion of upwelling areas and the cooling of surface waters during Pliocene–Pleistocene (Ibaraki, 1997; Ravelo et al., 2004; Lawrence, Liu & Herbert, 2006; Dowsett et al., 2013). Villafaña & Rivadeneira (2018), suggested that the extirpation in many shark and ray species from the temperate Pacific coast of South America during the Miocene–Recent interval, was the result of a combination of their tolerance to oceanographic conditions (e.g., salinity and thermal range) and life-history traits (e.g., body size).

In the WA, extirpation processes also affected taxa associated exclusively to deep-water environments. The best example is *Trigonognathus*, a shark with a fossil record that includes the Late Miocene of Panama and late Miocene–Pliocene of Venezuela (Aguilera & Rodrigues De Aguilera, 2001; Carrillo-Briceño et al., 2015b). The extant *Trigonognathus kabeyai* is a typical deep-water benthopelagic species inhabiting the upper continental slope at depths ranging between 330 and 360 m in Taiwanese and Hawaiian waters (Compagno, Dando & Fowler, 2005; Froese & Pauly, 2017). The extirpation of *Trigonognathus* from the WA does not seem to be associated with loss of habitat due sea level changes, because the region has abundant areas with deep environments (Sullivan Sealey & Bustamante, 1999) that sustain another great variety of sharks and rays. The shark and ray Pleistocene faunas in Tropical America are little known (Tables S3 and S4), with only few faunal references from Ecuador (Carrillo-Briceño, Aguilera & Rodríguez, 2014), the Caribbean (Aguilera, 2010), and Panama (Table S3). The Pleistocene record of sharks and rays in Tropical America, is critical to infer if there is any relation between extinction/extirpation process in this group and the habitat loss produced by sea-level oscillations, or other oceanographic biological, ecological, and oceanographic alterations (O’Dea et al., 2007; Lessios, 2008; Leigh, O’Dea & Vermeij, 2014; Pimiento et al., 2017).

## Conclusion

The study revealed: (1) a marked contrast in shark and ray species diversity between EP and WA faunas; the WA being more diverse (105 shark and 77 ray species) than EP (80 shark and 60 rays species). There is a higher percentage of shark species (49% for EP and 37% for WA) than ray species (17% for EP and 13% for WA) shared between the two regions. (2) The faunal similarity among the respective provinces of the EP and the WA suggests that geographic, biotic, and abiotic conditions, as well natural barriers (e.g., off shore island isolation and large volumes of river discharge) have a direct consequence in shark and ray diversity and geographic distribution. In addition, there is a strong relationship between shark and ray diversity with area and coastal length of each province. (3) TNWA province is the most diverse in total number and endemic species, suggesting this region as the hotspot of sharks and rays in Tropical America, and possibly in the continent. (4) Miocene–Pleistocene faunal compilation for Tropical America resulted in a total paleodiversity of 52 shark and 17 ray genera for Miocene–Pleistocene. The Neogene basins of the WA have higher diversity than EP, although this is likely related to a sampling bias. (5) There is high faunal similarity between the assemblages from the EP and WA recorded during early Miocene, followed by a decrease in faunal similarity in the younger time intervals. (6) There is a higher extinction rate of shark genera in Tropical America for the Pliocene, corresponding to a global extinction event of marine megafauna. (7) There is a gap of knowledge of fossil sharks and rays in the Pleistocene, which is a critical time interval as it could shed new light about the direct effects of habitat loss produced by sea-level oscillations, and other oceanographic alterations that occurred at the end of the Neogene and during the Pleistocene.

## Supplemental Information

10.7717/peerj.5313/supp-1 Supplemental Information 1 Marine provinces of Tropical America and their geographic indicators. Total area, coastal line, and the area of bathymetry are taken from Sullivan Sealey & Bustamante, 1999.

10.7717/peerj.5313/supp-2 Supplemental Information 2 Extant shark and ray diversity of Tropical America by marine provinces. Presence “1,” absence “0.” Data based on FishBase website (Froese & Pauly, 2017), Ocean Biogeographic Information System (OBIS: http://www.iobis.org/), and literature referred in Data S1. Abbreviations: Tropical Eastern Pacific (TEP), Galapagos (Gal), Warm Temperate Southeastern Pacific (WTSP), Tropical Northwestern Atlantic (TNWA), North Brazil Shelf (NBS), and Tropical Southwestern Atlantic (TSWA).

10.7717/peerj.5313/supp-3 Supplemental Information 3 The fossil record of sharks and rays (genera) from Tropical America, tabulated by country. The compilation was based on new and published data. For published data see references in Data S2, for new data in Table S6. Abbreviations: early (E), middle (M), late (L), Miocene (Mi), Pliocene (P), and Pleistocene (Pl). Extinct taxa are referred by †. New records referred in Table S6 (*).

10.7717/peerj.5313/supp-4 Supplemental Information 4 The shark and ray paleodiversity (genera) of Tropical America. Presence “1,” absence “0”. The (*) is referred for genera that are inferred to exist but have no fossil record in a determined interval. Data based on Table S3. Abbreviations: Eastern Central Pacific (EP), Western Central Atlantic (WA), early Miocene (Emi), middle Miocene (MMi), late Miocene (LMi), Pliocene (P), Pleistocene (Pl), and Recent (Rect). Extinct genera are referred by †.

10.7717/peerj.5313/supp-5 Supplemental Information 5 Revised and taxonomically expanded shark and ray assemblages from Tropical America. Collection: OA & JDCB, taxonomic determinations: JDCB. Paleontological collection: Natural History Museum of Basel (NMB S.A.), Switzerland; Palaeontological Institute and Museum at the University of Zurich Switzerland (PIMUZ); Paleontological collection of the Alcaldía Bolivariana de Urumaco, Venezuela (AMU-CURS). Abbreviations: tooth (T), dermal denticles (Dt), vertebra (Vb).

10.7717/peerj.5313/supp-6 Supplemental Information 6 New shark and ray assemblages from Tropical America. Collection: OA & JDCB, taxonomic determinations: JDCB. Paleontological collection: Museo Angel Segundo Lopez, Tara-Tara, Venezuela (MTT-V); Mapuka Museum of Universidad del Norte, Barranquilla, Colombia (MUN-STRI), Natural History Museum of Basel (NMB S.A.), Switzerland; Palaeontological Institute and Museum at the University of Zurich, Switzerland (PIMUZ); René Kindlimann (RK) private collection with public access, Uster, Switzerland; Paleontological collection of the Alcaldía Bolivariana de Urumaco, Venezuela (AMU-CURS). Abbreviations: tooth (T), dermal denticles (Dt), vertebra (Vb).

10.7717/peerj.5313/supp-7 Supplemental Information 7 Geographic range and regional endemic shark and ray species from the marine provinces of Tropical America. Data based on FishBase website (Froese & Pauly, 2017), Ocean Biogeographic Information System (OBIS: http://www.iobis.org/), and Table S2. Abbreviations: Eastern Atlantic (EA), Eastern Pacific (EP), Galapagos (Gal), Northwestern Atlantic (TNWA), North Brazil Shelf (NBS), Temperate Northern Atlantic (TNA), Temperate Northern Pacific (TNP), Tropical Eastern Pacific (TEP), Tropical Southwestern Atlantic (TSWA), Warm Temperate Southwestern Atlantic (WTSA), Warm Temperate Southeastern Pacific (WTSP), Western Atlantic (WA), and Western Pacific (WP).

10.7717/peerj.5313/supp-8 Supplemental Information 8 Similarity coefficient values using the modified Forbes distance (Alroy, 2015).

10.7717/peerj.5313/supp-9 Supplemental Information 9 Similarity coefficient values using the modified Forbes distance (Alroy, 2015) for the fossil assemblages.

10.7717/peerj.5313/supp-10 Supplemental Information 10 References list of extant shark and rays from Tropical America used in Table S2.

10.7717/peerj.5313/supp-11 Supplemental Information 11 References list of fossil shark and rays from Tropical America used in Table S3.

## Additional Information and Declarations

### Data Availability

The following information was supplied regarding data availability: The raw data are provided in the Supplemental Files.
