# Supplementary material for: Shark and ray diversity in the Tropical America (Neotropics)—an examination of environmental and historical factors affecting diversity
Source: PeerJ. 2018 Jul 20;6:e5313. doi: 10.7717/peerj.5313 (PMC6055692; doi:10.7717/peerj.5313)
Supplement: Supplemental Information 1 — Total area, coastal line, and the area of bathymetry are taken from Sullivan Sealey & Bustamante, 1999. [file peerj-06-5313-s001.pdf]

# Summarized information of the marine provinces of Tropical America

| | Eastern Pacific | Total area of the Ecoregion (Km2) | Total coastline length (Km) | Total area of Bathymetry (% within Province) | | |
|---|---|---|---|---|---|---|
| | | | | 0-200 m | 200-1000 m | > 1000 m |
| Province | Tropical East Pacific | 3596024 | 13264 | 5.83 | 2.72 | 91.45 |
| | Galapagos | 877041 | 1426 | 1 | 3 | 96 |
| | Warm Temperate Southeastern Pacific | 996559 | 3472 | 10 | 5 | 85 |

| | Western Atlantic | Total area of the Ecoregion (Km2) | Total coastline length (Km) | Total area of Bathymetry (% within Province) | | |
|---|---|---|---|---|---|---|
| | | | | 0-200 m | 200-1000 m | > 1000 m |
| Province | Tropical Northwestern Atlantic | 5378994 | 43556 | 17.34 | 10.66 | 72 |
| | North Brazil Shelf | 940628 | 12066 | 46 | 6 | 48 |
| | Tropical Southwestern Atlantic | 2443887 | 4176 | 7 | 2 | 91 |

# Detailed geographic indicators of the marine provinces of Tropical America

| Eastern Pacific | | Area of the Ecoregion (Km2) | % total area of the province | Coastline length (Km) | % total Coastline of the province | Area of Bathymetry (Km2) and % within Ecoregion | | | | | |
|---|---|---|---|---|---|---|---|---|---|---|---|
| | | | | | | 0-200 m | % | 200-1000 m | % | > 1000 m | % |
| Province | Tropical East Pacific | | | | | | | | | | |
| Ecoregion | -Clipperton and Revillagigedos | 1035466 | 29 | 118 | 1 | 314 | < 1 | 502 | < 1 | 1034650 | 100 |
| | -Mexican Tropical Pacific | 767409 | 18 | 1412 | 11 | 12144 | 2 | 16831 | 3 | 738433 | 95 |
| | -Chiapas-Nicarauga | 392204 | 12 | 2638 | 20 | 84893 | 22 | 29256 | 7 | 278055 | 71 |
| | -Nicoya | 330336 | 10 | 2756 | 21 | 26242 | 8 | 12842 | 4 | 291252 | 88 |
| | -Coco Islands | 298829 | 9 | 26 | < 1 | 43 | < 1 | 2487 | 1 | 296299 | 99 |
| | -Panama Bight | 508357 | 15 | 4227 | 32 | 54996 | 11 | 27150 | 5 | 426211 | 84 |
| | -Guayaquil | 263423 | 8 | 2087 | 16 | 31036 | 12 | 8441 | 3 | 223947 | 85 |
| | Total | 3596024 | 100 | 13264 | 100 | 209668 | | 97509 | | 3288847 | |
| Province | Galapagos | | | | | | | | | | |
| Ecoregion | -Northern Galápagos Islands | 224673 | 26 | 15 | 1 | 30 | < 1 | 698 | < 1 | 223945 | 100 |
| | -Eastern Galápagos Islands | 411657 | 47 | 1001 | 70 | 7157 | 2 | 24910 | 6 | 379590 | 92 |
| | -Western Galápagos Islands | 240711 | 27 | 410 | 29 | 1932 | 1 | 1388 | 1 | 237391 | 98 |
| | Total | 877041 | 100 | 1426 | 100 | 9119 | | 26996 | | 840926 | |
| Province | Warm Temperate Southeastern Pacific | | | | | | | | | | |
| Ecoregion | -Central Peru * | 328220 | 19 | 1164 | 19 | 65686 | 20 | 20242 | 6 | 242292 | 74 |
| | -Humboldtian * | 668339 | 39 | 2308 | 37 | 33249 | 5 | 30587 | 5 | 604503 | 90 |
| | -Central Chile | *out analysis* | *out analysis* | *out analysis* | *out analysis* | *out analysis* | | *out analysis* | | *out analysis* | |
| | -Araucanian | *out analysis* | *out analysis* | *out analysis* | *out analysis* | *out analysis* | | *out analysis* | | *out analysis* | |
| | Total | 996559 | 58 | 3472 | 56 | 98935 | | 50829 | | 846795 | |

| | Western Atlantic | Area of the Ecoregion (Km2) | % total area of the province | Coastline length (Km) | % total Coastline of the province | Area of Bathymetry (Km2) and % within Ecoregion | | | | | |
|---|---|---|---|---|---|---|---|---|---|---|---|
| | | | | | | 0-200 m | % | 200-1000 m | % | > 1000 m | % |
| Province | Tropical Northwestern Atlantic | | | | | | | | | | |
| Ecoregion | -Bermuda | *out analysis* | *out analysis* | *out analysis* | *out analysis* | *out analysis* | | *out analysis* | | *out analysis* | |
| | -Bahamian | 855017 | 15 | 7225 | 16.58 | 123274 | 15 | 102236 | 12 | 629508 | 73 |
| | -Caribbean | | | | | | | | | | |
| | Eastern Caribbean<br>Greater Antilles<br>Southern Caribbean<br>Southwestern Caribbean<br>Western Caribbean | 3310037 | 57 | 29477 | 67.67 | 451057 | 13 | 347665 | 11 | 2511315 | 76 |
| | -Floridian | 27195 | 0.5 | 1238 | 2.84 | 22073 | 78 | 5123 | 22 | 0 | 0 |
| | -Southern Gulf of Mexico | 1186745 | 20 | 5616 | 12.83 | 336407 | 29 | 118733 | 10 | 731603 | 61 |
| | Total | 5378994 | 92.5 | 43556 | 99.2 | 932811 | | 573757 | | 3872426 | |
| Province | North Brazil Shelf | | | | | | | | | | |
| Ecoregion | -Guianan | 384566 | 41 | 1814 | 15 | 147820 | 38 | 28936 | 8 | 207809 | 54 |
| | -Amazonian | 556062 | 59 | 10252 | 85 | 287516 | 52 | 23678 | 4 | 244869 | 44 |
| | Total | 940628 | 100 | 12066 | 100 | 435336 | | 52614 | | 452678 | |
| Province | Tropical Southwestern Atlantic | | | | | | | | | | |
| Ecoregion | -São Pedro and São Paulo Islands | 465415 | 19 | 12 | 0.3 | 7 | < 1 | 23 | < 1 | 465361 | 100 |
| | -Fernando de Naronha and Atoll das Rocas<br>-Northeastern Brazil | 1043712 | 43 | 2106 | 50.4 | 74082 | 7 | 26531 | 3 | 943100 | 90 |
| | -Eastern Brazil | 497583 | 20.2 | 2050 | 49 | 99667 | 20 | 21678 | 4 | 376238 | 76 |
| | -Trindade and Martin Vaz Islands | 437177 | 17.8 | 8 | 0.3 | 21 | < 1 | 65 | < 1 | 437114 | 100 |
| | Total | 2443887 | 100 | 4176 | 100 | 173777 | | 48297 | | 2221813 | |

* Ecoregions used to define the Warm Temperate Southeastern Pacific province in Tropical America
