# Supplementary material for: Shark and ray diversity in the Tropical America (Neotropics)—an examination of environmental and historical factors affecting diversity
Source: PeerJ. 2018 Jul 20;6:e5313. doi: 10.7717/peerj.5313 (PMC6055692; doi:10.7717/peerj.5313)
Supplement: Supplemental Information 2 — Presence “1,” absence “0.” Data based on FishBase website (Froese & Pauly, 2017), Ocean Biogeographic Information System (OBIS: http://www.iobis.org/), and literature referred in Data S1. Abbreviations: Tropical Eastern Pacific (TEP), Galapagos (Gal), Warm Temperate Southeastern Pacific (WTSP), Tropical Northwestern Atlantic (TNWA), North Brazil Shelf (NBS), and Tropical Southwestern Atlantic (TSWA). [file peerj-06-5313-s002.pdf]

				Provinces						Habitat			Habit			
				Eastern Central Pacific			Western Central Atlantic									
Order	Family	Genus	Species	TEP	Gal	WTSP	TNWA	NBS	TSWA	Benthic/Demersal	Benthopelagic	Pelagic	Coastal	Oceanic	Bathyal	Depth range m
Hexanchiformes	Chlamydoselachidae	*Chlamydoselachus*	*C. anguineus*	0	0	1	0	1	0	0	1	0	0	0	1	0 - 1570
	Heptranchidae	*Heptranchias*	*H. perlo*	0	0	1	1	1	1	0	1	0	0	0	1	0 - 1000
	Hexanchidae	*Hexanchus*	*H. griseus*	1	0	1	1	0	1	0	1	0	1	0	1	1-1250
			H. vitulus	0	0	0	1	0	0	0	1	0	1	0	1	90 - 600
		Notorynchus	*N. cepedianus*	1	0	1	0	0	1	0	1	0	1	0	0	0 - 570
Echinorhiniformes	Echinorhinidae	*Echinorhinus*	*E. cookei*	1	1	1	0	0	0	0	1	0	1	0	1	11 - 1100
Squaliformes	Squalidae	*Cirrhigaleus*	*C. asper*	0	0	0	1	0	1	0	1	0	0	0	1	200 - 650
		Squalus	*S. acanthias*	0	0	1	1	1	1	0	1	0	1	0	1	0 - 1460
			S. albicaudus	0	0	0	0	0	1	0	1	0	0	0	1	195 - 421
			S. bahiensis	0	0	0	0	0	1	0	1	0	0	0	1	? - 599
			S. cubensis	0	0	0	1	0	0	0	1	0	1	0	1	60 - 400
			S. lobularis	0	0	0	0	0	1	0	1	0	1	0	1	?
			S. mitsukurii	0	0	1	1	0	0	0	1	0	1	0	1	29 - 600
	Centrophoridae	*Centrophorus*	*C. granulosus*	0	0	0	1	1	1	0	1	0	1	0	1	50 - 1440
			C. squamosus	0	1	0	0	0	0	0	1	0	0	0	1	145 - 2400
			C. tessellatus	0	0	0	1	0	0	0	1	0	1	0	1	20 - 732
		Deania	*D. calcea*	0	0	1	0	0	0	0	1	0	1	0	1	60 - 1490
	Etmopteridae	*Aculeola*	*A. nigra*	1	0	1	0	0	0	0	1	0	1	0	1	110 - 560
		Centroscyllium	*C. nigrum*	1	1	1	0	0	0	0	1	0	0	0	1	269 - 1170
		Etmopterus	*E. bigelowi*	0	0	1	1	1	1	0	1	0	0	0	1	163 - 1000
			E. benchleyi	1	0	0	0	0	0	0	1	0	0	0	1	836 - 1443
			E. bullisi	0	0	0	1	0	0	0	1	0	0	0	1	? - 850
			E. carteri	0	0	0	1	0	0	0	1	0	0	0	1	283 - 356
			E. gracilispinis	0	0	0	1	1	1	0	1	0	1	0	1	70 - 1000
			E. hillianus	0	0	0	1	0	0	0	1	0	0	0	1	180 - 717
			E. litvinovi	0	0	1	0	0	0	0	1	0	0	0	1	? - 1050
			E. perryi	0	0	0	1	0	0	0	1	0	0	0	1	283 - 439
			E. pusillus	0	0	1	1	0	1	0	1	0	0	0	1	0 - 1070
			E. robinsi	0	0	0	1	0	0	0	1	0	0	0	1	400 - 800
			E. schultzi	0	0	0	1	1	0	0	1	0	0	0	1	200 - 1000
			E. virens	0	0	0	1	1	0	0	1	0	1	0	1	100 - 1000
	Somniosidae	*Centroscymnus*	*C. coelolepis*	0	0	0	1	1	1	0	1	0	0	0	1	150 - 3700
			C. owstonii	0	0	1	0	0	0	0	1	0	0	0	1	150-1459
		Centroselachus	*C. crepidater*	0	0	1	0	0	0	0	1	0	0	0	1	200-1500
		Somniosus	*S. antarcticus*	0	0	1	0	0	0	0	1	0	1	0	1	0-1440
			S. pacificus	1	0	0	0	0	0	0	1	0	1	0	1	0 - 2000
		Zameus	*Z. squamulosus*	0	0	1	1	1	1	0	1	0	1	0	1	0 - 2200
	Oxynotidae	*Oxynotus*	*O. caribbaeus*	0	0	0	1	0	0	0	1	0	0	0	1	402 - 457
	Dalatiidae	*Dalatias*	*D. licha*	0	0	0	1	0	1	0	1	0	1	0	1	37 - 1800
		Euprotomicrus	*E. bispinatus*	0	0	1	0	0	1	0	0	1	0	1	0	0 - 1800
		Isistius	*I. brasiliensis*	1	1	0	1	0	1	0	0	1	0	1	0	0 - 3700

			I. plutodus	0	0	0	1	0	1	0	0	1	0	1	0	60 - 200
		Squaliolus	*S. laticaudus*	0	0	0	1	1	1	0	0	1	0	1	0	200 - 1200
Pristiophoriformes	Pristiophoridae	*Pristiophorus*	*P. schroederi*	0	0	0	1	0	0	0	1	0	1	0	1	400 - 1000
Squatiniformes	Squatinidae	*Squatina*	*S. armata*	1	0	1	0	0	0	1	0	0	1	0	0	?
			S. californica	1	0	0	0	0	0	1	0	0	1	0	0	3 - 205
			S. david	0	0	0	1	0	0	1	0	0	1	0	0	144
			S. dumeril	0	0	0	1	0	0	1	0	0	1	0	1	1 - 1375
Heterodontiformes	Heterodontidae	*Heterodontus*	*H. francisci*	1	0	0	0	0	0	1	0	0	1	0	0	2 - 152
			H. mexicanus	1	0	0	0	0	0	1	0	0	1	0	0	0 - 50
			H. quoyi	1	1	1	0	0	0	1	0	0	1	0	0	3 - 40
Orectolobiformes	Ginglymostomatidae	*Ginglymostoma*	*G. cirratum*	0	0	0	1	1	1	1	0	0	1	0	0	0 - 130
			G. unami	1	0	0	0	0	0	1	0	0	1	0	0	0 - 130?
	Rhincodontidae	*Rhincodon*	*R. typus*	1	1	1	1	1	1	0	0	1	0	1	0	0 - 700
Lamniformes	Mitsukurinidae	*Mitsukurina*	*M. owstoni*	0	0	0	1	1	1	0	1	0	1	0	1	30 - 1300
	Odontaspidae	*Carcharias*	*C. taurus*	0	0	0	1	0	1	0	1	0	1	0	0	1 - 191
		Odontaspis	*O. ferox*	1	1	1	1	0	1	0	1	0	1	0	1	10 - 2000
			O. noronhai	0	0	0	0	0	1	0	1	0	1	0	1	60 - 1000
	Pseudocarcharhiidae	*Pseudocarcharias*	*P. kamoharai*	1	0	1	1	1	1	0	0	1	0	1	0	0 - 590
	Lamnidae	*Carcharodon*	*C. carcharias*	1	1	1	1	1	1	0	1	0	1	1	0	0 - 1280
		Isurus	*I. oxyrinchus*	1	1	1	1	1	1	0	0	1	1	1	0	0 - 740
			I. paucus	0	0	1	1	0	1	0	0	1	1	1	0	30 - 760
		Lamna	*L.. nasus*	0	0	1	0	0	0	0	0	1	1	1	0	0 - 715
	Alopiidae	*Alopias*	*A. pelagicus*	1	1	1	0	0	0	0	0	1	1	1	0	0 - 700
			A. superciliosus	1	1	1	1	1	1	0	0	1	1	1	0	0 - 730
			A. vulpinus	1	0	1	1	1	1	0	0	1	1	1	0	0 - 550
	Cetorhinidae	*Cetorhinus*	*C. maximus*	1	1	1	1	0	1	0	0	1	1	1	0	0 - 2000
	Megachasmidae	*Megachasma*	*M. pelagios*	1	0	1	1	0	1	0	0	1	0	1	0	5 - 600
Carcharhiniformes	Pentanchidae	*Apristurus*	*A. brunneus*	1	0	1	0	0	0	1	0	0	1	0	1	33 - 1306
			A. canutus	0	0	0	1	0	0	1	0	0	0	0	1	500 - 1000
			A. kampae	1	1	1	0	0	0	1	0	0	0	0	1	? - 1888
			A. laurussonii	0	0	0	1	0	0	1	0	0	0	0	1	560 - 1550
			A. nasutus	1	0	1	0	0	0	1	0	0	0	0	1	400 - 925
			A. parvipinnis	0	0	0	1	1	0	1	0	0	0	0	1	600 - 1380
			A. riveri	0	0	0	1	0	0	1	0	0	0	0	1	700 - 1500
			A. stenseni	1	1	0	0	0	0	1	0	0	0	0	1	915 - 975
		Bythaelurus	*B. canescens*	0	0	1	0	0	0	1	0	0	1	0	1	250 - 700
			B. giddingsi	0	1	0	0	0	0	1	0	0	1	0	1	428 - 562
		Cephaloscyllium	*C. ventriosum*	1	0	1	0	0	0	1	0	0	1	0	1	5 - 457
		Cephalurus	*C. cephalus*	1	0	0	0	0	0	1	0	0	1	0	1	155 - 927
		Galeus	*G. antillensis*	0	0	0	1	0	0	1	0	0	0	0	1	150 - 700
			G. arae	0	0	0	1	0	0	1	0	0	1	0	1	250 - 750
			G. cadenati	0	0	0	1	0	0	1	0	0	0	0	1	439- 700
			G. springeri	0	0	0	1	0	0	1	0	0	1	0	1	450 - 700
		Parmaturus	*P. campechiensis*	0	0	0	1	0	0	1	0	0	1	0	1	? - 1097
	Scyliorhinidae	*Schroederichthys*	*S. chilensis*	0	0	1	0	0	0	1	0	0	1	0	1	?
			S. maculatus	0	0	0	1	0	0	1	0	0	1	0	1	190-410

		S. saurisqualus	0	0	0	0	0	1	1	0	0	1	0	1	122-425
		S. tenuis	0	0	0	0	1	0	1	0	0	1	0	1	72-450
	Scyliorhinus	*S. boa*	0	0	0	1	1	0	1	0	0	1	0	1	229-676
		S. cabofriensis	0	0	0	0	0	1	1	0	0	1	0	1	?
		S. haeckelii	0	0	0	1	1	1	1	0	0	1	0	1	37-439
		S. hesperius	0	0	0	1	0	0	1	0	0	1	0	1	234-457
		S. meadi	0	0	0	1	0	0	1	0	0	0	0	1	329-548
		S. retifer	0	0	0	1	0	0	1	0	0	1	0	1	73-754
		S. torrei	0	0	0	1	0	0	1	0	0	1	0	1	229-650
		S. ugoi	0	0	0	0	0	1	1	0	0	0	0	1	400 - 500
Proscylliidae	*Eridacnis*	*E. barbouri*	0	0	0	1	0	0	1	0	0	0	0	1	430-613
Pseudotriakidae	*Pseudotriakis*	*P. microdon*	0	0	0	1	0	1	1	0	0	0	0	1	173 - 1890
Triakidae	*Galeorhinus*	*G. galeus*	1	0	1	0	0	1	0	1	0	1	1	0	120 - 185
	Mustelus	*M. albipinnis*	1	1	0	0	0	0	0	1	0	1	0	0	30 - 281
		M. canis	0	0	0	1	1	1	0	1	0	1	0	1	0 - 800
		M. dorsalis	1	0	0	0	0	0	0	1	0	1	0	0	20 - 200
		M. henlei	1	0	1	0	0	0	0	1	0	1	0	1	0 - 281
		M. higmani	0	0	0	1	1	1	0	1	0	1	0	1	1 - 900
		M. lunulatus	1	0	0	0	0	0	0	1	0	1	0	0	10 - 94
		M. mento	0	1	1	0	0	0	0	1	0	1	0	0	16 -50
		M. minicanis	0	0	0	1	0	0	0	1	0	1	0	0	71-183
		M. norrisi	0	0	0	1	1	1	0	1	0	1	0	0	1 - 100
		M. sinusmexicanus	0	0	0	1	0	0	0	1	0	1	0	1	20 - 250
		M. whitneyi	1	0	1	0	0	0	0	1	0	1	0	1	16 - 211
	Triakis	*T. acutipinna*	1	0	0	0	0	0	0	1	0	1	0	0	?
		T. maculata	0	1	1	0	0	0	0	1	0	1	0	0	?
Carcharhinidae	*Carcharhinus*	*C. acronotus*	0	0	0	1	1	1	0	1	0	1	0	0	18 -64
		C. albimarginatus	1	1	0	0	0	0	0	1	0	1	1	0	0 - 800
		C. altimus	1	1	0	1	1	1	0	1	0	1	1	0	0 - 430
		C. brachyurus	1	0	1	1	0	1	0	1	0	1	1	0	0 - 360
		C. brevipinna	0	0	0	1	1	1	0	1	0	1	0	0	0 - 100
		C. falciformis	1	1	1	1	1	1	0	0	1	1	1	0	0 - 4000
		C. galapagensis	1	1	0	1	0	1	0	1	0	1	1	0	1 - 286
		C. isodon	0	0	0	1	1	0	0	1	0	1	0	0	? - 10
		C. leucas	1	0	0	1	1	1	0	1	0	1	0	0	1 - 152
		C. limbatus	1	1	0	1	1	1	0	1	0	1	0	0	0 - 100
		C. longimanus	1	1	1	1	1	1	0	1	0	0	1	0	0 - 230
		C. obscurus	1	0	0	1	1	1	0	1	0	1	0	0	0 - 400
		C. perezii	0	0	0	1	1	1	0	1	0	1	0	0	1 - 65
		C. plumbeus	0	1	0	1	1	1	0	1	0	1	0	0	0 - 500
		C. porosus	1	0	1	1	1	1	0	1	0	1	0	0	16 - 32
		C. signatus	0	0	0	1	1	1	0	1	0	1	1	0	50 - 100
	Galeocerdo	*G. cuvier*	1	1	0	1	1	1	0	1	0	1	1	0	0 - 800
	Isogomphodon	*I. oxyrhynchus*	0	0	0	0	1	1	0	1	0	1	0	0	4 - 40
	Nasolamia	*N. velox*	1	1	0	0	0	0	0	1	0	1	0	0	15 - 192
	Negaprion	*N. brevirostris*	1	0	0	1	1	1	0	1	0	1	0	0	0 - 92
	Prionace	*P. glauca*	1	1	1	1	1	1	0	0	1	0	1	0	0 - 350
	Rhizoprionodon	*R. lalandii*	0	0	0	1	1	1	0	1	0	1	0	0	3 - 70
		R. longurio	1	0	0	0	0	0	0	1	0	1	0	0	? - 100
		R. porosus	0	0	0	1	1	1	0	1	0	1	0	1	? - 500
		R. terraenovae	0	0	0	1	1	0	0	1	0	1	0	1	0 - 280

		Triaenodon	*T. obesus*	1	1	0	0	0	0	1	0	0	1	0	0	1 - 330
	Sphyrnidae	*Sphyrna*	*S. corona*	1	0	0	0	0	0	0	1	0	1	0	0	?
			S. lewini	1	1	0	1	1	1	0	1	0	1	1	0	0 - 1000
			S. media	1	0	0	1	1	1	0	1	0	1	0	0	?
			S. mokarran	1	1	0	1	1	1	0	1	0	1	1	0	1 - 300
			S. tiburo	1	1	0	1	1	1	0	1	0	1	0	0	10 - 80
			S. tudes	0	0	0	1	1	1	0	1	0	1	0	0	? - 12
			S. zygaena	1	1	1	1	0	1	0	1	0	1	1	0	0 - 200
Rhinopristiformes	Pristidae	*Pristis*	*P. pectinata*	1	0	0	1	1	1	1	0	0	1	0	0	0- 10
			P. pristis	1	0	1	1	1	1	1	0	0	1	0	0	1 - ?
	Rhinobatidae	*Pseudobatos*	*P. glaucostigma*	1	0	0	0	0	0	1	0	0	1	0	0	1 - 112
			P. horkelii	0	0	0	0	0	1	1	0	0	1	0	0	1 - 100
			P. lentiginosus	0	0	0	1	0	0	1	0	0	1	0	0	0 - 30
			P. leucorhynchus	1	0	0	0	0	0	1	0	0	1	0	0	2 - 8
			P. percellens	0	0	0	1	1	1	1	0	0	1	0	0	0 - 110
			P. prahli	1	0	0	0	0	0	1	0	0	1	0	0	? - 70
			P. planiceps	1	1	1	0	0	0	1	0	0	1	0	0	?
	Trygonorrhinidae	*Zapteryx*	*Z. exasperata*	1	1	1	0	0	0	1	0	0	1	0	0	1 - 200
			Z. xyster	1	1	1	0	0	0	1	0	0	1	0	0	?
Rajiformes	Arhynchobatidae	*Bathyraja*	*B. aguja*	1	0	1	0	0	0	1	0	0	0	0	1	605 - 981
			B. longicauda	0	0	1	0	0	0	1	0	0	0	0	1	580-735
			B. peruana	1	0	1	0	0	0	1	0	0	0	0	1	605-1207
			B. spinosissima	1	1	0	0	0	0	1	0	0	0	0	1	800 - 2938
		Notoraja	*N. martinezi*	1	0	0	0	0	0	1	0	0	0	0	1	1256–1472
		Rioraja	*R. agassizii*	0	0	0	0	0	1	1	0	0	1	0	0	? - 130
		Sympterygia	*S. brevicaudata*	1	0	1	0	0	0	1	0	0	1	0	0	? - 18
	Gurgesiellidae	*Cruriraja*	*C. atlantis*	0	0	0	1	0	0	1	0	0	0	0	1	512 - 778
			C. cadenati	0	0	0	1	0	0	1	0	0	0	0	1	? - 458
			C. poeyi	0	0	0	1	0	0	1	0	0	0	0	1	385 - 870
			C. rugosa	0	0	0	1	0	0	1	0	0	0	0	1	366 - 1007
		Fenestraja	*F. atripinna*	0	0	0	1	0	0	1	0	0	0	0	1	? - 595
			F. ishiyamai	0	0	0	1	0	0	1	0	0	0	0	1	400 - 1000
			F. plutonia	0	0	0	1	1	0	1	0	0	0	0	1	290 - 750
			F. sinusmexicanus	0	0	0	1	0	0	1	0	0	0	0	1	? - 311
		Gurgesiella	*G. atlantica*	0	0	0	1	1	1	1	0	0	0	0	1	374 - 480
			G. furvescens	1	1	1	0	0	0	1	0	0	0	0	1	?
	Rajidae	*Amblyraja*	*A. frerichsi*	0	0	1	0	0	0	1	0	0	0	0	1	720-2609
			A. hyperborea	1	0	0	0	0	0	1	0	0	1	0	1	140 - 2500
		Breviraja	*B. colesi*	0	0	0	1	0	0	1	0	0	0	0	1	366 - 522
			B. mouldi	0	0	0	1	0	0	1	0	0	0	0	1	? - 503
			B. nigriventralis	0	0	0	1	1	0	1	0	0	0	0	1	549 - 776
			B. spinosa	0	0	0	1	1	0	1	0	0	0	0	1	323 - 675
		Dactylobatus	*D. armatus*	0	0	0	1	0	0	1	0	0	0	0	1	300 - 900
			D. clarkii	0	0	0	1	1	1	1	0	0	0	0	1	475 - 1000
		Dipturus	*D. bullisi*	0	0	0	1	1	0	1	0	0	0	0	1	200 - 600
			D. ecuadoriensis	1	0	0	0	0	0	1	0	0	0	0	1	?
			D. garricki	0	0	0	1	0	0	1	0	0	0	0	1	275 - 476
			D. olseni	0	0	0	1	0	0	1	0	0	1	0	1	91 - 238
			D. oregoni	0	0	0	1	0	0	1	0	0	0	0	1	369 - 468

			D. teevani	0	0	0	1	1	1	1	0	0	0	0	1	320 - 940
		Leucoraja	*L. garmani*	0	0	0	1	0	0	1	0	0	1	0	1	55 - 530
			L. lentiginosa	0	0	0	1	0	0	1	0	0	1	0	1	53 - 457
			L. yucatanensis	0	0	0	1	0	0	1	0	0	1	0	1	? - 423
		Malacoraja	*M. obscura*	0	0	0	0	0	1	1	0	0	0	0	1	808 - 1105
		Rostroraja	*R. ackleyi*	0	0	0	1	0	0	1	0	0	1	0	1	32 - 384
			R. bahamensis	0	0	0	1	0	0	1	0	0	0	0	1	388 - 412
			R. cervigoni	0	0	0	1	0	0	1	0	0	1	0	1	30 - 180
			R. eglanteria	0	0	0	1	0	0	1	0	0	1	0	1	0 - 330
			R. equatorialis	1	0	1	0	0	0	1	0	0	1	0	0	20 - 200
			R. texana	0	0	0	1	0	0	1	0	0	1	0	0	1 - 183
			R. velezi	1	1	1	0	0	0	1	0	0	1	0	0	35 - 300
		Rajella	*R. bigelowi*	0	0	0	1	0	0	1	0	0	0	0	1	650 - 4156
			R. eisenhardti	0	1	0	0	0	0	1	0	0	0	0	1	757 - 907
			R. fuliginea	0	0	0	1	1	0	1	0	0	0	0	1	732 - 824
			R. nigerrima	1	0	1	0	0	0	1	0	0	0	0	1	780 - 960
			R. purpuriventralis	0	0	0	1	1	1	1	0	0	0	0	1	? - 922
			R. sadowskii	0	0	1	0	0	1	1	0	0	0	0	1	1067 - 1353
	Anacanthobatidae	*Schroederobatis*	*S. americana*	0	0	0	1	1	0	1	0	0	0	0	1	200 - 900
		Springeria	*S. folirostris*	0	0	0	1	0	0	1	0	0	0	0	1	425 - 472
			S. longirostris	0	0	0	1	0	0	1	0	0	0	0	1	530 - 1052
Torpediformes	Narcinidae	*Benthobatis*	*B. marcida*	0	0	0	1	0	0	1	0	0	0	0	1	200 - 923
		Diplobatis	*D. picta*	0	0	0	1	1	1	1	0	0	1	0	0	? - 91
			D. colombiensis	0	0	0	1	0	0	1	0	0	1	0	0	? - 99
			D. guamachensis	0	0	0	1	0	0	1	0	0	1	0	0	?
			D. ommata	1	0	0	0	0	0	1	0	0	1	0	0	3 - 94
		Discopyge	*D. tschudii*	1	0	1	0	0	1	1	0	0	1	0	0	5 - 165
		Narcine	*N. bancroftii*	0	0	0	1	1	0	1	0	0	1	0	0	?
			N. brasiliensis	0	0	0	1	1	1	1	0	0	1	0	0	1 - 43
			N. entemedor	1	1	0	0	0	0	1	0	0	1	0	0	1 - 100
			N. leoparda	1	0	0	0	0	0	1	0	0	1	0	0	0 - 17
			N. vermiculata	1	0	0	0	0	0	1	0	0	1	0	0	10 - 60
	Torpedinidae	*Tetronarce*	*T. nobiliana*	0	0	0	1	1	1	1	0	0	1	0	1	2 - 800
			T. tremens	1	1	1	0	0	0	1	0	0	1	0	1	1 - 168
		Torpedo	*T. andersoni*	0	0	0	1	0	0	1	0	0	1	0	0	? - 229
Myliobatiformes	Dasyatidae	*Bathytoshia*	*B. centroura*	0	0	0	1	1	1	1	0	0	1	0	1	3 - 270
		Fontitrygon	*F. colarensis*	0	0	0	1	1	0	0	1	0	1	0	0	6 - ?
			F. geijskesi	0	0	0	1	1	1	1	0	0	1	0	0	5 - 25
		Hypanus	*H. americanus*	0	0	0	1	1	1	1	0	0	1	0	0	0 - 53
			H. dipterurus	1	1	1	0	0	0	1	0	0	1	0	0	1 - 70
			H. guttatus	0	0	0	1	1	1	1	0	0	1	0	0	1 - 36
			H. longus	1	1	0	0	0	0	1	0	0	1	0	0	?
			H. marianae	0	0	0	0	1	1	1	0	0	1	0	0	2 - 15
			H. sabinus	0	0	0	1	0	0	1	0	0	1	0	0	1 - 25
			H. say	0	0	0	1	1	1	1	0	0	1	0	0	1 - 10
		Pteroplatytrygon	*P. violacea*	1	1	1	1	1	1	0	1	0	1	0	1	1 - 381
		Taeniurops	*T. meyeni*	0	1	0	0	0	0	1	0	0	1	0	1	1 - 500
	Gymnuridae	*Gymnura*	*G. altavela*	0	0	0	1	0	1	1	0	0	1	0	0	5 - 100
			G. crebripunctata	1	0	1	0	0	0	1	0	0	1	0	0	?

		G. marmorata	1	0	1	0	0	0	1	0	0	1	0	0	1 - 94
		G. micrura	0	0	0	1	1	0	1	0	0	1	0	0	?
Potamotrygonidae	*Styracura*	*S. pacifica*	1	1	0	0	0	0	1	0	0	1	0	0	?
		S. schmardae	0	0	0	1	1	1	1	0	0	1	0	0	?
Urotrygonidae	*Urobatis*	*U. halleri*	1	0	0	0	0	0	1	0	0	1	0	0	0 - 91
		U. concentricus	1	0	0	0	0	0	1	0	0	1	0	0	1 - 109
		U. jamaicensis	0	0	0	1	0	0	1	0	0	1	0	0	1 - 25
		U. pardalis	1	0	0	0	0	0	1	0	0	1	0	0	1 - 14
		U. tumbesensis	1	0	0	0	0	0	1	0	0	1	0	0	?
	Urotrygon	*U. aspidura*	1	0	1	0	0	0	1	0	0	1	0	0	5 - 100
		U. munda	1	0	0	0	0	0	1	0	0	1	0	0	4 - 51
		U. rogersi	1	0	0	0	0	0	1	0	0	1	0	0	2 - 30
		U. chilensis	1	1	1	0	0	0	1	0	0	1	0	0	1 - 60
		U. cimar	1	0	0	0	0	0	1	0	0	1	0	0	0 - 85
		U. microphthalmum	0	0	0	1	1	0	1	0	0	1	0	0	?
		U. nana	1	0	0	0	0	0	1	0	0	1	0	0	?
		U. reticulata	1	0	0	0	0	0	1	0	0	1	0	0	?
		U. simulatrix	1	0	0	0	0	0	1	0	0	1	0	0	?
		U. venezuelae	0	0	0	1	0	0	1	0	0	1	0	0	?
Aetobatidae	*Aetobatus*	*A. narinari*	1	1	0	1	1	1	0	1	0	1	1	0	1 - 80
Myliobatidae	*Aetomylaeus*	*A. asperrimus*	1	1	0	0	0	0	0	1	0	1	0	0	? - 50
	Myliobatis	*M. californica*	1	1	0	0	0	0	0	1	0	1	0	0	0 - 108
		M. chilensis	0	0	1	0	0	0	0	1	0	1	0	0	?
		M. freminvillei	0	0	0	1	1	1	0	1	0	1	0	0	0 - 100
		M. goodei	0	0	0	1	1	1	0	1	0	1	0	0	1 - 130
		M. longirostris	1	0	0	0	0	0	0	1	0	1	0	0	? - 50
		M. peruvianus	0	1	1	0	0	0	0	1	0	1	0	0	1 - 100
		M. ridens	0	0	0	0	0	1	0	1	0	1	0	0	5 -47
Rhinopteridae	*Rhinoptera*	*R. bonasus*	0	0	0	1	1	1	0	1	0	1	0	0	0 - 22
		R. brasiliensis	0	0	0	0	0	1	0	1	0	1	0	0	0 - 20
		R. steindachneri	1	1	1	0	0	0	0	1	0	1	1	0	0 - 30
Mobulidae	*Mobula*	*M. birostris*	1	1	1	1	1	1	0	1	0	1	1	0	0 - 120
		M. mobular	0	0	0	1	0	0	0	1	0	1	1	0	?
		M. tarapacana	1	1	1	1	1	1	0	1	0	1	1	0	0 - 30
		M. hypostoma	1	0	0	1	1	1	0	1	0	1	1	0	?
		M. thurstoni	1	0	0	0	0	1	0	1	0	1	1	0	0 - 100
		M. munkiana	1	1	0	0	0	0	0	1	0	1	1	0	0 - 15
