# Supplementary material for: Shark and ray diversity in the Tropical America (Neotropics)—an examination of environmental and historical factors affecting diversity
Source: PeerJ. 2018 Jul 20;6:e5313. doi: 10.7717/peerj.5313 (PMC6055692; doi:10.7717/peerj.5313)
Supplement: Supplemental Information 3 — The compilation was based on new and published data. For published data see references in Data S2, for new data in Table S6. Abbreviations: early (E), middle (M), late (L), Miocene (Mi), Pliocene (P), and Pleistocene (Pl). Extinct taxa are referred by †. New records referred in Table S6 (*). [file peerj-06-5313-s003.pdf]

Family	Genus	Eastern central Pacific					Western Central Pacific																References
		Chile (Norte Grande)	Costa Rica	Ecuador	Panama	Peru	Barbados	Bonaire	Brazil	Colombia	Costa Rica	Cuba	Haiti	Jamaica	Martinique	Panama	Puerto Rico	Dominican Republic	Trinidad	The Grenadines	Venezuela	Southern Mexico	
**Sharks**																							
Chlamydoselachidae	*Chlamydoselachus*			MMi-LMi																			1
Hexanchidae	*Hexanchus*					P					LMi-EP												2–4
	Notorynchus																				LMI-EP		5–6
Heptranchidae	*Heptranchias*									EMi	EMi?-EP					LMi					EMi		4–5, 7–10
Squalidae	*Squalus*										LMi-EP					LMi					LMi-EP		4–8, 10
Centrophoridae	*Centrophorus*			MMi-EP	LMi					EMi						LMi					EMi		1, 4, 7–8, 10, 33
	Deania						EMi							MMi?							LMi-EP		5, 12–13
Etmopteridae	*Etmopterus*						EMi-MMi														LMi-EP		5–6, 10, 13
	Trigonognathus															LMi					LMi-EP		5, 8
Somniosidae	*Scymnodon*													MMi?									11
Dalatiidae	*Dalatias*						EMi			EMi						LMi					LMi-EP		5–8, 10
	Isistius			LMi-EP							LMi-EP					LMi					LMi-EP		1, 4–6, 8, 10, 14
	Squaliolus						EMi-MMi							MMi?									11, 13
Echinorhinidae	*Echinorhinus*					P																	2
Squatinidae	*Squatina*			LMi-EP		LMi-EP					LMi-EP					LMi					LMi-EP		1–2, 4–6, 8, 10, 15
Pristiophoridae	*Pristiophorus*	MMi-LMi		LMi-EP		MMi-P				EMi						LMi					LMi-EP		1–3, 5–8, 16–17
Heterodontidae	*Heterodontus*	MMi-LMi				MMi-P					LMi-EP					LMi					LMi-EP		2–6, 8, 10, 16–17
Hemiscyllidae	cf. *Chiloscyllium*								EMi														18
Ginglymostomatidae	*Ginglymostoma*			MMi-EP	LMi-P*	LMi					LMi-EP					LMi							1, 4, 19–20
	Nebrius								EMi	EMi*											EMi		7, 18, 21–23
Rhincodontidae	*Rhincodon*										LMi-EP												4
Mitsukurinidae	*Mitsukurina*										EMi?												9
Odontaspidae	*Carcharias*			LMi-EP		LMi-P					EMi?	EMi-MMi				LMi				MMi			1–3, 5–6, 8–10, 15–16, 24–26
	Odontaspis						EMi-MMi												EMi-MMi				5, 10, 12–13, 27
Pseudocarchariidae	*Pseudocarcharias*					LMi			EMi							LMi					LMi-EP		5, 8, 10, 15, 18, 22
Lamnidae	*Carcharodon*				Pl*	MMi-P										LMi, Pl*					LMi-EP		1–3, 5–6, 8, 10, 15, 28–31
	†*Carcharoides*					LMi																	15
	†*Cosmopolitodus*					LMi	EMi-MMi					EMi-MMi											12, 15, 24–25, 29, 32
	Isurus	MMi-LMi			LMi	EMi-LMi	EMi-MMi	Mi		EMi	EMi?,LMi									MMi	EMi		2–3, 7, 9, 12, 15–17, 24–26, 29, 33–34
†Otodontidae	†*Carcharocles*	P		LMi-EP	LMi	EMi-P		Mi	Mi		EMi-EP	EMi-EP	P	LMi-P	EMi	EMi-LMi	MMi	Mi	MMi-LMi	MMi	EMi-LP	EMi	1–4, 6–10, 12–13, 15–29, 31–42
	†*Megalolamna*					EMi				EMi													7, 43
	†*Paratodus*									EMi*						EP*			EMi		EMi		23, 27
Alopiidae	*Alopias*				LMi		EMi-MMi			EMi	LMi-EP					LMi							1, 4–8, 10, 12, 23, 25, 33
	†*Anotodus*					LMi				EMi*	LMi-EP					LMi*					LMi*		4, 15, 25
Pentanchidae	*Cephaloscyllium*					LMi*																	
Scyliorhinidae	†*Pachyscyllium*															LMi							8
	Scyliorhinus										LMi-EP												4
Triakidae	*Galeorhinus*	MMi-LMi				MMi-P										LMi					EMi		1–2, 6, 8, 10, 17, 25
	cf. *Iago*				LMi																		33
	Triakis					LMi*																	
	Mustelus				LMi	LMi*					LMi-EP					LMi					LMi-P		1, 4–6, 8, 10–11, 23
Hemigaleidae	*Chaenogaleus*										LMi-EP												4
	Hemipristis			MMi-EP	LMi	EMi-LMi	EMi-MMi		EMi	EMi	Mi?,LMi-EI	EMi-MMi				EMi,LMi			MMi-LMi		EMi-Pl*	Mi	1, 4–8, 10, 12–13, 18–23, 25, 28–29, 32–33, 35–37, 42, 44
	Paragaleus			LMi, Pl																	LMi		1, 36
Carcharhinidae	*Galeocerdo*			LMi-EP	LMi, Pl*	MMi-P			EMi		LMi-EP					EMi,LMi-P*			MMi		EMi-Pl*		1–2, 4–8, 10, 15, 18, 20–23, 25, 27, 29, 33, 35–36,
	†*Physogaleus*				LMi	LMi				EMi*	LMi-EP	EMi-MMi				EMi-LMi					EMi-LMi		4, 8, 15, 20, 23, 32–33, 35,
	Rhizoprionodon		MMi	MMi-Pl	LMi, P-Pl*				EMi		LMi-EP					LMi-Pl*					EMi-Pl*		1, 4–6, 8, 10, 18, 20, 22, 33, 36, 45
	Carcharhinus		MMi	EMi-Pl	LMi, P-Pl*	EMi-P	EMi-MMi		EMi	EMi-P*	MMi-Pl*	EMi-MMi		MMi-Pl		EMi-Pl*			EMi-LMi	Mi	EMi-Pl*	Mi	1-8, 10, 15, 19–27, 29, 31–36, 44–46
	Isogomphodon									EMi*	LMi-EP										EMi		4, 23
	Prionace					P															P*		2
	Negaprion		MMi	EMi-Pl	LMi, Pl*	LMi-P			EMi		EMi	EMi				LMi					EMi-Pl*		1, 5–6, 8, 10, 15, 18–25, 32-34, 36, 44–45
	†*Kruckowlamna*										LMi-EP												4
Sphyrnidae	*Sphyrna*			LMi-EP	LMi-P*	LMi-P	EMi-MMi		EMi	EMi*	MMi-Pl*	EMi				LMi-P*			MMi		EMi-Pl*		1–2, 4–8, 10, 12–13, 15, 18–25, 32–33, 36
**Rays**																							
Pristidae	*Pristis*				LMi-P*	LMi			EMi	EMi*	LMi-EP					EMi,LMi					EMi-P		4–5, 10, 15, 20, 23, 36
Rhinidae	*Rhynchobatus*				LMi-P*				EMi	EMi*	LMi-EP					LMi					EMi-P		4–6, 10, 18, 20, 33, 36
Rhinobatidae	*Pseudobatos*					LMi*															Pl*		
Rajidae	*Raja*										LMi-EP										LMi-EP		4–5
Narcinidae	*Narcine*																				Pl		10
Dasyatidae	*Dasyatis*		MMi		LMi-P*	MMi-LMi*			EMi	EMi*	LMi-EP										EMi-Pl*		2, 5–6, 10, 18–19, 23–25, 33, 36, 45
	cf. *Pteroplatytrygon*																				EMi		23
	Taeniura								EMi							LMi							18, 20
	cf. *Taeniurops*																				EMi		23
Potamotrygonidae	*Styracura*				LMi*				EMi														18
Urotrygonidae	*Urobatis*				LMi											EMi,LMi							20, 33, 35
Aetobatidae	*Aetobatus*		MMi	LMi-P	LMi	LMi				EMi*	LMi	EMi-MMI				LMi					EMi-P		1,5–6, 23, 18–19, 24–25, 32–33, 40, 45, 45
Myliobatidae	*Myliobatis*		MMi	EPL		LMi-P					LMi	EMi				LMi					MMi-P		2–3, 5–6, 8, 10, 19–20, 24–25, 32, 40, 42, 45
	Aetomylaeus								EMi	EMi*		EMi-LMi									EMi-P		18, 23, 47
Rhinopteridae	*Rhinoptera*		MMi	LMi-P	LMi				EMi	EMi*	LMi-EP					LMi					EMi-Pl*		1, 4–6, 10, 18, 20, 23–25, 33, 36, 42, 45
Mobulidae	*Mobula*			MMi-P	LMi-P*					EMi	LMi-EP					LMi					EMi		1, 5, 7, 20, 23, 33
	†*Plinthicus*									EMi*						EMi					EMi		23, 35
