# Supplementary material for: Shark and ray diversity in the Tropical America (Neotropics)—an examination of environmental and historical factors affecting diversity
Source: PeerJ. 2018 Jul 20;6:e5313. doi: 10.7717/peerj.5313 (PMC6055692; doi:10.7717/peerj.5313)
Supplement: Supplemental Information 4 — The (*) is referred for genera that are inferred to exist but have no fossil record in a determined interval. Data based on Table S3. Abbreviations: Eastern Central Pacific (EP), Western Central Atlantic (WA), early Miocene (Emi), middle Miocene (MMi), late Miocene (LMi), Pliocene (P), Pleistocene (Pl), and Recent (Rect). Extinct genera are referred by †. [file peerj-06-5313-s004.pdf]

| Order | Family | Genus | Eastern Central Pacific | | | | | | Western Central Atlantic | | | | | |
|---|---|---|---|---|---|---|---|---|---|---|---|---|---|---|
| | | | EMi | MMi | LMi | P | Pl | Rect | EMi | MMi | LMi | P | Pl | Rect |
| Hexanchiformes | Chlamydoselachidae | *Chlamydoselachus* | 0 | 1 | 1 | 1* | 1* | 1 | 0 | 0 | 0 | 0 | 0 | 1 |
| | Heptranchidae | *Heptranchias* | 0 | 0 | 0 | 0 | 0 | 1 | 1 | 1* | 1 | 1 | 1* | 1 |
| | Hexanchidae | *Hexanchus* | 0 | 0 | 0 | 1 | 1* | 1 | 0 | 0 | 1 | 1 | 1* | 1 |
| | | *Notorynchus* | 0 | 0 | 0 | 0 | 0 | 1 | 0 | 0 | 1 | 1 | 1* | 1 |
| Echinorhiniformes | Echinorhinidae | *Echinorhinus* | 0 | 0 | 0 | 1 | 1* | 1 | 0 | 0 | 0 | 0 | 0 | 0 |
| Squaliformes | Squalidae | *Cirrhigaleus* | 0 | 0 | 0 | 0 | 0 | 0 | 0 | 0 | 0 | 0 | 0 | 1 |
| | | *Squalus* | 0 | 0 | 0 | 0 | 0 | 1 | 0 | 0 | 1 | 1 | 1* | 1 |
| | Centrophoridae | *Centrophorus* | 0 | 1 | 1 | 1 | 1* | 1 | 1 | 1 | 1 | 1* | 1* | 1 |
| | | *Deania* | 0 | 0 | 0 | 0 | 0 | 1 | 1 | 1* | 1 | 1 | 0 | 0 |
| | Etmopteridae | *Aculeola* | 0 | 0 | 0 | 0 | 0 | 1 | 0 | 0 | 0 | 0 | 0 | 0 |
| | | *Centroscyllium* | 0 | 0 | 0 | 0 | 0 | 1 | 0 | 0 | 0 | 0 | 0 | 0 |
| | | *Etmopterus* | 0 | 0 | 0 | 0 | 0 | 1 | 1 | 1 | 1 | 1 | 1* | 1 |
| | | *Trigonognathus* | 0 | 0 | 0 | 0 | 0 | 0 | 0 | 0 | 1 | 1 | 0 | 0 |
| | Somniosidae | *Centroscymnus* | 0 | 0 | 0 | 0 | 0 | 1 | 0 | 0 | 0 | 0 | 0 | 1 |
| | | *Scymnodon* | 0 | 0 | 0 | 0 | 0 | 0 | 0 | 1 | 0 | 0 | 0 | 0 |
| | | *Centroselachus* | 0 | 0 | 0 | 0 | 0 | 1 | 0 | 0 | 0 | 0 | 0 | 0 |
| | | *Somniosus* | 0 | 0 | 0 | 0 | 0 | 1 | 0 | 0 | 0 | 0 | 0 | 0 |
| | | *Zameus* | 0 | 0 | 0 | 0 | 0 | 1 | 0 | 0 | 0 | 0 | 0 | 1 |
| | Oxynotidae | *Oxynotus* | 0 | 0 | 0 | 0 | 0 | 0 | 0 | 0 | 0 | 0 | 0 | 1 |
| | Dalatiidae | *Dalatias* | 0 | 0 | 0 | 0 | 0 | 0 | 1 | 1* | 1 | 1 | 1* | 1 |
| | | *Euprotomicrus* | 0 | 0 | 0 | 0 | 0 | 1 | 0 | 0 | 0 | 0 | 0 | 1 |
| | | *Isistius* | 0 | 0 | 1 | 1 | 1* | 1 | 0 | 0 | 1 | 1 | 1* | 1 |
| | | *Squaliolus* | 0 | 0 | 0 | 0 | 0 | 0 | 1 | 1 | 1* | 1* | 1* | 1 |
| Pristiophoriformes | Pristiophoridae | *Pristiophorus* | 0 | 1 | 1 | 1 | 0 | 0 | 1 | 1* | 1 | 1 | 1* | 1 |
| Squatiniformes | Squatinidae | *Squatina* | 0 | 0 | 1 | 1 | 1* | 1 | 0 | 0 | 1 | 1 | 1* | 1 |
| Heterodontiformes | Heterodontidae | *Heterodontus* | 0 | 1 | 1 | 1 | 1* | 1 | 1 | 1 | 1 | 1 | 0 | 0 |
| | Hemiscyllidae | cf. *Chiloscyllium* | 0 | 0 | 0 | 0 | 0 | 0 | 1 | 0 | 0 | 0 | 0 | 0 |
| Orectolobiformes | Ginglymostomatidae | *Ginglymostoma* | 0 | 0 | 1 | 1* | 1* | 1 | 0 | 0 | 1 | 1 | 1* | 1 |
| | | *Nebrius* | 0 | 0 | 0 | 0 | 0 | 0 | 1 | 0 | 0 | 0 | 0 | 0 |
| | Rhincodontidae | *Rhincodon* | 0 | 0 | 0 | 0 | 0 | 1 | 0 | 0 | 1 | 1 | 1* | 1 |
| Lamniformes | Mitsukurinidae | *Mitsukurina* | 0 | 0 | 0 | 0 | 0 | 0 | 1 | 0 | 0 | 0 | 0 | 1 |
| | Odontaspidae | *Carcharias* | 0 | 0 | 1 | 1 | 0 | 0 | 1 | 1 | 1* | 1* | 1* | 1 |
| | | *Odontaspis* | 0 | 0 | 0 | 0 | 0 | 1 | 1 | 1 | 0 | 0 | 0 | 1 |
| | Pseudocarcharhiidae | *Pseudocarcharias* | 0 | 0 | 1 | 1* | 1* | 1 | 1 | 1* | 1 | 1 | 1* | 1 |
| | Lamnidae | *Carcharodon* | 0 | 1 | 1 | 1 | 1 | 1 | 0 | 0 | 1 | 1 | 1* | 1 |
| | | †*Carcharoides* | 0 | 0 | 1 | 0 | 0 | 0 | 0 | 0 | 0 | 0 | 0 | 0 |
| | | †*Cosmopolitodus* | 0 | 0 | 1 | 1 | 0 | 0 | 1 | 1 | 0 | 0 | 0 | 0 |
| | | *Isurus* | 0 | 1 | 1 | 1* | 1* | 1 | 1 | 1 | 1 | 1 | 1* | 1 |
| | | *Lamna* | 0 | 0 | 0 | 0 | 0 | 1 | 0 | 0 | 0 | 0 | 0 | 0 |
| | †Otodontidae | †*Carcharocles* | 1 | 1 | 1 | 1 | 0 | 0 | 1 | 1 | 1 | 1 | 0 | 0 |
| | | †*Megalolamna* | 1 | 0 | 0 | 0 | 0 | 0 | 1 | 0 | 0 | 0 | 0 | 0 |
| | | †*Paratodus* | 0 | 0 | 0 | 0 | 0 | 0 | 1 | 1* | 1* | 1 | 0 | 0 |
| | Alopiidae | *Alopias* | 0 | 0 | 1 | 1* | 1* | 1 | 1 | 1 | 1 | 1 | 1* | 1 |
| | | *†Anotodus* | 0 | 0 | 1 | 0 | 0 | 0 | 1 | 1* | 1 | 0 | 0 | 0 |
| | Cetorhinidae | *Cetorhinus* | 0 | 0 | 0 | 0 | 0 | 1 | 0 | 0 | 0 | 0 | 0 | 1 |
| | Megachasmidae | *Megachasma* | 0 | 0 | 0 | 0 | 0 | 1 | 0 | 0 | 0 | 0 | 0 | 1 |
| Carcharhiniformes | Pentanchidae | *Apristurus* | 0 | 0 | 0 | 0 | 0 | 1 | 0 | 0 | 0 | 0 | 0 | 1 |
| | | *Bythaelurus* | 0 | 0 | 0 | 0 | 0 | 1 | 0 | 0 | 0 | 0 | 0 | 0 |

| | | | | | | | | | | | | | | |
|---|---|---|---|---|---|---|---|---|---|---|---|---|---|---|
| | | *Cephaloscyllium* | 0 | 0 | 1 | 1* | 1* | 1 | 0 | 0 | 0 | 0 | 0 | 0 |
| | | *Cephalurus* | 0 | 0 | 0 | 0 | 0 | 1 | 0 | 0 | 0 | 0 | 0 | 0 |
| | | *Galeus* | 0 | 0 | 0 | 0 | 0 | 0 | 0 | 0 | 0 | 0 | 0 | 1 |
| | | *Parmaturus* | 0 | 0 | 0 | 0 | 0 | 0 | 0 | 0 | 0 | 0 | 0 | 1 |
| | Scyliorhinidae | †*Pachyscyllium* | 0 | 0 | 0 | 0 | 0 | 0 | 0 | 0 | 1 | 0 | 0 | 0 |
| | | *Schroederichthys* | 0 | 0 | 0 | 0 | 0 | 1 | 0 | 0 | 0 | 0 | 0 | 1 |
| | | *Scyliorhinus* | 0 | 0 | 0 | 0 | 0 | 0 | 0 | 0 | 1 | 1 | 1* | 1 |
| | Proscylliidae | *Eridacnis* | 0 | 0 | 0 | 0 | 0 | 0 | 0 | 0 | 0 | 0 | 0 | 1 |
| | Pseudotriakidae | *Pseudotriakis* | 0 | 0 | 0 | 0 | 0 | 0 | 0 | 0 | 0 | 0 | 0 | 1 |
| | Triakidae | *Galeorhinus* | 0 | 1 | 1 | 1 | 1* | 1 | 1 | 1* | 1* | 1 | 1* | 1 |
| | | *Triakis* | 0 | 0 | 1 | 1 | 1* | 1 | 0 | 0 | 0 | 0 | 0 | 0 |
| | | cf. *Iago* | 0 | 0 | 1 | 0 | 0 | 0 | 0 | 0 | 0 | 0 | 0 | 0 |
| | | *Mustelus* | 0 | 0 | 1 | 1 | 1* | 1 | 1 | 1 | 1 | 1 | 1 | 1 |
| | Hemigaleidae | *Chaenogaleus* | 0 | 0 | 0 | 0 | 0 | 0 | 0 | 0 | 1 | 1 | 0 | 0 |
| | | *Hemipristis* | 1 | 1 | 1 | 1 | 0 | 0 | 1 | 1 | 1 | 1 | 1 | 0 |
| | | *Paragaleus* | 0 | 0 | 1 | 1* | 1 | 0 | 0 | 0 | 1 | 0 | 0 | 0 |
| | Carcharhinidae | *Carcharhinus* | 1 | 1 | 1 | 1 | 1 | 1 | 1 | 1 | 1 | 1 | 1 | 1 |
| | | *Galeocerdo* | 0 | 1 | 1 | 1 | 1 | 1 | 1 | 1 | 1 | 1 | 1* | 1 |
| | | *Isogomphodon* | 0 | 0 | 0 | 0 | 0 | 0 | 1 | 1* | 1 | 1 | 1* | 1 |
| | | †*Kruckowlamna* | 0 | 0 | 0 | 0 | 0 | 0 | 0 | 0 | 1 | 1 | 0 | 0 |
| | | *Nasolamia* | 0 | 0 | 0 | 0 | 0 | 1 | 0 | 0 | 0 | 0 | 0 | 0 |
| | | *Negaprion* | 1 | 1 | 1 | 1 | 1 | 1 | 1 | 1 | 1 | 1 | 1* | 1 |
| | | *Prionace* | 0 | 0 | 0 | 1 | 1* | 1 | 0 | 0 | 0 | 1 | 1* | 1 |
| | | †*Physogaleus* | 0 | 0 | 1 | 0 | 0 | 0 | 1 | 1 | 0 | 0 | 0 | 0 |
| | | *Rhizoprionodon* | 0 | 1 | 1 | 1 | 1 | 1 | 1 | 1 | 1 | 1 | 1* | 1 |
| | | *Triaenodon* | 0 | 0 | 0 | 0 | 0 | 1 | 0 | 0 | 0 | 0 | 0 | 0 |
| | Sphyrnidae | *Sphyrna* | 0 | 1 | 1 | 1 | 1* | 1 | 1 | 1 | 1 | 1 | 1 | 1 |
| Rhinopristiformes | Pristidae | *Pristis* | 0 | 0 | 1 | 0 | 0 | 1 | 1 | 1 | 1 | 1 | 1* | 1 |
| | Rhinidae | *Rhynchobatus* | 0 | 0 | 1 | 0 | 0 | 0 | 1 | 1 | 1 | 1 | 0 | 0 |
| | Rhinobatidae | *Pseudobatos* | 0 | 0 | 1 | 1* | 1* | 1 | 0 | 0 | 0 | 0 | 1 | 1 |
| | Trygonorrhinidae | *Zapterix* | 0 | 0 | 0 | 0 | 0 | 1 | 0 | 0 | 0 | 0 | 0 | 0 |
| Rajiformes | Arhynchobatidae | *Bathyraja* | 0 | 0 | 0 | 0 | 0 | 1 | 0 | 0 | 0 | 0 | 0 | 0 |
| | | *Notoraja* | 0 | 0 | 0 | 0 | 0 | 1 | 0 | 0 | 0 | 0 | 0 | 0 |
| | | *Rioraja* | 0 | 0 | 0 | 0 | 0 | 0 | 0 | 0 | 0 | 0 | 0 | 1 |
| | | *Sympterygia* | 0 | 0 | 0 | 0 | 0 | 1 | 0 | 0 | 0 | 0 | 0 | 0 |
| | Gurgesiellidae | *Cruriraja* | 0 | 0 | 0 | 0 | 0 | 0 | 0 | 0 | 0 | 0 | 0 | 1 |
| | | *Fenestraja* | 0 | 0 | 0 | 0 | 0 | 0 | 0 | 0 | 0 | 0 | 0 | 1 |
| | | *Gurgesiella* | 0 | 0 | 0 | 0 | 0 | 1 | 0 | 0 | 0 | 0 | 0 | 1 |
| | Rajidae | *Amblyraja* | 0 | 0 | 0 | 0 | 0 | 1 | 0 | 0 | 0 | 0 | 0 | 0 |
| | | *Breviraja* | 0 | 0 | 0 | 0 | 0 | 0 | 0 | 0 | 0 | 0 | 0 | 1 |
| | | *Dactylobatus* | 0 | 0 | 0 | 0 | 0 | 0 | 0 | 0 | 0 | 0 | 0 | 1 |
| | | *Dipturus* | 0 | 0 | 0 | 0 | 0 | 1 | 0 | 0 | 0 | 0 | 0 | 1 |
| | | *Leucoraja* | 0 | 0 | 0 | 0 | 0 | 0 | 0 | 0 | 0 | 0 | 0 | 1 |
| | | *Malacoraja* | 0 | 0 | 0 | 0 | 0 | 0 | 0 | 0 | 0 | 0 | 0 | 1 |
| | | *Rostroraja* | 0 | 0 | 0 | 1 | 1* | 1 | 0 | 0 | 1 | 1 | 1* | 1 |
| | | *Rajella* | 0 | 0 | 0 | 0 | 0 | 1 | 0 | 0 | 0 | 0 | 0 | 1 |
| | Anacanthobatidae | *Schroederobatis* | 0 | 0 | 0 | 0 | 0 | 0 | 0 | 0 | 0 | 0 | 0 | 1 |
| | | *Springeria* | 0 | 0 | 0 | 0 | 0 | 0 | 0 | 0 | 0 | 0 | 0 | 1 |
| Torpediformes | Narcinidae | *Benthobatis* | 0 | 0 | 0 | 0 | 0 | 0 | 0 | 0 | 0 | 0 | 0 | 1 |
| | | *Diplobatis* | 0 | 0 | 0 | 0 | 0 | 1 | 0 | 0 | 0 | 0 | 0 | 1 |
| | | *Discopyge* | 0 | 0 | 0 | 0 | 0 | 1 | 0 | 0 | 0 | 0 | 0 | 1 |

| | | | | | | | | | | | | | | |
|---|---|---|---|---|---|---|---|---|---|---|---|---|---|---|
| | | *Narcine* | 0 | 0 | 0 | 0 | 0 | 1 | 0 | 0 | 0 | 0 | 1 | 1 |
| | Torpedinidae | *Tetronarce* | 0 | 0 | 0 | 0 | 0 | 1 | 0 | 0 | 0 | 0 | 0 | 1 |
| | | *Torpedo* | 0 | 0 | 0 | 0 | 0 | 0 | 0 | 0 | 0 | 0 | 0 | 1 |
| Myliobatiformes | Dasyatidae | *Dasyatis* | 0 | 1 | 1 | 1 | 1* | 1? | 1 | 1 | 1 | 1 | 1 | 1? |
| | | *Bathytoshia* | 0 | 0 | 0 | 0 | 0 | 0 | 0 | 0 | 0 | 0 | 0 | 1 |
| | | *Fontitrygon* | 0 | 0 | 0 | 0 | 0 | 0 | 0 | 0 | 0 | 0 | 0 | 1 |
| | | *Hypanus* | 0 | 0 | 0 | 0 | 0 | 1 | 0 | 0 | 0 | 0 | 0 | 1 |
| | | *Pteroplatytrygon* | 0 | 0 | 0 | 0 | 0 | 1 | 1 | 1* | 1* | 1* | 1* | 1 |
| | | *Taeniura* | 0 | 0 | 0 | 0 | 0 | 0 | 1 | 1* | 1 | 0 | 0 | 0 |
| | | *Taeniurops* | 0 | 0 | 0 | 0 | 0 | 1 | 1 | 0 | 0 | 0 | 0 | 0 |
| | Gymnuridae | *Gymnura* | 0 | 0 | 0 | 0 | 0 | 1 | 0 | 0 | 0 | 0 | 0 | 1 |
| | Potamotrygonidae | *Styracura* | 0 | 0 | 1 | 1* | 1* | 1 | 1 | 1* | 1* | 1* | 1* | 1 |
| | Urotrygonidae | *Urobatis* | 0 | 0 | 1 | 1* | 1* | 1 | 1 | 1* | 1 | 1* | 1* | 1 |
| | | *Urotrygon* | 0 | 0 | 0 | 0 | 0 | 1 | 0 | 0 | 0 | 0 | 0 | 1 |
| | Aetobatidae | *Aetobatus* | 0 | 0 | 1 | 1 | 1 | 1 | 1 | 1 | 1 | 1 | 1* | 1 |
| | Myliobatidae | *Aetomylaeus* | 0 | 0 | 0 | 0 | 0 | 1 | 1 | 1 | 1 | 1 | 0 | 0 |
| | | *Myliobatis* | 0 | 0 | 1 | 1 | 1 | 1 | 1 | 1 | 1 | 1 | 1* | 1 |
| | Rhinopteridae | *Rhinoptera* | 0 | 0 | 1 | 1 | 1* | 1 | 1 | 1 | 1 | 1 | 1 | 1 |
| | Mobulidae | *Mobula* | 0 | 1 | 1 | 1 | 1* | 1 | 1 | 1* | 1 | 1 | 1* | 1 |
| | | †*Plinthicus* | 0 | 0 | 0 | 0 | 0 | 0 | 1 | 0 | 0 | 0 | 0 | 0 |
