# Supplementary material for: Shark and ray diversity in the Tropical America (Neotropics)—an examination of environmental and historical factors affecting diversity
Source: PeerJ. 2018 Jul 20;6:e5313. doi: 10.7717/peerj.5313 (PMC6055692; doi:10.7717/peerj.5313)
Supplement: Supplemental Information 5 — Collection: OA & JDCB, taxonomic determinations: JDCB. Paleontological collection: Natural History Museum of Basel (NMB S.A.), Switzerland; Palaeontological Institute and Museum at the University of Zurich Switzerland (PIMUZ); Paleontological collection of the Alcaldía Bolivariana de Urumaco, Venezuela (AMU-CURS). Abbreviations: tooth (T), dermal denticles (Dt), vertebra (Vb). [file peerj-06-5313-s005.pdf]

Geological Unit	Age	Country	Region
Río Banano Formation	Late Miocene-Pliocene?	Costa Rica	Limón Region (Caribbean coast)

Taxon	N° Specimens	Catalog number	Other number	Locality	Collection
Carcharhiniformes					
Galeocerdo cuvier	1 (T)	NMB/to be assigend	PPP-3251	Santa Rita	1998

Geological Unit	Age	Country	Region
Angostura Formation	Middle-Late Miocene	Ecuador	Bordon Basin

Taxon	N° Specimens	Catalog number	Other number	Locality	Collection
Carcharhiniformes					
Carcharhinus sp.	1 (T)	NMB S.A.1540	PPP-3459	Río Santiago	1999
Carcharhinus sp.	1 (T)	NMB S.A.1541	PPP-3587	Falvio Alfaro	1999
Carcharhinus sp.	4 (T)	NMB S.A.1542a-d	PPP-3587	Falvio Alfaro	1999
Rhizoprionodon sp.	1 (T)	NMB S.A.1543	PPP-3465	Río Santiago	1999
†*Negaprion eurybathrodon*	1 (T)	NMB S.A.1544	PPP-3587	Falvio Alfaro	1999
Sphyrna sp.	1 (T)	NMB S.A.1545	PPP-3456	Río Santiago	1999

Geological Unit	Age	Country	Region
Jama Formation	Early Pleistocene	Ecuador	Manabi Basin

Taxon	N° Specimens	Catalog number	Other number	Locality	Collection
Carcharhiniformes					
Carcharhinus sp.	1 (T)	NMB S.A.1546	PPP-3563	Jama (Punta La Cereza)	1999

Geological Unit	Age	Country	Region
Onzole Formation	Late Miocene-Early Pliocene	Ecuador	Bordon Basin

Taxon	N° Specimens	Catalog number	Other number	Locality	Collection
Lamniformes					
Lamnidae indet.	1 (T)	NMB S.A.1547	PPP-3494	Río Camarones	1999

Geological Unit	Age	Country	Region
Chagres Formation	Late Miocene	Panama	Caribbean coast

Taxon	N° Specimens	Catalog number	Other number	Locality	Collection
Squaliformes					
Dalatias licha	4 (T)	PIMUZ-A/I-4647		Río Indio	2014
Squalus sp.	1 (T)	PIMUZ-A/I-4689		Río Indio	2014
Centrophorus sp.	1 (T)	PIMUZ-A/I-4692		Río Indio	2014
Pristiophoriformes					
Pristiophorus sp.	9 (T)	PIMUZ-A/I-4690		Río Indio	2014
Heterodontiformes					
Heterodontus sp.	1 (T)	NMB S.A.1551	PPP-3214	Río Indio	1998
Heterodontus sp.	1 (T)	NMB S.A.1552	PPP-3218	Río Indio	1998
Lamniformes					
†*Carcharocles megalodon*	1 (T)	NMB/to be assigend	PPP-3218	Río Indio	1998
Alopias superciliosus	1 (T)	PIMUZ-A/I-4696		Río Indio	2014
Carcharhiniformes					
†*Hemipristis serra*	1 (T)	NMB/to be assigend	PPP-3218	Río Indio	1998
Mustelus sp.	1 (T)	NMB S.A.1553	PPP-3214	Miguel de la Borda	1998
Mustelus sp.	1 (T)	NMB S.A.1554	PPP-3215	Miguel de la Borda	1998
Mustelus sp.	3 (T)	NMB S.A.1555	PPP-3226	Río Indio	1998
Galeorhinus galeus	1 (T)	PIMUZ-A/I-4693		Río Indio	2014
Carcharhinus brachyurus	2 (T)	PIMUZ-A/I-4684		Río Indio	2014
Carcharhinus leucas	2 (T)	PIMUZ-A/I-4683, 78		Río Indio, Punta Mansueto	2014
Carcharhinus falciformis	1 (T)	PIMUZ-A/I-4687		Río Indio	2014
Carcharhinus plumbeus	2 (T)	PIMUZ-A/I-4686		Río Indio	2014
Carcharhinus sp.	1 (T)	NMB/to be assigend	PPP-3218	Río Indio	1998
Negaprion sp.	1 (T)	PIMUZ-A/I-4688		Río Indio	2014
Sphyrna sp.	1 (T)	NMB S.A.1556	PPP-3226	Río Indio	1998
Carcharhinidae indet.	1 (T)	NMB S.A.1557	PPP-3221	Río Indio	1998
Myliobatiformes					
Myliobatis sp.	3 (T)	PIMUZ-A/I-4602		Río Indio	2014
Chondrichthyan indet.	1 (Dt)	NMB/to be assigend	PPP-3218	Río Indio	1998

Geological Unit	Age	Country	Region
Chucunaque Formation	Late Miocene	Panama	Darien (Pacific Coast)

Taxon	N° Specimens	Catalog number	Other number	Locality	Collection
Myliobatiformes					
Dasyatis sp.	3 (T)	NMB S.A.1562a/b/c	PPP-1578	Río Chico	1994

Geological Unit	Age	Country	Region
Gatún Formation	Late Miocene	Panama	Caribbean coast

Taxon	N° Specimens	Catalog number	Other number	Locality	Collection
Carcharhiniformes					
†*Hemipristis serra*	1 (T)	NMB/to be assigend	PPP-04	Sabanita (Colón)	1986
†*Hemipristis serra*	1 (T)	NMB/to be assigend	PPP-226	Isla Payardi	1987
†*Hemipristis serra*	2 (T)	NMB S.A.1563a/b	PPP-2164	Isla Payardi	1995
Paragaleus sp.	1 (T)	NMB S.A.1564	PPP-44	Río Chagres	1986
Carcharhinus albimarginatus	1 (T)	NMB S.A.1565	PPP-2164	Isla Payardi	1995
Carcharhinus obscurus	1 (T)	NMB/to be assigend	PPP-226	Isla Payardi	1987
Carcharhinus perezi	1 (T)	NMB S.A.1566	PPP-220	Sabanita (Colón)	1987
Carcharhinus perezi	3 (T)	NMB/to be assigend	PPP-2164	Isla Payardi	1995
Carcharhinus perezi	1 (T)	NMB/to be assigend	PPP-2164	Isla Payardi	1995
Carcharhinus cf. C. *plumbeus*	1 (T)	NMB/to be assigend	PPP-2164	Isla Payardi	1995
Carcharhinus sp.	1 (T)	NMB/to be assigend	PPP-01	Sabanita (Colón)	1986
Carcharhinus sp.	1 (T)	NMB/to be assigend	PPP-04	Sabanita (Colón)	1986
Carcharhinus sp.	1 (T)	NMB/to be assigend	PPP-10	Sabanita (Colón)	1986
Carcharhinus sp.	1 (T)	NMB/to be assigend	PPP-44	Río Chagres	1986
Carcharhinus sp.	1 (T)	NMB/to be assigend	PPP-220	Isla Payardi	1987
Carcharhinus sp.	1 (T)	NMB/to be assigend	PPP-2164	Isla Payardi	1995
Carcharhinus sp.	1 (T)	NMB/to be assigend	PPP-2164	Isla Payardi	1995
Carcharhinus sp.	3 (T)	NMB/to be assigend	PPP-2167	Village San Judas Tadeo	1995
Carcharhinus sp.	1 (T)	NMB/to be assigend	PPP-2168	Village San Judas Tadeo	1995
Galeocerdo cuvier	1 (T)	NMB/to be assigend	PPP-04	Sabanita (Colón)	1986
Galeocerdo cuvier	1 (T)	NMB S.A.1567	PPP-2164	Isla Payardi	1995
Galeocerdo cuvier	1 (T)	NMB S.A.1568	PPP-2164	Isla Payardi	1995
Rhizoprionodon sp.	1 (T)	NMB S.A.1569	PPP-44	Río Chagres	1986
†*Negaprion eurybathrodon*	1 (T)	NMB S.A.1570	PPP-04	Sabanita (Colón)	1986
†*Negaprion eurybathrodon*	1 (T)	NMB/to be assigend	PPP-2164	Isla Payardi	1995
†*Negaprion eurybathrodon*	1 (T)	NMB/to be assigend	PPP-2164	Isla Payardi	1995
Sphyrna zygaena	1 (T)	NMB/to be assigend	PPP-2164	Isla Payardi	1995
Sphyrna sp.	1 (T)	NMB/to be assigend	PPP-1	Sabanita (Colón)	1986
Sphyrna sp.	1 (T)	NMB/to be assigend	PPP-10	Sabanita (Colón)	1986
Sphyrna sp.	1 (T)	NMB/to be assigend	PPP-2168	Village San Judas Tadeo	1995
Rhinopristiformes				Village San Judas Tadeo	1995
Rhynchobatus sp.	1 (T)	NMB/to be assigend	PPP-2168	Village San Judas Tadeo	1995
Myliobatiformes					
cf. *Dasyatis*	1 (T)	NMB S.A.1571	PPP-2163	Isla Payardi	1995
Aetomylaeus	1 (T)	NMB/to be assigend	PPP-2164	Isla Payardi	1995
cf. *Aetomylaeus*	1 (T)	NMB S.A.1572	PPP-2164	Isla Payardi	1995
Rhinoptera sp.	5 (T)	NMB S.A.1573a-e	PPP-2164	Isla Payardi	1995
Rhinoptera sp.	1 (T)	NMB/to be assigend	PPP-04	Sabanita (Colón)	1986
Rhinoptera sp.	1 (T)	NMB/to be assigend	PPP-2164	Isla Payardi	1995
Rhinoptera sp.	1 (T)	NMB/to be assigend	PPP-2164	Isla Payardi	1995
Myliobatidae indet.	1 (T)	NMB/to be assigend	PPP-2167	Village San Judas Tadeo	1995
Mobula sp.	1 (T)	NMB S.A.1574	PPP-2163	Isla Payardi	1995
Mobula sp.	1 (T)	NMB S.A.1575	PPP-2163	Isla Payardi	1995
Mobula sp.	1 (T)	NMB S.A.1576	PPP-2163	Isla Payardi	1995
Mobula sp.	1 (T)	NMB S.A.1577	PPP-2167	Village San Judas Tadeo	1995

Chondrichthyan indet.	1 (Vb)	NMB/to be assigend	PPP-2164	Isla Payardi	1995

Geological Unit	Age	Country	Region
Urumaco Formation	Late Miocene	Venezuela	Western Venezuela

Taxon	N° Specimens	Catalog number	Other number	Locality	Collection
Carcharhiniformes					
Carcharhinus cf. *leucas*	1 (T)	AMU-CURS-776	—	Corralito (La Playita-Upper Mb.)	2001
Carcharhinus obscurus	1 (T)	AMU-CURS-777	—	Corralito (La Playita-Upper Mb.)	2001
Rhizoprionodon sp.	1 (T)	AMU-CURS-775	—	Río Urumaco (Middle Mb.)	1997?
Myliobatiformes					
Dasyatis sp.	1 (T)	AMU-CURS-774	—	Corralito (Upper Mb.)	1995
Aetomylaeus sp.	1 plate (7 T)	CIAAP-UNEFM-1380	—	Qda. El Mamón (Upper Mb.)	?
Rhinoptera sp.	3 (T)	AMU-CURS-774	—	Corralito Sur (Upper Mb.)	2011
