# Supplementary material for: Shark and ray diversity in the Tropical America (Neotropics)—an examination of environmental and historical factors affecting diversity
Source: PeerJ. 2018 Jul 20;6:e5313. doi: 10.7717/peerj.5313 (PMC6055692; doi:10.7717/peerj.5313)
Supplement: Supplemental Information 6 — Collection: OA & JDCB, taxonomic determinations: JDCB. Paleontological collection: Museo Angel Segundo Lopez, Tara-Tara, Venezuela (MTT-V); Mapuka Museum of Universidad del Norte, Barranquilla, Colombia (MUN-STRI), Natural History Museum of Basel (NMB S.A.), Switzerland; Palaeontological Institute and Museum at the University of Zurich, Switzerland (PIMUZ); René Kindlimann (RK) private collection with public access, Uster, Switzerland; Paleontological collection of the Alcaldía Bolivariana de Urumaco, Venezuela (AMU-CURS). Abbreviations: tooth (T), dermal denticles (Dt), vertebra (Vb). [file peerj-06-5313-s006.pdf]

Geological Unit	Age	Country	Region
Castilletes Formation	Early Miocene	Colombia	Guajira Peninsula (Caribbean coast)

Taxon	N° Specimens	Catalog number	Other number	Locality	Collection
Orectolobiformes					
Nebrius sp.	4 (T)	MUN-STRI-41136	—	390090	2013-14
Lamniformes					
†*Carcharocles* sp.	2 (T)	MUN-STRI-41145	—	290824	2013-14
Carcharhiniformes					
†*Hemipristis serra*	2 (T)	MUN-STRI-41137	—	390090	2013-14
†*Carcharhinus ackermanni*	2 (T)	MUN-STRI-37703		430202	2013-14
Galeocerdo sp.	2 (T)	MUN-STRI-37703	—	430202	2013-14
Carcharhinus spp.	2 (T)	MUN-STRI-41142	—	390090	2013-14
Negaprion sp.	26 (T)	MUN-STRI-41133	—	390090	2013-14
Isogomphodon sp.	2 (T)	MUN-STRI-41184	—	430053	2013-14
†*Physogaleus* sp.	2 (T)	MUN-STRI-41132	—	390090	2013-14
Sphyrna sp.	4 (T)	MUN-STRI-41143	—	390090	2013-14
Rhinopristiformes					
Pristis sp.	1 (T)	MUN-STRI-37395	—	390094	2013-14
Rhynchobatus sp.	4 (T)	MUN-STRI-42132	—	390094	2013-14
Myliobatiformes					
Dasyatis sp.	2 (T)	MUN-STRI-42135	—	390094	2013-14
Aetobatus sp.	1 (T)	MUN-STRI-16551	—	390085	2013-14
Aetomylaeus sp.	1 (T)	MUN-STRI-16708	—	390090	2013-14
Rhinoptera sp.	121 (T)	MUN-STRI-41139	—	390090	2013-14
†*Plinthicus* sp.	1 (T)	MUN-STRI-41185	—	430053	2013-14

Geological Unit	Age	Country	Region
Jimol	Early Miocene	Colombia	Guajira Peninsula (Caribbean coast)

Taxon	N° Specimens	Catalog number	Other number	Locality	Collection
Squaliformes					
Dalatias sp.	2 (T)	MUN-STRI-41205	—	220601	2013-14
Lamniformes					
†*Carcharocles* sp.	1 (T)	MUN-STRI-41158	—	290681	2013-14
Carcharhiniformes					
†*Hemipristis serra*	2 (T)	MUN-STRI-41206	—	290601	2013-14
†*Carcharhinus ackermanni*	1 (T)	MUN-STRI-41128	—	290820	2013-14
Carcharhinus spp.	4(T)	MUN-STRI-34776		290601	
Myliobatiformes					
Aetomylaeus sp.	1 (T)	MUN-STRI-37939	—	290602	2013-14
Rhinoptera sp.	7 (T)	MUN-STRI-41406	—	290602	2013-14

Geological Unit	Age	Country	Region
Undifferentiated Jimol/ Castilletes formations	Early Miocene	Colombia	Guajira Peninsula (Caribbean coast)

Taxon	N° Specimens	Catalog number	Other number	Locality	Collection
Pristiophoriformes					
Pristiophorus sp.	5 (T)	MUN-STRI-34788	—	290468	2013-14
Lamniformes					
Isurus sp.	13 (T)	MUN-STRI-37671	—	290468	2013-14
†*Paratodus* sp.	1 (T)	MUN-STRI-43742	—	290468	2013-14
†*Carcharocles* sp.	52 (T)	MUN-STRI-16900	—	290468	2013-14
Alopias sp.	2 (T)	MUN-STRI-43745	—	290468	2013-14
†*Anotodus* sp.	1 (T)	MUN-STRI-43740	—	290468	2013-14
Carcharhiniformes					
†*Hemipristis serra*	98 (T)	MUN-STRI-34790	—	290468	2013-14
Galeocerdo sp.	5 (T)	MUN-STRI-40377	—	290468	2013-14
†*Carcharhinus ackermanni*	1 (T)	MUN-STRI-43743	—	290468	2013-14
†*Carcharhinus gibbesii*	54 (T)	MUN-STRI- 43808	—	290468	2013-14
Carcharhinus spp.	498 (T)	MUN-STRI- 43807	—	290468	2013-14
Negaprion sp.	30 (T)	MUN-STRI-37681	—	290468	2013-14
†*Physogaleus* sp.	20 (T)	MUN-STRI-40378	—	290468	2013-14
Sphyrna sp.	7 (T)	MUN-STRI-43741	—	290468	2013-14
Myliobatiformes					
Aetobatus sp.	1 (T)	MUN-STRI-37638	—	290468	2013-14
Aetomylaeus sp.	10 (T)	MUN-STRI-43746	—	290468	2013-14
Rhinoptera sp.	3 (T)	MUN-STRI-37394	—	290468	2013-14
†*Plinthicus* sp.	3 (T)	MUN-STRI-40376	—	290468	2013-14

Geological Unit	Age	Country	Region
Ware Formation	Late Pliocene	Colombia	Guajira Peninsula (Caribbean coast)

Taxon	N° Specimens	Catalog number	Other number	Locality	collection
Carcharhiniformes					
Carcharhinus leucas	8 (T)	MUN-STRI-41121	—	390075	2013-14
Carcharhinus spp.	4 (T)	MUN-STRI-41120	—	390075	2013-14
Negaprion sp.	3 (T)	MUN-STRI-41119	—	390075	2013-14
Rhinopristiformes					
Pristis sp.	1 (T)	MUN-STRI-34512	—	390075	2013-14
Myliobatiformes					
Rhinoptera sp.	1 (T)	MUN-STRI-16343	—	390080	2013-14

Geological Unit	Age	Country	Region
Moin Formation	Late Pliocene-Early Pleistocene	Costa Rica	Limón Region (Caribbean coast)

Taxon	N° Specimens	Catalog number	Other number	Locality	Collection
Carcharhiniformes					
Carcharhinus limbatus	1 (T)	NMB S.A.1636	PPP-3255	Pueblo Nuevo	1998
Carcharhinus obscurus	1 (T)	NMB/to be assigend	PPP-3264	Pueblo Nuevo	1998
Carcharhinus sp.	1 (T)	NMB/to be assigend	PPP-948	Limón	1992
Carcharhinus sp.	1 (T)	NMB/to be assigend	PPP-950	Limón	1992
Sphyrna sp.	1 (T)	NMB/to be assigend	PPP-950	Limón	1992
Chondrichthyan indet.	1 (T)	NMB/to be assigend	PPP-950	Limón	1992

Geological Unit	Age	Country	Region
Chagres Formation	Late Miocene	Panama	Caribbean coast

Taxon	N° Specimens	Catalog number	Other number	Locality	Collection
Lamniformes					
†*Anotodus retroflexus*	1 (T)	PIMUZ-A/I-4627	—	Río Indio	2014
Carcharhiniformes					
Carcharhinus sp.	1 (T)	NMB/to be assigend	PPP-3218	Río Indio	1998
Rajiformes					
Rajidae Indet.	1 (T)	PIMUZ-A/I-4695		Río Indio	2014
Dasyatis sp.	1 (T)	NMB S.A.1558	PPP-3214	Miguel de la Borda	1998
Dasyatidae indet.	3 (T)	NMB S.A.1560a-c	PPP-3214	Miguel de la Borda	1998

Geological Unit	Age	Country	Region
Armuelles Formation	Pleistocene	Panama	Burica Peninsula (Pacific Coast)

Taxon	N° Specimens	Catalog number	Other number	Locality	Collection
Carcharhiniformes					
Carcharhinus leucas	1 (T)	NMB S.A.1548	PPP-047	Punta Burica, East coast	1986
Carcharhinus sp.	1 (T)	NMB/to be assigend	PPP-089	Monte Verde	1986

Geological Unit	Age	Country	Region
Burica Formation	Late Pliocene-Pleistocene	Panama	Burica Peninsula (Pacific Coast)

Taxon	N° Specimens	Catalog number	Other number	Locality	Collection
Lamniformes					
Carcharodon carcharias	1 (T)	PIMUZ-A/I 4670	STRI-31325	Punta Burica-620015	2011
Carcharhiniformes					
Carcharhinus limbatus	1 (T)	PIMUZ-A/I 4665	STRI-31328	Punta Burica- 620019	2006
Carcharhinus limbatus	1 (T)	PIMUZ-A/I 4668	STRI-31331	Punta Burica- 620013	2006
Carcharhinus limbatus	1 (T)	PIMUZ-A/I 4663	STRI-36693	Punta Burica- 620016	2013
Carcharhinus sp.	1 (T)	PIMUZ-A/I 4673	STRI-31327	Punta Burica-620015	2011
Carcharhinus sp.	1 (T)	PIMUZ-A/I 4672	STRI-36694	Punta Burica-620015	2013
Carcharhinus sp.	1 (T)	PIMUZ-A/I 4674	STRI-36696	Punta Burica-620015	2013
Carcharhinus sp.	1 (T)	PIMUZ-A/I 4675	STRI-36697	Punta Burica-620015	2013
Galeocerdo cuvier	1 (T)	PIMUZ-A/I 4667	STRI-31326	Punta Burica-620015	2011
Galeocerdo cuvier	1 (T)	PIMUZ-A/I 4664	STRI-36695	Punta Burica-620015	2013
Rhizoprionodon cf. *longurio*	1 (T)	PIMUZ-A/I 4671	STRI-31329	Punta Burica- 620019	1989
Rhizoprionodon cf. *longurio*	1 (T)	PIMUZ-A/I 4666	STRI-31335	Punta Burica- 620013	2006
Rhizoprionodon cf. *longurio*	1 (T)	PIMUZ-A/I 4669	STRI-31336	Punta Burica- 620016	2006
Negaprion brevirostris	1 (T)	PIMUZ-A/I 4662	STRI-36692	Punta Burica- 620016	2013
Negaprion brevirostris	1 (T)	PIMUZ-A/I 4676	STRI-31338	Punta Burica- 620018	2006
Myliobatiformes					
Rhinoptera sp.	1 (T)	PIMUZ-A/I 4677	—	Punta Burica- 620018	2017

Geological Unit	Age	Country	Region
Cayo Agua Formation	Early Pliocene	Panama	Bocas del Toro (Caribbean Coast)

Taxon	N° Specimens	Catalog number	Other number	Locality	Collection
Lamniformes					
†*Paratodus benedenii*	1 (T)	NMB S.A.1549	PPP-313	Cayo de Agua (Pta. Nispero)	1988
Carcharhiniformes					
Carcharhinus acronotus	1 (T)	NMB S.A.1550	PPP-047	Cayo de Agua	1988
Carcharhinus leucas	2 (T)	NMB/to be assigend	PPP-2237	Cayo de Agua	1995
Carcharhinus sp.	1 (T)	NMB/to be assigend	PPP-295	Cayo de Agua	1988
Galeocerdo cuvier	1 (T)	NMB/to be assigend	PPP-2245	Cayo de Agua	1995
Rhizoprionodon sp.	1 (T)	NMB/to be assigend	PPP-313	Cayo de Agua	1988
Rhizoprionodon sp.	1 (T)	NMB/to be assigend	PPP-355	Cayo de Agua	1988
Sphyrna sp.	1 (T)	NMB/to be assigend	PPP-2245	Cayo de Agua	1995

Geological Unit	Age	Country	Region
Escudo de Veraguas Formation	Late Pliocene	Panama	Caribbean coast

Taxon	N° Specimens	Catalog number	Other number	Locality	Collection
Carcharhiniformes					
Carcharhinus sp.	1 (T)	NMB/to be assigend	PPP-177	Escudo de Veraguas	1987
Carcharhinus sp.	1 (T)	NMB/to be assigend	PPP-178	Escudo de Veraguas	1987
Carcharhinus sp.	1 (T)	NMB/to be assigend	PPP-180	Escudo de Veraguas	1987
Carcharhinus sp.	1 (T)	NMB/to be assigend	PPP-362	Escudo de Veraguas	1988
Carcharhinus sp.	1 (T)	NMB/to be assigend	PPP-364	Escudo de Veraguas	1988
Rhizoprionodon sp.	1 (T)	NMB/to be assigend	PPP-1241	Escudo de Veraguas	1993
Chondrichthyan indet.	1 (T)	NMB/to be assigend	PPP-1241	Escudo de Veraguas	1993

Geological Unit	Age	Country	Region
Pucro Formation ?	Late Miocene	Panama	Darien (Pacific Coast)

Taxon	N° Specimens	Catalog number	Other number	Locality	collection
Carcharhiniformes					
Carcharhinus sp.	1 (T)	NMB/to be assigend	PPP-915	Río Chico	1991
Carcharhinus sp.	1 (T)	NMB S.A.1578	PPP-1609	Río Turquesa	1994
Sphyrna sp.	1 (T)	NMB S.A.1579	PPP-1612	Río Turquesa	1994
Myliobatiformes					
Dasyatis sp.	1 (T)	NMB S.A.1580	PPP-1607	Río Turquesa	1994
cf. *Dasyatis*	3 (T)	NMB S.A.1581a-c	PPP-1607	Río Turquesa	1994
cf. *Dasyatis*	1 (T)	NMB S.A.1582a-b	PPP-1607	Río Turquesa	1994
Styracura sp.	1 (T)	NMB S.A.1583	PPP-1612	Río Turquesa	1994
Dasyatidae indet.	1 (T)	NMB S.A.1584	PPP-1607	Río Turquesa	1994
Dasyatidae indet.	1 (T)	NMB S.A.1585a-e	PPP-1607	Río Turquesa	1994
Dasyatidae indet.	1 (T)	NMB S.A.1586	PPP-1607	Río Turquesa	1994
Mobula sp.	1 (T)	NMB S.A.1588	PPP-1607	Río Turquesa	1994
Chondrichthyan indet.	1 (Dt)	NMB/to be assigend	PPP-1607	Río Turquesa	1994

Geological Unit	Age	Country	Region
Shark Hole Point Formation	Early Pliocene	Panama	Caribbean coast

Taxon	N° Specimens	Catalog number	Other number	Locality	collection
Carcharhiniformes					
Sphyrna sp.	1 (T)	NMB/to be assigend	PPP-2226	Valiente Peninsula	1995

Geological Unit	Age	Country	Region
Swan Cay Formation	Early Pleistocene	Panama	Caribbean coast

Taxon	N° Specimens	Catalog number	Other number	Locality	collection
Carcharhiniformes					
Carcharhinus altimus	1 (T)	NMB/to be assigend	PPP-1995	Swan Cay	1995

Geological Unit	Age	Country	Region
Tuira Formation	Late Miocene-Pliocene	Panama	Darien (Pacific Coast)

Taxon	N° Specimens	Catalog number	Other number	Locality	collection
Orectolobiformes					
cf. *Ginglymostoma*	1 (T)	NMB S.A.1589	PPP-1625	Río Tupisa	1994
Carcharhiniformes					
Carcharhinus sp.	1 (T)	NMB S.A.1590	PPP-1149	Río Tupisa	1993
Carcharhinus sp.	1 (T)	NMB S.A.1591	PPP-1620	Río Tupisa	1994
Carcharhinus sp.	1 (T)	NMB/to be assigend	PPP-1723	Río Turquesa	1994
Rhizoprionodon sp.	1 (T)	NMB/to be assigend	PPP-1135	Río Turquesa	1993
Rhizoprionodon sp.	1 (T)	NMB S.A.1592	PPP-1163	Río Chico	1993
Rhizoprionodon sp.	1 (T)	NMB/to be assigend	PPP-1609	Río Turquesa	1994
Rhizoprionodon sp.	1 (T)	NMB S.A.1593	PPP-1620	Río Tupisa	1994
Rhizoprionodon sp.	1 (T)	NMB/to be assigend	PPP-1625	Río Tupisa	1994
Rhizoprionodon sp.	1 (T)	NMB/to be assigend	PPP-1625	Río Tupisa	1994
Sphyrna mokarran	1 (T)	NMB/to be assigend	PPP-1554	Río Chico	1994
Sphyrna cf. *S. zygaena*	1 (T)	NMB/to be assigend	PPP-1566	Río Chico	1994
Sphyrna sp.	1 (T)	NMB/to be assigend	PPP-1145	Río Tupisa	1993
Sphyrna sp.	1 (T)	NMB/to be assigend	PPP-1554	Río Chico	1994
Sphyrna sp.	1 (T)	NMB S.A.1594	PPP-1554	Río Chico	1994
Sphyrna sp.	1 (T)	NMB/to be assigend	PPP-1566	Río Chico	1994
Sphyrna sp.	1 (T)	NMB/to be assigend	PPP-1620	Río Tupisa	1994
Sphyrna sp.	1 (T)	NMB/to be assigend	PPP-1620	Río Tupisa	1994
Sphyrna sp.	1 (T)	NMB/to be assigend	PPP-1625	Río Tupisa	1994
Carcharhinidae indet.	2 (T)	NMB S.A.1595a/b	PPP-1142	Río Tupisa	1993
Carcharhinidae indet.	1 (T)	NMB S.A.1631	PPP-1554	Río Chico	1994
Carcharhinidae indet.	2 (T)	NMB S.A.1602	PPP-1554	Río Chico	1995
Carcharhinidae indet.	1 (T)	NMB S.A.1601	PPP-1620	Río Tupisa	1994
Carcharhinidae indet.	1 (T)	NMB/to be assigend	PPP-1627	Río Tupisa	1994
Rhinopristiformes					
Pristis sp.	1 (T)	NMB/to be assigend	PPP-1139	Río Turquesa	1993
Pristis sp.	1 (T)	NMB/to be assigend	PPP-1139	Río Turquesa	1993
Pristis sp.	1 (T)	NMB/to be assigend	PPP-1155	Río Chico	1993
Pristis sp.	1 (T)	NMB/to be assigend	PPP-1528	Río Tuira	1993
Pristis sp.	1 (T)	NMB/to be assigend	PPP-1554	Río Turquesa	1993
Rhynchobatus sp.	1 (T)	NMB/to be assigend	PPP-1142	Río Tupisa	1993
Rhynchobatus sp.	1 (T)	NMB S.A.1599	PPP-1566	Río Chico	1994
Rhynchobatus sp.	1 (T)	NMB/to be assigend	PPP-1609	Río Turquesa	1994

Myliobatiformes					
Dasyatis sp.	2 (T)	NMB S.A.1629a-b	PPP-1137	Río Turquesa	1993
Dasyatis sp.	3 (T)	NMB S.A.1611	PPP-1145	Río Tupisa	1993
Dasyatis sp.	7 (T)	NMB S.A.1614a-g	PPP-1164	Río Chico	1993
Dasyatis sp.	1 (T)	NMB S.A.1619	PPP-1155	Río Chico	1993
Dasyatis sp.	1 (T)	NMB S.A.1605	PPP-1627	Río Tupisa	1994
Dasyatis	12 (T)	NMB S.A.1608a-l	PPP-1625	Río Tupisa	1994
cf. *Dasyatis*	2 (T)	NMB S.A.1615	PPP-1132	Río Tupisa	1993
cf. *Dasyatis*	1 (T)	NMB S.A.1610	PPP-1134	Río Turquesa	1993
cf. *Dasyatis*	1 (T)	NMB S.A.1613	PPP-1139	Río Turquesa	1993
cf. *Dasyatis*	1 (T)	NMB S.A.1616	PPP-1145	Río Tupisa	1993
cf. *Dasyatis*	1 (T)	NMB S.A.1617	PPP-1149	Río Tupisa	1993
cf. *Dasyatis*	1 (T)	NMB S.A.1621	PPP-1163	Río Chico	1993
cf. *Dasyatis*	1 (T)	NMB S.A.1612	PPP-1528	Río Tuira	1994
cf. *Dasyatis*	13 (T)	NMb S.A.1625a-m	PPP-1566	Río Chico	1994
cf. *Dasyatis*	3 (T)	NMB S.A.1618a-c	PPP-1603	Río Turquesa	1994
cf. *Dasyatis*	13 (T)	NMB 1609a-m	PPP-1620	Río Tupisa	1994
cf. *Dasyatis*	5 (T)	NMB S.A.1626a-e	PPP-1625	Río Tupisa	1994
Dasyatidae indet.	5 (T)	NMB S.A.1624a-e	PPP-1149	Río Tupisa	1993
Dasyatidae indet.	1 (T)	NMB S.A.1620	PPP-1603	Río Turquesa	1994
Dasyatidae indet.	3 (T)	NMB S.A.1622a-c	PPP-1620	Río Tupisa	1994
Dasyatidae indet.	2 (T)	NMB S.A.1630a-b	PPP-1620	Río Tupisa	1994
Dasyatidae indet.	1 (T)	NMB S.A.1623	PPP-1625	Río Tupisa	1994
Dasyatidae indet.	1 (T)	NMB S.A.1604	PPP-1627	Río Tupisa	1994
Dasyatidae indet.	4 (T)	NMB S.A.1628a-d	PPP-1997	Río Turquesa	1994
Rhinoptera sp.	1 (T)	NMB/to be assigend	PPP-1625	Río Tupisa	1994
Mobula sp.	1 (T)	NMB S.A.1598	PPP-1625	Río Tupisa	1994
Chondrichthyan indet.	1 (Dt)	NMB S.A.1633	PPP-1132	Río Turquesa	1993
Chondrichthyan indet.	1 (Dt)	NMB/to be assigend	PPP-1139	Río Turquesa	1993
Chondrichthyan indet.	1 (Dt)	NMB S.A.1634	PPP-1164	Río Chico	1993
Chondrichthyan indet.	1 (Dt)	NMB/to be assigend	PPP-1603	Río Turquesa	1994
Chondrichthyan indet.	1 (T)	NMB/to be assigend	PPP-1605	Río Turquesa	1994
Chondrichthyan indet.	1 (T)	NMB/to be assigend	PPP-1607	Río Turquesa	1994
Chondrichthyan indet.	3 (Dt)	NMB/to be assigend	PPP-1620	Río Tupisa	1994
Chondrichthyan indet.	1 (Dt)	NMB/to be assigend	PPP-1620	Río Tupisa	1994
Chondrichthyan indet.	1 (Dt)	NMB/to be assigend	PPP-1620	Río Tupisa	1994
Chondrichthyan indet.	1 (Dt)	NMB S.A.1635	PPP-1625	Río Tupisa	1994
Chondrichthyan indet.	2 (T)	NMB/to be assigend	PPP-1625	Río Tupisa	1994

Geological Unit	Age	Country	Region
Manzanilla Formation	Late Miocene-Pliocene	Trinidad	Atlantic Ocean

Taxon	N° Specimens	Catalog number	Other number	Locality	collection
Carcharhiniformes					
Carcharhinus sp.	1 (T)	NMB/to be assigend	PPP-2676	San Jose River (San Jose Calcareous Silt)	1998

Geological Unit	Age	Country	Region
La Tejita Formation	Miocene	Venezuela	Margarita Island (Caribbean-Eastern Venezuela)

Taxon	N° Specimens	Catalog number	Other number	Locality	collection
Dasyatis sp.	1 (T)	AMU-CURS-782	PPP-2573	Margarita Airport	1995

Geological Unit	Age	Country	Region
Caujarao	Late Miocene	Venezuela	Western Venezuela

Taxon	N° Specimens	Catalog number	Other number	Locality	collection
Lamniformes					
†*Carcharocles* sp.	1 (T)	MTT-V-001	—	Near Tara-Tara	2015-2017
†*Anotodus* sp.	1 (T)	MTT-V-SN	—	Near Tara-Tara	2015-2017
Carcharhiniformes					
Hemipristis sp.	3 (T)	MTT-V-127	—	Near Tara-Tara	2015-2017
Galeocerdo sp.	2 (T)	MTT-V-117	—	Near Tara-Tara	2015-2017
Negaprion sp.	2 (T)	MTT-V-374	—	Near Tara-Tara	2015-2017
Rhizoprionodon sp.	1 (T)	MTT-V-381	—	Near Tara-Tara	2015-2017
†Physogaleus sp.	2 (T)	MTT-V-102	—	Near Tara-Tara	2015-2017
Sphyrna sp.	3 (T)	MTT-V-125	—	Near Tara-Tara	2015-2017
Rhinopristiformes					
Pristis sp.	1 (T)	MTT-V-391	—	Near Tara-Tara	2015-2017
Myliobatiformes					
Rhinoptera sp.	1 (T)	MTT-V-SN	—	Near Tara-Tara	2015-2017

Geological Unit	Age	Country	Region
Paraguaná Formation	Early Pliocene	Venezuela	Paraguaná Peninsula (Punta Cardón)

Taxon	N° Specimens	Catalog number	Other number	Locality	collection
Carcharhiniformes					
Carcharhinus sp.	1 (T)	AMU-CURS-778	—	Hotel Cardón-Club Miramar (Amuay Mb.)	2013
Sphyrna mokarran	1 (T)	AMU-CURS-779	—	Hotel Cardón-Club Miramar (Amuay Mb.)	2013
Carcharhinidae indet.	1 (T)	AMU-CURS-780	—	Hotel Cardón-Club Miramar (Amuay Mb.)	2013

Geological Unit	Age	Country	Region
La Tortuga Formation	Pleistocene	Venezuela	Margarita Island (Caribbean-Eastern Venezuela)

Taxon	N° Specimens	Catalog number	Other number	Locality	collection
Carcharhiniformes					
Mustelus sp.	1 (T)	AMU-CURS-781	PPP-2575	Tetas de María Guevara	1995
Myliobatiformes					
Dasyatis sp.	1 (T)	AMU-CURS-783	PPP-2575	Tetas de María Guevara	1995
Dasyatis sp.	1 (T)	AMU-CURS-784	PPP-3090	Tetas de María Guevara	1997
Dasyatidae indet.	1 (T)	AMU-CURS-785	PPP-2575	Tetas de María Guevara	1995
Dasyatidae indet.	1 (T)	AMU-CURS-786	PPP-2575	Tetas de María Guevara	1995
Dasyatidae indet.	1 (T)	AMU-CURS-787	PPP-2575	Tetas de María Guevara	1995
Dasyatidae indet.	1 (T)	AMU-CURS-788	PPP-2575	Tetas de María Guevara	1995
Dasyatidae indet.	1 (T)	AMU-CURS-789	PPP-2575	Tetas de María Guevara	1995
Dasyatidae indet.	1 (T)	AMU-CURS-790	PPP-2575	Tetas de María Guevara	1995
Dasyatidae indet.	1 (T)	AMU-CURS-791	PPP-2575	Tetas de María Guevara	1995
Dasyatidae indet.	1 (T)	AMU-CURS-792	PPP-2575	Tetas de María Guevara	1995
Dasyatidae indet.	1 (T)	AMU-CURS-793	PPP-2575	Tetas de María Guevara	1995
Dasyatidae indet.	1 (T)	AMU-CURS-794	PPP-2575	Tetas de María Guevara	1995
Dasyatidae indet.	1 (T)	AMU-CURS-795	PPP-2575	Tetas de María Guevara	1995
Dasyatidae indet.	1 (T)	AMU-CURS-796	PPP-2575	Tetas de María Guevara	1995
Rhinoptera sp.	1 (T)	N°/ to be assigend	PPP-2574	Margarita Airport	1995

Geological Unit	Age	Country	Region
San Gregorio Formation (Cocuiza Member)	Pleistocene	Venezuela	Western Venezuela

Taxon	N° Specimens	Catalog number	Other number	Locality	collection
Carcharhiniformes					
Carcharhinus spp.	20 (T)	AMU-CURS-S/N	—	Norte Casa Chiguaje	2017
Rhizoprionodon sp.	2 (T)	AMU-CURS-S/N	—	Norte Casa Chiguaje	2017
Sphyrna sp.	2 (T)	AMU-CURS-S/N	—	Norte Casa Chiguaje	2017
†*Hemipristis serra*	1 (T)	AMU-CURS-S/N	—	Norte Casa Chiguaje	2017
Galeocerdo sp.	2 (T)	AMU-CURS-S/N	—	Norte Casa Chiguaje	2017
Negaprion sp.	1 (T)	AMU-CURS-S/N	—	Norte Casa Chiguaje	2017
Myliobatiformes					
Rhinoptera sp.	10 (T)	AMU-CURS-S/N	—	Norte Casa Chiguaje	2017

Geological Unit	Age	Country	Region
Pisco	Late Miocene	Peru	Pacific

Taxon	N° Specimens	Catalog number	Other number	Locality	collection
Pristiophoriformes					
Pristiophorus sp.	4 (T)	RK-coll.nbr. 8/11	—	Agua de Lomas-Sacaco	1987
Carcharhiniformes					
Cephaloscyllium sp.	4 (T)	RK-coll.nbr. 7/683	—	Agua de Lomas-Sacaco	1987
Mustelus sp.	1 (T)	RK-coll.nbr. 7/684	—	Agua de Lomas-Sacaco	1987
Triakis sp.	30 (T)	RK-coll.nbr. 7/682	—	Agua de Lomas-Sacaco	1987
Sphyrna sp.	1 (T)	RK-coll.nbr. 7/681		Agua de Lomas-Sacaco	1987
Rhinopristiformes					
Pseudobatos sp.	2 (T)	RK-coll.nbr. 12/28	—	Agua de Lomas-Sacaco	1987
