# Supplementary material for: Shark and ray diversity in the Tropical America (Neotropics)—an examination of environmental and historical factors affecting diversity
Source: PeerJ. 2018 Jul 20;6:e5313. doi: 10.7717/peerj.5313 (PMC6055692; doi:10.7717/peerj.5313)
Supplement: Supplemental Information 7 — Data based on FishBase website (Froese & Pauly, 2017), Ocean Biogeographic Information System (OBIS: http://www.iobis.org/), and Table S2. Abbreviations: Eastern Atlantic (EA), Eastern Pacific (EP), Galapagos (Gal), Northwestern Atlantic (TNWA), North Brazil Shelf (NBS), Temperate Northern Atlantic (TNA), Temperate Northern Pacific (TNP), Tropical Eastern Pacific (TEP), Tropical Southwestern Atlantic (TSWA), Warm Temperate Southwestern Atlantic (WTSA), Warm Temperate Southeastern Pacific (WTSP), Western Atlantic (WA), and Western Pacific (WP). [file peerj-06-5313-s007.pdf]

Order	Family	Genus	Species	Eastern Central Pacific			Western Central Atlantic		
				TEP	Gal	WTSP	TNWA	NBS	TSWA
Squaliformes	Squalidae	*Squalus*	*Squalus bahiensis*						1
	Etmopteridae	*Etmopterus*	*Etmopterus benchleyi*	1					
			Etmopterus carteri				1		
			Etmopterus litvinovi			1			
			Etmopterus perryi				1		
			Etmopterus robinsi				1		
	Oxynotidae	*Oxynotus*	*Oxynotus caribbaeus*				1		
Pristiophoriformes	Pristiophoridae	*Pristiophorus*	*Pristiophorus schroederi*				1		
Squatiniformes	Squatinidae	*Squatina*	*Squatina david*				1		
Carcharhiniformes	Pentanchidae	*Apristurus*	*Apristurus canutus*				1		
		Bythaelurus	*Bythaelurus giddingsi*		1				
		Galeus	*Galeus antillensis*				1		
			Galeus cadenati				1		
			Galeus springeri				1		
		Parmaturus	*Parmaturus campechiensis*				1		
	Scyliorhinidae	*Schroederichthys*	*Schroederichthys maculatus*				1		
			Schroederichthys saurisqualus						1
			Schroederichthys tenuis					1	
		Scyliorhinus	*Scyliorhinus cabofriensis*						1
			Scyliorhinus hesperius				1		
			Scyliorhinus torrei				1		
			Scyliorhinus ugoi						1
	Proscylliidae	*Eridacnis*	*Eridacnis barbouri*				1		
	Triakidae	*Mustelus*	*Mustelus minicanis*				1		
		Triakis	*Triakis acutipinna*	1					
Rhinopristiformes	Rhinobatidae	*Pseudobatos*	*Pseudobatos prahli*	1					
Rajiformes	Arhynchobatidae	*Bathyraja*	*Bathyraja longicauda*			1			
		Notoraja	*Notoraja martinezi*	1					
	Gurgesiellidae	*Cruriraja*	*Cruriraja cadenati*				1		
		Fenestraja	*Fenestraja atripinna*				1		
			Fenestraja sinusmexicanus				1		
	Rajidae	*Amblyraja*	*Amblyraja hyperborea*	1					
		Breviraja	*Breviraja mouldi*				1		
		Dipturus	*Dipturus ecuadoriensis*	1					
		Leucoraja	*Leucoraja yucatanensis*				1		
		Malacoraja	*Malacoraja obscura*						1
		Rostroraja	*Rostroraja ackleyi*				1		
			Rostroraja bahamensis				1		
			Rostroraja cervigoni				1		
		Rajella	*Rostroraja eisenhardti*		1				
Torpediformes	Narcinidae	*Diplobatis*	*Diplobatis colombiensis*				1		
			Diplobatis guamachensis				1		
		Narcine	*Narcine leoparda*	1					
	Torpedinidae	*Torpedo*	*Torpedo andersoni*				1		
Myliobatiformes	Urotrygonidae	*Urobatis*	*Urobatis pardalis*	1					
			Urobatis tumbesensis	1					
		Urotrygon	*Urotrygon munda*	1					

		Urotrygon cimar	1						
		Urotrygon nana	1						
		Urotrygon reticulata	1						
		Urotrygon simulatrix	1						
		Urotrygon venezuelae					1		
Myliobatidae	*Myliobatis*	*Myliobatis chilensis*			1				
		Myliobatis ridens							1
Rhinopteridae	*Rhinoptera*	*Rhinoptera brasiliensis*							1
		Total spp.	**14**	**2**	**3**		**28**	**1**	**7**
