# Supplementary material for: Shark and ray diversity in the Tropical America (Neotropics)—an examination of environmental and historical factors affecting diversity
Source: PeerJ. 2018 Jul 20;6:e5313. doi: 10.7717/peerj.5313 (PMC6055692; doi:10.7717/peerj.5313)
Supplement: Supplemental Information 8 [file peerj-06-5313-s008.pdf]

Similarity coefficient values among the six marine provinces for sharks and rays

	EP-TEP	EP-Gal	EP-WTSP	WA-TNWA	WA-NBS
EP-Gal	0.89				
EP-WTSP	0.78	0.65			
WA-TNWA	0.37	0.41	0.38		
WA-NBS	0.39	0.38	0.33	0.96	
WA-TSWA	0.51	0.46	0.50	0.87	0.90

Similarity coefficient values among the six marine provinces including rays only.

	EP-TEP	EP-Gal	EP-WTSP	WA-TNWA	WA-NBS
EP-Gal	0.89				
EP-WTSP	0.86	0.72			
WA-TNWA	0.17	0.17	0.15		
WA-NBS	0.24	0.21	0.19	0.98	
WA-TSWA	0.32	0.21	0.29	0.78	0.90

Similarity coefficient values among the six marine provinces including sharks only.

	EP-TEP	EP-Gal	EP-WTSP	WA-TNWA	WA-NBS
EP-Gal	0.89				
EP-WTSP	0.75	0.61			
WA-TNWA	0.55	0.59	0.52		
WA-NBS	0.52	0.50	0.43	0.96	
WA-TSWA	0.65	0.62	0.62	0.91	0.92
