# Supplementary material for: Shark and ray diversity in the Tropical America (Neotropics)—an examination of environmental and historical factors affecting diversity
Source: PeerJ. 2018 Jul 20;6:e5313. doi: 10.7717/peerj.5313 (PMC6055692; doi:10.7717/peerj.5313)
Supplement: Supplemental Information 9 [file peerj-06-5313-s009.pdf]

Similarity coefficient values for the fossil assemblages

	EP-EMi	EP-MMi	EP-LMi	EP-P	EP-Pl	WA-EMi	WA-MMi	WA-LMi	WA-P
EP-MMi	0.82								
EP-LMi	0.77	1.00							
EP-P	0.78	1.00	0.97						
EP-Pl	0.37	0.86	0.95	1.00					
WA-EMi	1.00	0.86	0.86	0.79	0.71				
WA-MMi	0.77	0.89	0.89	0.82	0.73	0.99			
WA-LMi	0.77	0.94	0.89	0.89	0.88	0.92	0.96		
WA-P	0.77	0.94	0.86	0.90	0.88	0.90	0.94	0.99	
WA-Pl	0.56	0.83	0.86	0.91	0.91	0.84	0.87	0.97	0.98
