# Supplementary material for: Shark and ray diversity in the Tropical America (Neotropics)—an examination of environmental and historical factors affecting diversity
Source: PeerJ. 2018 Jul 20;6:e5313. doi: 10.7717/peerj.5313 (PMC6055692; doi:10.7717/peerj.5313)
Supplement: Supplemental Information 10 [file peerj-06-5313-s010.pdf]

References list of extant shark and rays from Tropical America

Acero PA, Tavera JJ, Anguila R, Hernández L. 2016. A New Southern Caribbean Species of Angel Shark (Chondrichthyes, Squaliformes, Squatinidae), Including Phylogeny and Tempo of Diversification of American Species. *Copeia* 104:577-585. DOI: 10.1643/CI-15-292

Acuña-Marrero D, Jiménez J, Smith F, Doherty PF, Hearn A, Green JR, Paredes-Jarrín J, and Salinas-de-León P. 2014. Whale Shark (*Rhincodon typus*) Seasonal Presence, Residence Time and Habitat Use at Darwin Island, Galapagos Marine Reserve. *PLoS One* 9:e115946. DOI: https://doi.org/10.1371/journal.pone.0115946

Aguilera OA. 1998. Los peces marinos del occidente de Venezuela. *Acta Biologica Venezuelica* 18:43-57.

Amezcua LF. 1996. *Peces demersales de la plataforma continental del Pacífico central de México*. México, D.F. : Instituto de Ciencias del Mar y Limnología, UNAM/CONABIO.

Bearez P. 1996. Lista de los peces marinos del Ecuador continental. *Revista de Biología Tropica*l 44:731-741.

Bonfil R. 1997. Status of shark resources in the southern Gulf of Mexico and Caribbean: implications for management. *Fisheries Research* 29:101-117. DOI: https://doi.org/10.1016/S0165-7836(96)00536-X

Bussing WA, and López M. 2009. Marine fish. *In: Wehrtmann IS, and Cortés J, eds. Marine* biodiversity of Costa Rica, Central America. Berlin: Springer, 453–458.

Bustamante C, Vargas-Caro C, and Bennett MB. 2014. Not all fish are equal: functional biodiversity of cartilaginous fishes (Elasmobranchii and Holocephali) in Chile. *Journal of Fish Biology* 85:1617-1633. DOI: 10.1111/jfb.12517

Castro JI. 2011. *The Sharks of North America*. Oxford: Oxford University Press.

Cervigón F. 2005. La ictiofauna marina de Venezuela: una aproximación ecológica. *Boletín del Instituto Oceanográfico de Venezuela* 44:3-28.

Cervigón F, and Alcalá A. 1999. *Los peces marinos de Venezuela. Tiburones y Rayas*. Edo. Nueva Esparta, Venezuela: Fundación Museo del Mar.

Chirichigno N, Vélez J. 1998. *Clave para identificar los peces marinos del Perú*. Lima: Instituto del Mar del Perú.

Chirichigno N, Cornejo RM. 2001. *Catálogo comentado de los peces marinos del Perú*. Lima: Instituto del Mar del Perú.

Compagno, LJ. 1984. *Sharks of the World: An annotated and illustrated catalogue of shark species known to date. Part 1: Hexanchiformes to Lamniformes*. Food and Agriculture Organization of the United Nations, Roma.

Compagno LJV. 1984. Sharks of the World. *An annotated and illustrated catalogue of shark species known to date. Part 2: Carcharhiniformes*. Roma: Food and Agriculture Organization of the United Nations.

Compagno LJV. 2002. *Bullhead, mackerel and carpet sharks (Heterodontiformes, Lamniformes and Orectolobiformes)*. Rome: Food and Agriculture Organization of the United Nations.

Compagno LJV, Dando M, Fowler S. 2005. *Sharks of the World*. Princeton: Princeton University Press.

Corgos A, Rosende-Pereiro A. 2016. First record of the whitetip reef shark, *Triaenodon obesus* from the coast of Jalisco, western Mexico mainland. *Marine Biodiversity Records* 9:66. DOI: 10.1186/s41200-016-0069-7

Cornejo R, Vélez-Zuazo X, González-Pestana A, Kouri J, Mucientes G. 2015. An updated checklist of Chondrichthyes from the southeast Pacific off Peru. *Check List* [S.l.], v. 11, n. 6, p. 1809, dec. 2015. Check List 11:1-7. DOI: http://dx.doi.org/10.15560/11.6.1809.

Cortés J. 2012. Marine biodiversity of an Eastern Tropical Pacific oceanic island, Isla del Coco, Costa Rica. *Revista de Biología Tropical* 60:131–185.

Cotto SA. 2001. *Guía De Identificación De Peces Marinos Del Mar Caribe De Nicaragua*. Nicaragua: Proyecto DIPAL II.

Daly-Engel TS, Baremore IE, Grubbs RD, Gulak SJB, Graham RT, Enzenauer MP. 2018. Resurrection of the sixgill shark *Hexanchus vitulus* Springer & Waller, 1969 (Hexanchiformes, Hexanchidae), with comments on its distribution in the northwest Atlantic Ocean. *Marine Biodiversity*. DOI: 10.1007/s12526-018-0849-x

Del Moral-Flores LF, Pérez-Ponce de León G. 2013. Diversidad de los peces Condrictios (tiburones, rayas y quimeras) de México. *Biodiversitas* 111:1–6.

Del Moral-Flores LF, Ramírez-Antonio E, Angulo A, Pérez-Ponce de León G. 2015. *Ginglymostoma unami* sp. nov. (Chondrichthyes: Orectolobiformes: Ginglymostomatidae): una especie nueva de tiburón gata del Pacífico oriental tropical.

Revista Mexicana de Biodiversidad 86:48-58. DOI: https://doi.org/10.7550/rmb.46192.

Ebert D. 2016. *Deep-sea cartilaginous fishes of the southeastern Pacific Ocean*. Rome: Food and Agriculture Organization.

Espinosa PH, Castro–Aguirre JL, Huidobro–Campos L. 2004. *Catálogo sistemático de tiburones (Elasmobranchii: Selachimorpha)*. Listados Faunísticos de México IX. México: Instituto de Biología, UNAM.

Estupiñán-Montaño C, Galván-Magaña F, Hacohen-Domené A, Estupiñán-Ortíz JF. 2016. First reports of Smalltooth sand tiger sharks, *Odontaspis ferox* (Elasmobranchii: Lamniformes: Odontaspididae), off the continental Ecuador. *Acta Ichthyologica et Piscatoria* 46:251-253.

Froese R, Pauly, D. 2017. FishBase. World Wide Web electronic publication. Available at: (accessed www.fishbase.org 10/2017 2015).

Gallo V, Cavalcanti MJ, da Silva RF, da Silva HM, Pagnoncelli D. 2010. Panbiogeographical analysis of the shark genus *Rhizoprionodon* (Chondrichthyes, Carcharhiniformes, Carcharhinidae). *Journal of Fish Biology* 76:1696-1713. DOI: doi:10.1111/j.1095-8649.2010.02609.x

Galván-Villa C, Ríos-Jara E, Bastida-Izaguirre D, Hastings PA, Balart EF. 2016. Annotated checklist of marine fishes from the Sanctuary of Bahía Chamela, Mexico with occurrence and biogeographic data. *ZooKeys* 554:139-157.

Geelhoed SCV, Janinhoff N, Verdaat JP. 2016. First visual record of a living basking shark Cetorhinus maximus in the Caribbean Sea. *Caribbean Journal of Science* 49:76-78. DOI: 10.18475/cjos.v49i1.a7

Grijalba-Bendeck M, Acevedo K. 2009. *Mitsukurina owstoni* Jordan (Chondrichthyes: Mitsukurinidae) primer registro para el Caribe Colombiano. *Boletín de Investigaciones Marinas y Costeras* 38:211-215.

Grove JS, Lavenberg RE. 1997. *Fishes of the Galápagos Islands*. Stanford, CA: Stanford University Press.

Guitart D. 1974-1978. *Sinopsis de los peces marinos de Cuba*. Tomos I al IV. La Habana, Cuba: Academia de Ciencias de Cuba. Instituto de Oceanología.

Hacohen-Domené A, Polanco-Vásquez F, Graham RT. 2016. First report of the whitesaddled catshark *Scyliorhinus hesperius* (Springer 1966) in Guatemala's Caribbean Sea. *Marine Biodiversity Records* 9:101. DOI: 10.1186/s41200-016-0103-9.

Hearn AR, Acuña D, Ketchum JT, Penaherrera C, Green J, Marshall A, Guerrero M, Shillinger G. 2014. Elasmobranchs of the Galapagos marine reserve. In: Denkinger J, and Vinueza L, eds. *The Galapagos Marine Reserve*: Springer International Publishing, 23-59.

Iglésias SP, Lecointre G, Sellos DY. 2005. Extensive paraphylies within sharks of the order Carcharhiniformes inferred from nuclear and mitochondrial genes. Molecular Phylogenetics and Evolution 34:569-583. DOI: https://doi.org/10.1016/j.ympev.2004.10.022

Jiménez-Prado P, Béarez P. 2004. *Peces Marinos del Ecuador continental/ Marine fishes of continental Ecuador*. Quito: SIMBIOE/NAZCA/IFEA , Tomo II.

Kondyurin VV, Myagkov NA. 1984. Sharks of the genus Squalus of the western Atlantic ocean. *Journal of Ichthyology* 24:118-121.

Kyne PM, Carlson JK, Ebert DA, Fordham SV, Bizzarro JJ, Graham RT, Kulka DW, Tewes EE, Harrison LR, Dulvy NK. 2012. The Conservation Status of North American, Central American, and Caribbean Chondrichthyans. Vancouver, Canada: IUCN Species Survival Commission Shark Specialist Group. Lamilla, J, Bustamante C. 2005) *Guía para el reconocimiento de tiburones, rayas y quimeras de Chile*. Oceana, 17, 1−80.

Last PR, Seret B, Naylor GJ. 2016. A new species of guitarfish, *Rhinobatos borneensis* sp. nov. with a redefinition of the family-level classification in the order Rhinopristiformes (Chondrichthyes: Batoidea). *Zootaxa* 4117:451-475. DOI: http://doi.org/10.11646/zootaxa.4117.4.1

Last PR, Weigmann S, Yang L. 2016. Changes to the nomenclature of the skates (Chondrichthyes: Rajiformes). In: Last PR, and Yearsley GK, eds. *Rays of the World*: Supplementary information. CSIRO Special Publication, 11–34.

Lessa R, Santana FM, Rincón G, Gadig OBF, El-Deir ACA. 1999. *Biodiversidade de elasmobrânquios do Brasil*. Recife: MMA/PROBIO, Necton, Elasmobrânquios.

Long DJ, McCosker JE, Blum S, Klapfer A. 2011. Tropical Eastern Pacific Records of the Prickly Shark, *Echinorhinus cookei* (Chondrichthyes: Echinorhinidae). *Pacific Science* 65:433-440. DOI: 10.2984/65.4.433

Long DJ, Sala E, Ballesteros E, Caselle JE, Friedlander AM, Klapfer A, Blum S, Constable HB. 2014. Summary of South American records of the smalltooth sand tiger shark Odontaspis ferox (Chondrichthyes: Odontaspidae), with the first record from Chilean waters. *Marine Biodiversity Records* 7:e67. DOI: 10.1017/S1755267214000700

Mantilla AL. 1998. Lista de Especies Elasmobranquias de Colombia. *Revista de Fenología y Anatomia* 1:1-42.

McCallister M, Ford R, Gelsleichter J. 2013. Abundance and Distribution of Sharks in Northeast Florida Waters and Identification of Potential Nursery Habitat. *Marine and Coastal Fisheries* 5:200-210. DOI: 10.1080/19425120.2013.786002

McEachran J, Fechhelm JD. 1998. *Fishes of the Gulf of Mexico*, Vol. 1: Myxiniformes to Gasterosteiformes Texas: University of Texas Press.

Mejía-Falla PA, Navia AF, Mejía-Ladino LM, Acero PA, Rubio EA. 2007. Tiburones y rayas de Colombia (Pisces Elasmobranchii): lista actualizada, revisada y comentada. *Boletín de Investigaciones Marinas y Costeras* 36:111-149..

Mejía-Falla P, Navia A, Puentes Granada V. 2011. *Guía para la identificación de especies de tiburones, rayas y quimeras de Colombia*. Bogota: Ministerio de Ambiente y Desarrollo Sostenible; Corporación para el Desarrollo Sostenible del Archipiélago de San Andrés, Providencia y Santa Catalina – CORALINA; Gobernación de San Andrés, Providencia y Santa Catalina, Fundación SQUALUS.

Mendoza A, Kelez S, Cherres WG, Maguiño R. 2017. The Largetooth Sawfish, *Pristis pristis* (Linnaeus, 1758), is not extirpated from Peru: new records from Tumbes. *Check List* 13. DOI: 10.15560/13.4.261

Menezes NA. 2011. Checklist dos peixes marinhos do Estado de São Paulo, Brasil. *Biota Neotropica* 11:1‑14.

Menezes N, L. de Figueiredo J, Buckup P, Moura R. 2003. *Catálogo das Espécies de Peixes Marinhos do Brasil*. Museu de Zoologia, Universidade dce São Paulo.

Menni RC, Stehmann MFW. 2000. Distribution, environment and biology of batoid fishes off Argentina, Uruguay and Brazil. A review. *Revista del Museo Argentino de Ciencias Naturales* 2:69‑109.

Navia AF, Mejía-Falla PA, Hleap JS. 2016. Zoogeography of Elasmobranchs in the Colombian Pacific Ocean and Caribbean Sea. *Neotropical Ichthyology* 14:e140134. DOI: 10.1590/1982-0224-20140134

Pequeño G. 1997. Peces de Chile. Lista sistemática revisada y comentada: addendum. *Revista de Biología Marina y Oceanografía* 32:77-94.

OBIS. 2017. Ocean Biogeographic Information System. Intergovernmental Oceanographic Commission of UNESCO. www.iobis.org.

Pikitch E, K , Chapman DD, Babcock EA, Shivji MS. 2005. Habitat use and demographic population structure of elasmobranchs at a Caribbean atoll (Glover's Reef, Belize). *Marine Ecology Progress Series* 302:187-197.

Robertson DR, Allen GR. 2015. Shorefishes of the Tropical Eastern Pacific: online information system. Version 2.0 In: Smithsonian Tropical Research Institute B, Panamá., editor.

Robertson DR, Tassell VJ. 2015. Shorefishes of the Greater Caribbean: online information system. Version 1.0. In: Smithsonian Tropical Research Institute B, Panamá, editor.

Rodriguez-Ferrer G, Wetherbee BM, Schärer M, Lilyestrom C, Zegarra JP, Shivji M. 2017. First record of the megamouth shark, *Megachasma pelagios,* (family Megachasmidae) in the tropical western North Atlantic Ocean. *Marine Biodiversity Records* 10:20. DOI: 10.1186/s41200-017-0117-y

Rosa RS, Gadig OBF. 2014. Conhecimento da diversidade dos Chondrichthyes marinhos no Brasil: a contribuição de José Lima de Figueiredo. *Arquivos de Zoologia*, 45:89‑104.

Rubio EA. 1987. *Lista sistemática de Peces Costeros y de Profundidad del Pacífico Colombiano*. Cali, Colombia: Centro de publicaciones Facultad de Ciencias, Universidad del Valle.

Schmitter Soto JJ, Vásquez Yeomans L, Aguilar Perera A, Curiel Mondragón C, Caballero Vázquez JA. 2000. Lista de peces marinos del Caribe mexicano. *Anales del Instituto de Biología Serie Zoología* 71:143-177.

Soares KD, Gomes UL, Carvalho MR. 2016. Taxonomic review of catsharks of the *Scyliorhinus haeckelii* group, with the description of a new species (Chondrichthyes: Carcharhiniformes: Scyliorhinidae). *Zootaxa* 4066:501-534. DOI: 10.11646/zootaxa.4066.5.1

Soto JMR. 2001. Annotated systematic checklist and bibliography of the coastal and oceanic fauna of Brazil. I. Sharks. *Mare Magnum* 1:51‑120.

Spier D, Gerum HLN, Bornatowski H, Contente R, Mattos NAS, Vilar CC, Spach HL. 2018. Ichthyofauna of the inner shelf of Paraná, Brazil: checklist, geographic distribution, economic importance and conservation status. *Biota Neotropica* 18.

Szpilman M. 2000. *Peixes Marinhos do Brasil - Guia Prático de Identificação*. Rio de Janeiro: Mauad Editora Ltda. p 288.

Tavares R. 2005. Abundancia y distribución de tiburones en el Parque Nacional Archipiélago de Los Roques y otras islas oceánicas venezolanas, 1997-1998. *Ciencias marinas* 31:441-454.

Tavares, R. (2005) Abundancia y distribución de tiburones en el Parque Nacional Archipiélago de Los Roques y otras islas oceánicas venezolanas, 1997-1998. Ciencias marinas, 31, 441-454.

Tavares R. 2009. Tiburones y rayas (Elasmobranquios): ¿Un recurso pesquero sobre-explotado en Venezuela? *INIAHOY* 4:71-77.

Tavares R, Arocha F. 2008. Species diversity, relative abundance and length structure of oceanic sharks caught by the Venezuelan longline fishery in the Caribbean Sea and western-central Atlantic. *Zootecnia Tropical* 26:489–503.

Uyeno T, Matsuura K, Fujii E. 1983. *Fishes trawled off Suriname and French Guiana*. Tokyo: Japan Marine Fishery Resource Research Center.

Viana STdf, Carvalho MRd, Gomes Ul. 2016. Taxonomy and morphology of species of the genus Squalus Linnaeus, 1758 from the Southwestern Atlantic Ocean (Chondrichthyes: Squaliformes: Squalidae). *Zootaxa* 4133:1–89. DOI: http://dx.doi.org/10.11646/zootaxa.4133.1.1

Voigt M, Weber D. 2011. *Field guide for sharks of the genus Carcharhinus*. München: Verlag Dr. Friedrich Pfeil.

Weigmann S. 2016. Annotated checklist of the living sharks, batoids and chimaeras (Chondrichthyes) of the world, with a focus on biogeographical diversity. *Journal of Fish Biology* 88:837-1037. DOI: 10.1111/jfb.12874
