# Supplementary material for: Shark and ray diversity in the Tropical America (Neotropics)—an examination of environmental and historical factors affecting diversity
Source: PeerJ. 2018 Jul 20;6:e5313. doi: 10.7717/peerj.5313 (PMC6055692; doi:10.7717/peerj.5313)
Supplement: Supplemental Information 11 [file peerj-06-5313-s011.pdf]

References list of fossil shark and rays from Tropical America

1. Carrillo-Briceño JD, Aguilera OA, Rodríguez F. 2014. Fossil Chondrichthyes from the central eastern Pacific Ocean and their paleoceanographic significance. *Journal of South American Earth Sciences* 51:76-90. DOI: http://dx.doi.org/10.1016/j.jsames.2014.01.001

2. Kindlimann R. 1990. Selacios del Terciario Tardío de Sacaco, Departamento de Arequipa. *Boletín de Lima* 69:91-95.

3. de Muizon C, Devries TJ. 1985. Geology and paleontology of late Cenozoic marine deposits in the Sacaco area (Peru). *Geologische Rundschau* 74:547-563.

4. Laurito CA. 1999. *Los seláceos fósiles de la localidad de Alto Guayacán (y otros ictiolitos asociados), Mioceno superior-Plioceno inferior de la Formación Uscari, provincia de Limón, Costa Rica*. San José: Laurito, C. (Ed.).

5. Aguilera O, Rodrigues de Aguilera D. 2001. An exceptional coastal upwelling fish assemblage in the Caribbean Neogene. *Journal of Paleontology* 75:732-742. DOI: 10.1666/0022-3360(2001)075<0732:AECUFA>2.0.CO;2

6. Aguilera OA, Ramos MIF, Paes ET, Costa SARF, Sánchez-Villagra MR. 2011. The Neogene tropical America fish assemblage and the paleobiogeography of the Caribbean region. *Swiss Journal of Palaeontology* 130:217-240. DOI: 10.1007/s13358-011-0020-9

7. Carrillo-Briceño JD, Argyriou T, Zapata V, Kindlimann R, Jaramillo CA. 2016. A new Early Miocene (Aquitanian) Elasmobranchii assemblage from the Gaujira Peninsula, Colombia. *Ameghiniana*:77-99. DOI: doi.org/10.5710/AMGH.26.10.2015.2931

8. Carrillo-Briceño JD, De Gracia C, Pimiento C, Aguilera OA, Kindlimann R, Santamarina P, Jaramillo C. 2015. A new Late Miocene chondrichthyan assemblage from the Chagres Formation, Panama. *Journal of South American Earth Sciences* 60:56-70. DOI: http://dx.doi.org/10.1016/j.jsames.2015.02.001

9. Laurito MCA, Calvo C, Valerio Al, Calvo A, Chacón R. 2014. Ictiofauna del mioceno inferior de la localidad de Pacuare de Tres Equis, formación río Banano, provincia de Cartago, Costa Rica, y descripción de un nuevo género y una nueva especie de scaridae. *Revista Geológica de América Central* 50:153-192. DOI: http://dx.doi.org/10.15517/rgac.v0i50.15121

10. Aguilera OA. 2010. *Peces Fósiles del Caribe de Venezuela*. Washington: Gorham Printing.

11. Underwood CJ, Mitchell SF. 2004. Sharks, bony fishes and endodental borings from the Miocene Montpelier Formation (White Limestone Group) of Jamaica. *Cainozoic Research* 3:157-165.

12. Casier E. 1966. Sur la faune ichthyologique de la Formation de Bissex Hill et de la Série océanique, de l'Ile de la Barbade, et sur l'âge de ces formations. *Eclogae Geologicae Helvetiae* 59:493-516.

13. Casier E. 1958. Contribution á l'étude des poissons fossiles des Antilles. *Mémoire Suisse de Paléontologie* 74:1-95.

14. Laurito CA. 1996. El género *Isistius* (Squalidae) en el Alto Guayacán. Formación Uscari (Mioceno Superior-Plioceno Inferior), provincia de Limón, Costa Rica. *Revista Geológica de América Central* 19-20:87-92. DOI: https://doi.org/10.15517/rgac.v0i19-20.8628

15. Landini W, Altamirano-Sierra A, Collareta A, Di Celma C, Urbina M, Bianucci G. 2017. The late Miocene elasmobranch assemblage from Cerro Colorado (Pisco

Formation, Peru). *Journal of South American Earth Sciences* 73:168-190. DOI: http://dx.doi.org/10.1016/j.jsames.2016.12.010

16. Suárez ME. 2015. Tiburones, rayas y quimeras (chondrichthyes) fósiles de Chile. In: Rubilar-Rogers D, Otero R, Vargas A, and Sallaberry M, eds. *Vertebrados Fósiles de Chile*. Santiago: Museo Nacional de Historia Natural, Chile, 17-33.

17. Carrillo-Briceño JD, González-Barba G, Landaeta MF, Nielsen SN. 2013. Condrictios fósiles del Plioceno Superior de la Formación Horcón, Región de Valparaíso, Chile central. *Revista Chilena de Historia Natural* 86:191-206.

18. Aguilera O, Luz Z, Carrillo-Briceño JD, Kocsis L, Vennemann TW, de Toledo PM, Nogueira A, Amorim KB, Moraes-Santos H, Polck MR, Ruivo MdL, Linhares AP, Monteiro-Neto C. 2017. Neogene sharks and rays from the Brazilian ‘Blue Amazon’. PLoS One 12:e0182740. DOI: 10.1371/journal.pone.0182740

19. Apolín J, González G, Martínez JM. 2004. Seláceos del Mioceno Superior de Quebrada Pajaritos (Piura, Perú). In: Perú SGd, editor. XII Congreso Peruano de Geología. p 401-404.

20. Pimiento C, González-Barba G, Ehret DJ, Hendy AJ, MacFadden BJ, Jaramillo C. 2013. Sharks and rays (Chondrichthyes, Elasmobranchii) from the late Miocene Gatun Formation of Panama. 87:755-774. DOI: https://doi.org/10.1666/12-117

21. Costa SAF, Richter M, Toledo PM, Moraes-Santos HM. 2009. Shark teeth from Pirabas formation (Lower Miocene), northeastern Amazonia, Brazil. *Boletin do Museo Paraense Emílio Goeldi, Ciencias Naturais* 4:221-230.

22. Reis MAFd. 2005. Chondrichthyan fauna from the Pirabas Formation, Miocene of northern Brazil, with comments on paleobiogeography. *Anuário do Instituto de Geociências* 28:31-58.

23. Carrillo-Briceño JD, Aguilera OA, De Gracia C, Aguirre-Fernández G, Kindlimann R, Sánchez-Villagra MR. 2016. An Early Neogene Elasmobranch fauna from the southern Caribbean (Western Venezuela). *Palaeontologia Electronica* 19.2.27A:1-32.

24. Laurito MCA, Valerio AL. 2008. Ictiofauna de la localidad de San Gerardo de Limoncito, Formación Curré, Mioceno Superior, cantón de Coto Brus, provincia de Puntarenas, Costa Rica. *Revista Geológica de América Central* 39:65-85. DOI: http://dx.doi.org/10.15517/rgac.v0i39.12249

25. Kruckow T, Thies D. 1990. Die Neoselachier der Paleokaribik (Pisces: Elasmobranchii). *Courier Forschungsinstitut Senckenberg* 119:1-102.

26. Portell RW, Hubbell G, Donovan SK, Green JL, Harper DA, Pickerill R. 2008. Miocene sharks in the Kendeace and Grand Bay formations of Carriacou, The Grenadines, Lesser Antilles. *Caribbean Journal of Science* 44:279-286.

27. Leriche M. 1938. Contribution à L'étude des Poissons fossils des pays riverains de la Méditerranée américaine, Venezuela, Trinité, Antiles, Mexique. *Mémoires de la Sociéte Paléontologique du Suisse* 61:1-52.

28. Marocco R, de Muizon C. 1988. Los Vertebrados del Neógeno de la Costa Sur del Perú: Ambiente sedimentario y condiciones de fosilización. *Bulletin de l'Institut Français d'études Andines* 17:105-117.

29. Alván A. 2007. Geología de Ocucaje: aportes en la sedimentología y paleontología de Lomas de Ullujaya (Ica, Perú). *Revista del Instituto de Investigaciones FIGMMG* 11:51-59.

30. Ehret DJ, Macfadden BJ, Jones DS, Devries TJ, Foster DA, Salas-Gismondi R. 2012. Origin of the white shark Carcharodon (Lamniformes: Lamnidae) based on recalibration of the Upper Neogene Pisco Formation of Peru. *Palaeontology* 55:1139-1153. DOI: 10.1111/j.1475-4983.2012.01201.x

31. Bianucci G, Di Celma C, Collareta A, Landini W, Post K, Tinelli C, de Muizon C, Bosio G, Gariboldi K, Gioncada A, Malinverno E, Cantalamessa G, Altamirano-Sierra A, Salas-Gismondi R, Urbina M, Lambert O. 2016. Fossil marine vertebrates of Cerro Los Quesos: Distribution of cetaceans, seals, crocodiles, seabirds, sharks, and bony fish in a late Miocene locality of the Pisco Basin, Peru. *Journal of Maps*: 1-10. DOI: 10.1080/17445647.2015.1115785

32. Iturralde-Vinent MA, Hubbell G, Rojas R. 1996. Catalogue of Cuban fossil Elasmobranchii (Paleocene to Pliocene) and paleogeographic implications of their lower to middle Miocene occurrence. *Boletín de la Sociedad Jamaicana de Geología* 31:7-21.

33. Perez VJ, Pimiento C, Hendy A, González-Barba G, Hubbell G, MacFadden BJ. 2017. Late Miocene chondrichthyans from Lago Bayano, Panama: Functional diversity, environment and biogeography. *Journal of Paleontology* 91:512-547. DOI: 10.1017/jpa.2017.5

34. Longbottom AE. 1979. Miocene shark's teeth from Ecuador. *Bulletin of the British Museum (Natural History) Geology* 32:57-70.

35. Pimiento C, Gonzalez-Barba G, Hendy AJW, Jaramillo C, MacFadden BJ, Montes C, Suarez SC, Shippritt M. 2013. Early Miocene chondrichthyans from the Culebra Formation, Panama: A window into marine vertebrate faunas before closure the Central American Seaway. *Journal of South American Earth Sciences* 42:159-170. DOI: http://dx.doi.org/10.1016/j.jsames.2012.11.005

36. Carrillo-Briceño JD, Maxwell E, Aguilera OA, Sánchez R, Sánchez-Villagra MR. 2015. Sawfishes and Other Elasmobranch Assemblages from the Mio-Pliocene of the South Caribbean (Urumaco Sequence, Northwestern Venezuela). *PLoS On*e 10:e0139230. DOI: https://doi.org/10.1371/journal.pone.0139230

37. Donovan SK, Gunter GC. 2001. Fossil sharks from Jamaica. *Bulletin of the Mizunami Fossil Museum* 28:211-215.

38. Nieves-Rivera AM, Ruiz-Yantín M, Gottfried MD. New Record of the Lamnid Shark Carcharodon megalodon from the Middle Miocene of Puerto Rico. *Caribbean Journal of Science* 39:223–227.

39. Aguilera OA, Rodrigues de Aguilera D. 2004. Giant-toothed White Sharks and Wide-toothed Mako (Lamnidae) from the Venezuela Neogene: Their Role in the Caribbean, Shallow-water Fish Assemblage. *Caribbean Journal of Science* 40:368-382.

40. MacPhee RDE, Iturralde-Vinent MA, Gaffney ES. 2003. Domo de Zaza, an Early Miocene vertebrate locality in South-Central Cuba, with notes on the tectonic evolution of Puerto Rico and the Mona Passage. *American Museum Novitates* 3394:1-42.

41. Collareta A, Lambert O, Landini W, Di Celma C, Malinverno E, Varas-Malca R, Urbina M, Bianucci G. 2017. Did the giant extinct shark Carcharocles megalodon target small prey? Bite marks on marine mammal remains from the late Miocene of Peru. *Palaeogeography*, *Palaeoclimatolog*y, *Palaeoecology* 469:84-91. DOI: http://dx.doi.org/10.1016/j.palaeo.2017.01.001

42. MacFadden BJ, Jones DS, Jud NA, Moreno-Bernal JW, Morgan GS, Portell RW, Perez VJ, Moran SM, Wood AR. 2017. Integrated Chronology, Flora and Faunas, and Paleoecology of the Alajuela Formation, Late Miocene of Panama. *PLoS One* 12:e0170300. DOI: 10.1371/journal.pone.0170300

43. Shimada K, Chandler RE, Lam OLT, Tanaka T, Ward DJ. 2016. A new elusive otodontid shark (Lamniformes: Otodontidae) from the lower Miocene, and comments on the taxonomy of otodontid genera, including the 'megatoothed' clade. *Historical Biology*:1-11. DOI: 10.1080/08912963.2016.1236795

44. Sánchez-Villagra MR, Burnham RJ, Campbell DC, Feldmann RM, Gaffney ES, Kay RF, Lozsán R, Purdy R, Thewissen JGM. 2000. A New Near-Shore Marine Fauna and Flora from the Early Neogene of Northwestern Venezuela. *Journal of Paleontology* 74:957-968. DOI: 10.1666/0022-3360(2000)074<0957:ANNSMF>2.0.CO;2

45. Laurito MCA. 2004. Ictiofauna de la Formación Punta Judas, Mioceno Medio, Provincia de Puntarenas, Costa Rica. *Brenesia* 62:57-73.

46. Purdy RW, Donovan SK, Pickerill RK, Dixon HL. 1996. Fish teeth from the Pleistocene of Jamaica. *Journal of Vertebrate Paleontology* 16:165-167.

47. Iturralde-Vinent M, Laurito C, Rojas R, Gutiérrez R. 1998. Myliobatidae (Elasmobranchii: Batomorphii) del Terciario de Cuba. *Revista de la Sociedad Mexicana de Paleontología* 8:135-145.
